# Minimal Peptoid Dynamics Inform Self-Assembly Propensity

**DOI:** 10.1021/acs.jpcb.3c03725

**Published:** 2023-12-01

**Authors:** Hamish W. A. Swanson, King Hang Aaron Lau, Tell Tuttle

**Affiliations:** Department of Pure and Applied Chemistry, University of Strathclyde, 295 Cathedral Street, Glasgow G1 1XL, U.K.

## Abstract

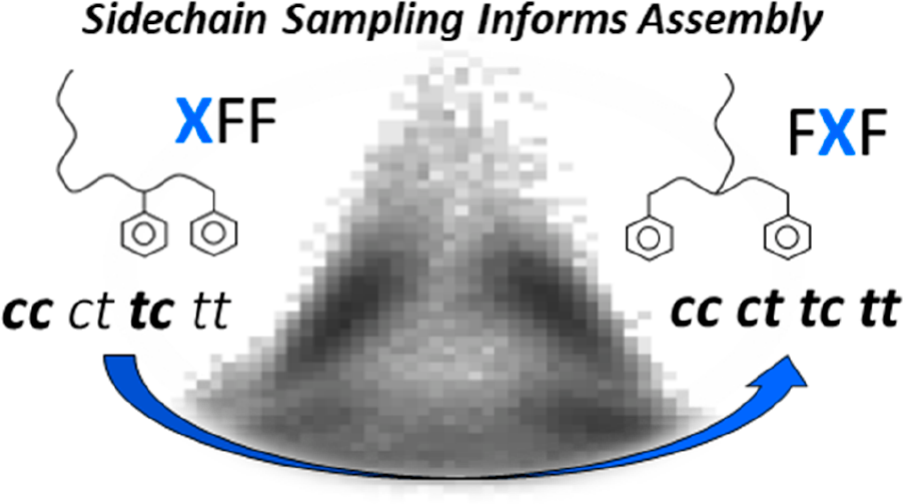

Peptoids are structural isomers of natural peptides, with side chain attachment at the amide nitrogen, conferring this class of compounds with the ability to access both *cis* and *trans* ω torsions as well as an increased diversity of ψ/φ states with respect to peptides. Sampling within these dimensions is controlled through side chain selection, and an expansive set of viable peptoid residues exists. It has been shown recently that “minimal” di- and tripeptoids with aromatic side chains can self-assemble into highly ordered structures, with size and morphological definition varying as a function of sequence pattern (e.g., XFF and FXF, where X = a nonaromatic peptoid monomer). Aromatic groups, such as phenylalanine, are regularly used in the design of minimal peptide assemblers. In recognition of this, and to draw parallels between these compounds classes, we have developed a series of descriptors for intramolecular dynamics of aromatic side chains to discern whether these dynamics, in a preassembly condition, can be related to experimentally observed nanoscale assemblies. To do this, we have built on the atomistic peptoid force field reported by Weiser and Santiso (CGenFF-WS) through the rigorous fitting of partial charges and the collation of Charmm General Force Field (CGenFF) parameters relevant to these systems. Our study finds that the intramolecular dynamics of side chains, for a given sequence, is dependent on the specific combination of backbone ω torsions and that homogeneity of sampling across these states correlates well with the experimentally observed ability to assemble into nanomorphologies with long-range order. Sequence patterning is also shown to affect sampling, in a manner consistent for both tripeptoids and tripeptides. Additionally, sampling similarities between the nanofiber forming tripeptoid, Nf-Nke-Nf in the *cc* state, and the nanotube forming dipeptide FF, highlight a structural motif which may be relevant to the emergence of extended linear assemblies. To assess these properties, a variety of computational approaches have been employed.

## 1 Introduction

**Figure 1:**
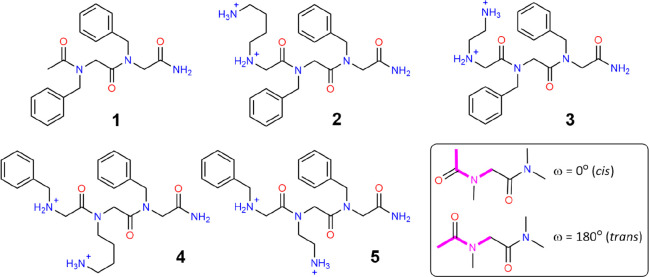
Molecules of interest in this study. Sequences **1** (Ac-Nf-Nf), **2** (Nk-Nf-Nf), **3** (Nke-Nf-Nf), **4** (Nf-Nk-Nf), and **5** (Nf-Nke-Nf) have been characterized experimentally by Lau et al.^36,37^ The inset in the box shows the definition of the *cis* and *trans* nomenclature for peptoids.

Peptoids are synthetic structural isomers of peptides with functional side chain attachment on the nitrogen atoms of the peptide bonds along a poly(N-substituted glycine) backbone, as opposed to the poly(C-substituted glycine) amino acid case. A consequence of this is that the backbone is achiral and can undergo *cis*/*trans* isomerization at the alpha-carbon (Figure 1).^1,2^ Peptoids have favorable properties, which make them appealing for diverse applications. Their resistance to proteolysis,^3^ enhanced cell permeability with respect to peptides,^4^ and properties that promote biocompatibility are highly favorable to therapeutic and biomedical applications.^5,6^ The convenience of the submonomer solid phase synthesis of peptoids^7^ in incorporating residues with functional side chains has promoted their use not only in combinatorial drug discovery^8^ but also for investigating the molecular variations required in multifarious polymer and materials applications, e.g., studying ion conduction of fuel cell membranes,^9^ as antifreezing agents^10^ and antifouling coatings,^11,12^ and in developing self-assembled nanostructures.^13,14^ More than 250 different side chains have been demonstrated,^15^ and this number is regularly increasing.^16^ Many peptoid nanoassemblies have been reported to date including nanosheets,^17−19^ nanotubes,^20^ superhelices,^21^ micelles,^22,23^ and polymersomes.^24^ The incorporation of lipophilicity to support molecular assembly has also subsequently been shown to modulate antimicrobial activity.^25−27^

Molecular dynamics (MD) simulations have enabled the understanding and development of a plethora of self-assembly systems. Especially for the study of small molecules, MD is efficient, given the small number of atoms or beads in each assembling molecule. As such, it has been feasible to screen the assembly of all 400 di- and 8000 tripeptide sequences^28,29^ at the coarse grain (CG) level of detail; this approach identified several previously unknown unprotected hydrogel forming tripeptides.^30^ With this large data set in hand, it was possible for Lampel et al. to generate design rules which describe tripeptide self-assembly: citing specifically the pairing of aromatic side chains and the inclusion of charged residues at terminal positions of the same charge (e.g., lysine at the N-terminus or aspartic acid at the C-terminus).^31^ Despite their short length, such “minimal” peptides up to four residues in length offer opportunities for a wide variety of therapeutic applications through interference with pathological assembly processes,^32^ microbe membrane disruption and lysis,^33^ as well as in terms of the mode of delivery through their ability to form gels and emulsions.^34,35^ Currently there have been limited studies on the assembly of such minimal peptoids,^36−39^ which is surprising given the wealth of discoveries made in the more established field of peptide self-assembly.^40−43^ It is our interest to understand how the assembly of minimal peptoids can enable the knowledge gained in the field of short-peptide assemblers to be imported into the field of peptoids.

In previous work Lau et al. investigated the assembly propensity of an acylated amide dipeptoid analogue of FF (Ac-Nf-Nf, **1**, Figure 1); intriguingly, this was found to form lamellar nanosheets,^36^ while the dipeptide is found to form the characteristic nanotube structures in both the termini unprotected form,^40^ with termini acylation/amidation,^44^ side chain heterochirality, and fluorination.^43^ Given the ubiquity of this nanostructure, its absence in the peptoid case is surprising. Furthermore, small-angle X-ray scattering (SAXS) measurements revealed the presence of noncrystalline packing which indicates a divergence from the crystallinity observed in the FF nanotube.

Subsequently in 2020 Lau et al. extended this work to study tripeptoid analogues of the tripeptide assemblers KFF and FKF (**2**–**5**, Figure 1),^37^ with side chains that are direct analogues of lysine (Nk, where “k” indicates it is a lysine analogue) and with shortened ethyl linkers (Nke, where “e” indicates it is lysine like with an *ethyl* linkage). It was found experimentally that Nk-Nf-Nf, **2**, and Nke-Nf-Nf, **3**, formed globular assemblies ∼45 nm in size. While Nf-Nk-Nf, **4**, formed larger vesicular structures of around ∼100 nm in size, strikingly Nf-Nke-Nf, **5**, gave a well-defined nanofiber many micrometers in length and with uniform width ca. 6 nm. The assemblies with the N(FXF) (where X = a nonbenzene containing side chain) motif were also found to be robust over a large pH range, of 3–9 for Nf-Nk-Nf and 3–11 for the nanofiber, indicating that hydrophobicity is critical to their assembly.

The divergence of the assembly between these peptoids and their peptide analogues is intriguing. To discover more molecules of interest and therein the nuances of short peptoid assembly, it is necessary to parametrize a CG force field for the screening of large data sets, and development in this direction is currently underway.^45,46^ However, a major hurdle to this aim is that atomistic models of peptoids are not as advanced as those for peptides, lacking in both a consistent parametrization of force fields and the number of residues described. Several force field families exist for the simulation of α-peptoids, and these are generally based on earlier models for peptides or general force fields for small molecules. The AMBER force field was first used by Armand to rationalize the helical handedness preference for polyproline type I (PPI) helices derived from (Nfes)_8_.^47^ Voelz et al. then performed a survey of AMBER force fields to assess which was best suited for the simulation of peptoids, by comparing the replication of low torsional minima from QM data obtained by Butterfoss et al.^48^ and experimental determined structures.^49^ The Generalized Amber Force Field (GAFF) performed best, an additional biasing potential was required to reproduce the correct φ torsion preference in Nfes residues, and subsequently this was successfully employed in *de novo* structure prediction of small peptoid molecules and the simulation of metal cation binding in Q-proline macrocycles.^50,51^ Mukherjee et al. also modified this force field for improved simulation of peptoid helices to reproduce QM and experimental results.^52^ This modified force field, dubbed GAFF-φ (also known as GAFF2), has subsequently been used to simulate porphyrin-modified peptoid helices and reproduce experimentally observed changes in helical structure.^53^ Recently, Harris et al. have built on GAFF-φ (also termed GAFF2) extending it to be applicable to 70 peptoid residues. A novel feature of this model is an emphasis on the reproduction of residue specific *K*_*cis*/*trans*_ values, setting a precedent for the incorporation of this peptoid intrinsic property into future parametrization schemes which will in turn enable greater distinction between peptoid models and their their amino acid counterparts.^54^

Other peptoid force fields include an OPLS model used by Park et al. to simulate polysarcosine chains.^55^ More recently, Hoyas et al. developed a DREIDING-based peptoid force field, dubbed PEPDROID, which can simulate the dynamics of both α- and β-peptoid backbones.^56^ The parametrization gave good reproduction of the QM results obtained by Butterfoss et al.^48^ as well as accurately reproducing the peptoid threaded loop secondary structure.^57^ Development within the Charmm-based family of peptoid force fields began with MFTOID, which is an adaption of the Charmm22 force field of proteins.^58^ Subsequently, the CGenFF-WS model was developed by Weiser and Santiso et al.^59^ based on the Charmm General Force Field (CGenFF).^60^ Both models enable the possibility of the *cis*/*trans* isomerization that exists in peptoid amide bonds, which is energetically not possible by using peptide parameters directly. However, the CGenFF-WS delivered improvements in the reproduction of peptoid φ/ψ backbone preferences as well as the to the ω angle torsion.

Adequate sampling of amide sequence states is an important consideration in peptoid force field applications. Recently Ferguson et al. used a MFTOID atomistic MD to rationalize the formation of peptoid helices and sheets and the solvent dependence of this hierarchical assembly pathway.^61^ The slow amide state switching, between 0.1 and 1 s,^1^ was overcome with extended simulation times. Alternatively, Edison et al. used *ad hoc* softening of torsional potentials of selected amide bonds to give an indication of conformational preference within simulated self-assembled nanosheets.^62^ Use of Replica Exchange Molecular Dynamics (REMD)^52,63−65^ and metadynamics^59,66−68^ has also been reported. Furthermore, Voelz et al. have also shown that enhanced-sampling MD simulations can be combined with sparse experimental NMR correlation data, through a Bayesian Inference of Conformational Populations method (BICePs),^69^ to accurately predict conformational populations in solution and thus rationalize cation binding in peptoid macrocyles.^51^ The peptoid simulation research space is a truly burgeoning field. Recently Alamdari et al. have shown the combination of DFT and metadynamics simulations to be a powerful platform from which to interrogate the potential energy surface (PES) of the peptoid backbone and in this effort rationalized the origins of conformational heterogeneity within such systems.^67,68^ In parallel Hwang et al. simulated pH-sensitive peptoid helices to provide insights into how the backbone *cis*/*trans* preferences change with side chain charge, giving agreement with experimentally observed changes as a function of this environmental property.^71^ The role of charged group sequencing in block amphiphilic peptoids was also investigated by Tsai et al.; this work provided molecule-specific insights into how sequencing can significantly impact resultant micellar structuring.^72^ To better inform enhanced sampling approaches to the study of peptoids sequences, Naleem et al. recently deployed and evaluated various machine learning (ML) schemes to identify the reaction coordinates and critical degrees of freedom which govern the cis α_D_ to trans α_D_ conformational transition in a sarcosine dipeptoid, providing a framework on which further work in this direction can be pursued.^73^

While the need for accurate modeling of peptoids is clearly growing, there has been little focus on the accurate determination of atomic partial charges in the CHARMM model family.^58,59^ Invariably, charges are taken from peptide fragments and elsewhere in existing peptoid force fields. In contrast, the CHARMM force field has a robust protocol for partial charge determination, which enables internal consistency between molecules and ensures that the expansion of the force field will not result in a decrease in accuracy. Notably this aspect of force field parametrization is “built in” and inherent to AMBER and DREIDING force field parametrization protocols, and therefore, this aspect of model design is sufficiently addressed in these model families.

We report in the present work an atomistic MD study using peptoid monomers for which a rigorous partial charge parametrization has been performed. Using the CGenFF approach,^60^ we demonstrate extension of a new molecular parametrization to a diverse selection of 31 monomers. To illustrate the applicability of our modified CGenFF-WS model, we also relate discrete intramolecular structuring at the preassembled stage to experimentally observed self-assembilies reported by Lau et al. with two MD methods: density functional theory (DFT) and through the evaluation of the absolute molecular entropies (AME). Additional insight into the behavior of peptoids is provided by novel evaluation of side chain aromatic group alignment and the distribution of different combinations of *cis*–*trans* conformations along the peptoid backbone.

## 2 Experimental Methods

### 2.1 Peptoid System Nomenclature

The rapid growth of peptoid research accompanied by the diversity of peptoid residues, including many side chains with no direct equivalents in natural amino acids, has resulted in various residue naming schemes lacking in consistency. With the goal of flexibly parametrizing diverse side chains, we have been motivated to develop a new general naming scheme for peptoid monomers to aid communication between peptoid researchers and those more familiar with peptide chemistry; furthermore, this standardization is able to circumvent the competing lexicon of peptoid monomer naming. This has been adopted in this paper (Figure 1), and so a brief overview is provided here. In this approach, the characteristic peptoid “N” is retained for familiarity. This is followed by a lowercase letter corresponding generally to the most closely related amino acid single letter code; e.g., the familiar residue termed Nlys or Nab is now simply Nk. On the other hand, completely novel peptoids with no relationship to amino acids can also easily fit into this scheme by, e.g., assigning letters not taken up by amino acids in the Latin, Greek, or other alphabets. Additional letters then specify side chain length, chirality, or atom substitution. Typically, we strive to make names of fewer than five characters in length for simplicity. Further explanation can be found online.^74^

### 2.2 Force Field Parametrization

We used the CGenFF bonded parameters reported by Weiser et al. to model the peptoid backbone torsions;^59^ where additional side chain parameters were required, we used the “initial guess” utility provided by The University of Maryland.^75^ In accordance with the CGenFF protocol, any parameters with penalty scores greater than 10 were reparametrized. The Force Field Toolkit (ffTK)^76^ was used for the refinement of angles and torsions (SI Section 2). We believe this is the most extensive compilation of peptoid relevant bonded terms within the CHARMM peptoid forcefield family to date, with sufficient diversity to simulate a total of 31 peptoid monomers.

The peptoid backbone and side chain partial charges were parametrized in a manner consistent with the CGenFF protocol.^60^ Specifically, this involved reproducing multiple TIP3P water–molecule interaction energies determined at the HF/6-31G(d) level of theory within ±0.2 kcal/mol, for structrues initially optimised at the MP2/6-31G(d) level. It was decided not to use CHARMM fixed values in cases where carbon and hydrogen atoms were in proximity to electronegative atoms, thereby maximizing the chemical detail which might be discerned from this parametrization scheme.

To generate possible partial charge sets, a grid search approach was taken. All possible permutations, from combinations of partial charges which summed to the molecular charge, were generated, with a grid size of 0.05*e*. Charge sets were then applied to the optimized structures, and the interaction energies were evaluated. Overestimation of the dipole moment by 1.2–1.5 times the magnitude at the HF/6-31G(d) level is required for a given partial charge set, and so this requirement was used to cut down preliminary partial charge sets. Adjustments were then made by hand to obtain the best agreement among these parameters. For simplicity, the peptoid backbone partial charges were parametrized and fixed, such that this operation was only necessary for new side chains. The data associated with this work are provided in SI Section 1.

### 2.3 Classical Molecular Dynamics

The slow time scale(s) over which peptoid amide bond isomerization occurs presents a challenge when screening for conformation-dependent properties. The number of unique 3D structures increases as a function of 4^*n*–1^, where *n* is the number of monomers in the peptoid chain.^77^ To preclude amide isomerization, we screened the dynamics of each amide sequence with a predefined *cis* (*c*) or *trans* (*t*) configuration for each backbone amide bond in the sequence (e.g., *cc*, *ct*, *tc*, and *tt*). We chose this approach to ensure conformational purity with respect to amide bond conformation. Each dilute system was composed of 25 molecules (4 × 4 × 4 nm^3^ box) which were solvated with TIP3P water to a concentration of ∼0.65 M; chlorine ions were added to ensure system neutrality as required for use of particle mesh Ewald (PME) electrostatics. By simulating a small population of dilute molecules, as opposed to single molecules, we sought to explore more of the potential energy surface (PES) and sample more conformations than a single molecule in the same duration of time. The van der Waals interactions were smoothly shifted at 0.8 nm to zero at a cutoff of 1.2 nm. A PME grid spacing of 0.1 Å was used to treat long-range electrostatic interactions beyond the short-range cutoff at 1.2 nm. All tripeptides were treated in the same manner and simulated using the Charmm36m model for proteins.^78^ For Ac-Nf-Nf acetonitrile molecules parametrized for CGenFF were used to make ∼70% acetonitrile: a 30% water solvent mixture which was then used to solvate the 25 molecules as consistent with the solvent environment in which the lamellar nanosheet formed (SI Section 2.3.8). All systems were built using Gromacs ver 2020.7^79^ and visualized using visual molecular dynamics (VMD).^80^ All systems were initially minimized for 10,000 steps; this was followed by heating to 298.15 K from 0 K in 10 K increments increasing every 1000 steps (80,000 steps total). Then the systems were equilibrated for (2,000,000 steps, 4 ns) with a Langevin thermostat, employing a damping factor of 5 ps^–1^ and a Langevin barostat with a reference pressure of 1.01325 bar. Following this, a production simulation using the same conditions was performed for 50 ns. NAMD ver. 2.15 was used for all multimolecule MD simulations.^81^ A 2 fs time step was used throughout. Transient aggregation occurred in the multimolecule peptoid and peptide simulations; though sustained aggregation was only observed for the dipeptide FF, as confirmed by solvent accessible surface area (SASA) analysis (SI Section 8, Figures S64–S68). Single molecule simulations of all peptoids and the peptides KFF, FKF, DFF, FDF, and FF were performed using Gromacs ver. 2020.7^79^ (see Supporting Information, Section 2.3 for details) to ensure that intermolecular interactions did not affect the intramolecular metrics used in this study. Analysis of ρ, λ, and η parameters (*vida infra*) confirmed that this was the case for all residues (Figures S69–S72).

### 2.4 Geometry Optimizations

Five structures of each amide backbone conformation of Ac-Nf-Nf (e.g., *cc*, *ct*, *tc*, and *tt*) were selected entirely at random over the 25 molecules simulated across the 2500 frames of the 50 ns simulation. All initial and optimized torsions and optimized energies are provided in SI Section 5 (Tables S40 and S41). These were optimized using the B97-3c method,^82^ which employs the DFT-D3 dispersion correction with Becke–Johnson damping,^83,84^ to provide insight into the relative energies between these different states. A conductor-like polarizable continuum model (CPCM) for acetonitrile was used. All DFT calculations were done using Orca ver. 5.0.3.^85^

### 2.5 Absolute Molecular Entropy (AME)

To assess the AME of the molecules of interest to this study, we decided to employ a method based on that recently described by Pracht et al.^86^ First the minimum-energy conformer was found using the Conformer-Rotamer Ensemble Sampling Tool (CREST)^87^ using the GFN-FF force field.^88^ This structure was then optimized at the B97-3c/def2-TVZP(D3) level of theory. The vibrational contribution to AME, *S*_msRHHO_ ,was then evaluated using the single-point Hessian (SPH)^89^ at the GFN2-xTB^90^ level using a scaling factor 0.97 using xtb.^91^ We found that this approach gave very similar vibrational contributions for the test cases described in the original report (SI Section 4 and Table S39). Then the conformational contributions to AME, *S*_conf_, and the *S̅*_msRHHO_ population correction were evaluated using CREST in entropy mode once again using the GFN-FF level of theory to limit computational expense. Note that if a new low global minimum-energy structure was found in this more rigorous conformer search, the process was restarted. This was done both for tripeptoids of interest and for all canonical amino acids with the XFF and FXF motif and some heterochiral tripeptides (*vida infra*).

### 2.6 ANI-1ccx Molecular Dynamics

To both complement the classical MD component of this study and to further interrogate the dynamic differences between our molecules of interest, we performed 1 ns simulation on each molecule of interest in the each amide conformation state using the CCSD(T)/CBS extrapolated ANI potential (ANI-1ccx) to evaluate molecular energies and atomic forces.^92^ The Atomic Simulation Environment (ASE) Python module was used to perform these simulations.^93^ A 1 fs time step was used while a Langevin thermostat was used to maintain a temperature of 298.15 K using a friction coefficient of 0.002 fs^–1^. Atomic velocities were initially fit to a Maxwell–Boltzmann distribution.

### 2.7 Phenyl Side Chain Alignment Analysis: ρ, λ, and η

We hypothesized that the intramolecular organization of side chains in our sequences would shed light on the relationship between a peptoid sequence and its peptide analogue as well as on the differences in side chain dynamics between amide pseudo-stereoisomers (e.g., different ω angle conformations for the same molecule, such as *cis–cis* (*cc*) or *cis–trans* (*ct*) conformations). The minimal peptoid systems studied by Lau et al. all contained phenyl side chains, and it was experimentally observed that specific π–π interactions were present in Ac-Nf-Nf and Nf-Nke-Nf.^36,37^

At the same time, the FF dipeptide constitutes the core self-assembly motif of both amyloid fibers and a range of peptide systems (e.g., FF, XFF, FXF, and FFX) including the hydrophobic component of peptide nanotube assembly, as first reported by Gazit et al.^40^ This was also seen as central to the tripeptide assembly design rules produced by Lampel et al.,^31^ and Fredrix et al. have shown that changes in aromatic side chain orientations are critical to the assembly mechanism of the dipeptide FF in a coarse-grained mechanistic assembly study.^28^ Additionally, we are inspired to take an intramolecular view of assembling molecules by the increasing number of examples of heterochiral peptides for which changes in backbone stereochemistry significantly alter assembly outcomes.^24,32,94−96^ Here, side chain functionality and hence intermolecular interaction types are conserved, but their presentation to the environment, through conformation, differs. Intramolecular metrics seem to be best placed to deconvolve such systems.

Given the preeminence of this aromatic functionality, we focused on internal intramolecular π–π organization (e.g., face to face and edge to face) in the assemblers of interest to probe if this could provide insight into the formation of extended nanostructures. We define a ratio, ρ, as a first metric for assessing the geometric degree of alignment of aromatic side chains, which can be applied to both peptoids and peptides:
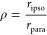
1where *r*_ipso_ and *r*_para_ correspond to the distances between ipso and para carbons, respectively, on consecutive benzene side chains. A value of *r* = 1.0 corresponds to an exact face to face alignment of the side chains.

**Figure 2:**
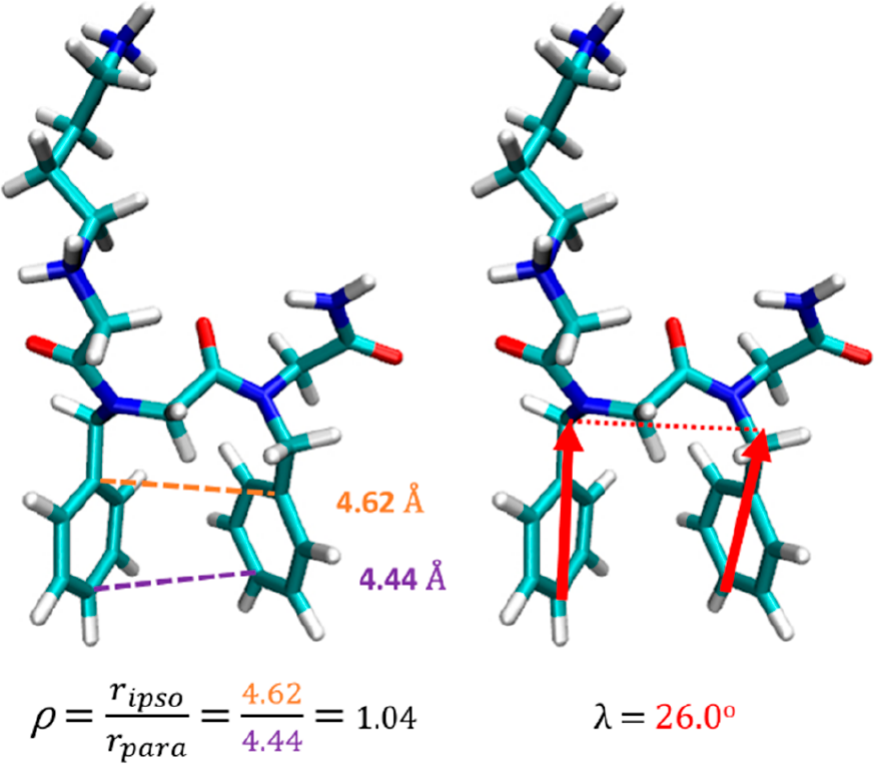
Illustration of ρ and λ parameters used to describe the relative alignment of benzene side chains in a molecule of Nk-Nf-Nf in the *cis*–*trans* (*ct*) conformation. *Ρ* is the ratio of ipso–ipso and para–para distances, and λ is the torsion between the aromatic rings. When λ = ±180° the rings are deemed to be coplanar.

We further define λ, the dihedral angle between the C1_para_–C1_ipso_–C2_ipso_–C2_para_ carbons, to add torsional information (Figure 2). With these two variables, it is possible to produce a 2D ρ–λ surface to describe the arrangement of phenylic side chains. It was identified that sampling in λ is correlated to that of φ/ψ, and an in-depth analysis of this interrelation is outlined in SI Section 2.3.4. Importantly, to quantify the extent to which a molecule explores the ρ–λ surface in a simulation, the change in the torsion, Δλ, was compared between the current frame *i* and the next frame *i* + 1 (eq 2). We consider that a “switch” in intramolecular aromatic ring organization has taken place when Δλ≥ 20°. The number of switches per molecule is summed, and an average is taken across the 25 molecules within the simulation, *x̅*_*λ*_ (eq 3). Note: 360° is added to any λ value less than 0°.
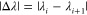
2
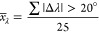
3

The number of switches across the 25-molecule population for a given amide state is generally consistent, and so representing these as an average was deemed acceptable (SI Section 3.3). To further validate this strategy, we performed bootstrapping analysis of the Δλ values to generate bootstrapped *x̅*_λ_ values (*n* = 1000). These were found to be normally distributed and generally occupied a narrow range with small bootstrap standard deviations. This confirms that the metric is robust with respect to dynamic differences between molecules within a given population (Tables S33 and S34 and Figures S54–S58). We hypothesize that the more the λ side chain torsional space is sampled, the more efficiently aromatic units will be accommodated into well-defined assembled hydrophobic domain. As backbone conformations exist as an ensemble, we propose that side chain exploration independent from amide backbone structuring would be important to accommodate assembled arrangements. Moreover, the homogeneity of *x̅*_λ_ across different amide state combinations across residues can be computed with η, defined as the ratio of the maximum (*x̅*_λmax_) and minimum (*x̅*_λmin_) values across the amide states (eq 4).
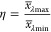
4When |®_λmax_ – *x̅*_λmin_| → 0, then η →1.0. λ exploration is deemed to be homogeneous when η ≈ 1.0. Conversely, when η ≫ 1.0, the backbone exploration is thought to be inhomogeneous.

## 3 Results and Discussion

### 3.1 Dipeptoid Assembly Predisposition

**Figure 3:**
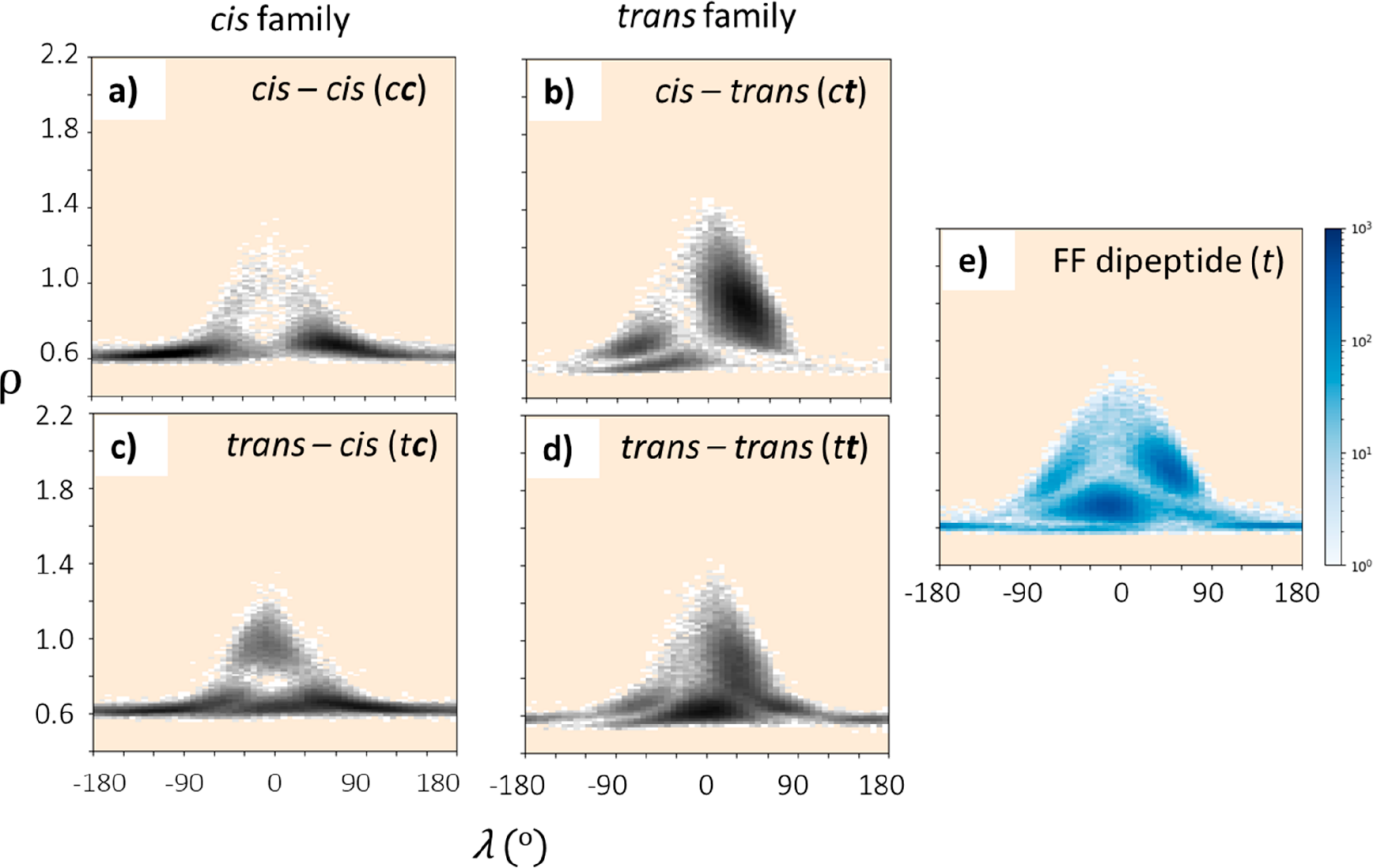
(a–d) ρ vs λ plots for Ac-Nf-Nf in both the *cis–cis* (*cc*), *cis–trans* (*ct*), *trans–cis* (*tc*), and *trans–trans* (*tt*) conformations. (e) Furthermore, the same plot for the dipeptide FF (*t*) is included to compare sampling preferences in the ρ/λ surface. In all cases, the beige background is used to show regions that are unsampled in the trajectory. Each plot is composed of every ρ vs λ combination for every frame, 2500 frames total, and for all 25 molecules, 60 bins are used for the histogram.

We first studied the assembly of Ac-Nf-Nf, which was recently reported to undergo self-assembly in 70% acetonitrile to form free-floating lamellar structures consisting of 5–10 layers of dipeptoids.^36^Figures 3a–d show the ρ vs λ plots characterizing benzyl stacking interactions between the aromatic side chains of the Nf residues and clearly demonstrate the significant impact of amide backbone conformation on these properties. Four possible amide conformation contributions are shown (i.e., *cis–cis* (*cc*), *cis–trans* (*ct*), *trans–cis* (*tc*), and *trans–trans* (*tt*)). Values of ρ > 0.8 indicate favorable “face to face” or “parallel displaced” π–π interactions (except for unstable exact face to face alignment around ρ = 1.0) while deviations away from λ = 0° indicate in-plane misorientation between the side chain benzenes.

It is striking that the ρ–λ surface for the *cis* pair (i.e., *cc* and *tc* states; Figure 3a,c) is very different from those of the *trans* pair (i.e., *tt* and *ct* states; Figure 3b,d), with the former occupying two highly populated regions at a low degree of alignment (ρ ∼ 0.6, λ ≳ ±60°) and the latter sharing two consistent domains, one at a higher side chain alignment than the other (i.e., ρ ∼ 0.8, λ ∼ 60° vs ρ ∼ 0.7, λ ∼ −90°). The *tt* state also exhibit an additional domain (ρ ∼ 0.7, λ ∼ 0°). This pairing of backbone states most likely reflects the importance of backbone state between Nf-Nf over that between Ac-Nf because the small acetyl capping group has less impact over spatial sampling of side chains (i.e., *cc* and *tc* are dynamically similar, as are *tt* and *ct*).

Interestingly, we find similar sampling geometries between the *trans* pair and the well-characterized FF dipeptide system (Figure 3b,d and Figure 3e). Given the potential for very similar side chain dynamics for FF and Ac-Nf-Nf, and therefore similar hydrophobic region organization (e.g., optimal π–π interactions), it is intriguing that FF forms crystalline nanotubes when diluted in water from hexafluoroisopropanol, an organic solvent that disrupts hydrogen bonding,^40^ while Ac-Nf-Nf forms an amorphous nanosheet in 70% acetonitrile.

**Figure 4:**
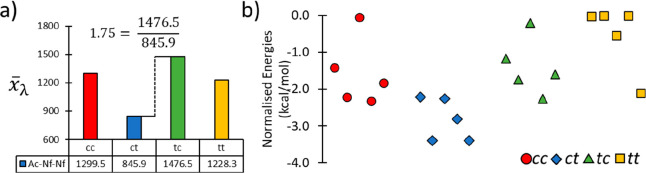
(a) *x̅*_λ_ values for each amide sequence set of Ac-Nf-Nf from which the homogeneity parameter, η, is calculated as illustrated. (b) Normalized energies of Ac-(Nf)_2_ structures in CPCM acetonitrile solvent at the B97-3c level of theory for 5 conformers per amide sequence state. These occupy an energy range <4 kcal/mol, the closeness of which confirms that an ensemble of states will exist in solution.

To obtain further insight, we analyzed the conformational energies of Ac-Nf-Nf. Five randomly selected conformers of each amide state were optimized at the B97-3c level of theory using acetonitrile as the CPCM solvent to match experiment. It was found that these structures occupied a normalized energy range of <4 kcal/mol and that the *cis* and *trans* pairs are generally similar in energy (Figure 4b, SI Section 5), which suggests that no single amide bond conformation is likely to be dominant and that an interchanging ensemble may exist in solution. This result is expected given the wide number of ω, ψ, and φ states which are accessible to achiral residues such as Nf.^67^

Experimentally, Ac-Nf-Nf adopts a *cis*-amide crystal when dried slowly from a low-volatility DMSO:water solvent mixture,^36^ and this present study now calculates low ρ values and laterally extended benzene rings for this structure (ρ ∼ 0.651 and λ ∼ ±57.2°; see SI Section 6). This is intriguing given that DFT energies indicate that an ensemble should exist in solution; indeed, elsewhere many reported peptoid crystals adopt the *cis*-amide conformation.^97^ Such organization contrasts with the crystal structure obtained for FF by Görbitz et al.^98^ where the *trans* backbone would preclude such structuring and for which only one ρ–λ surface exists.

Applying the analysis of the homogeneity parameter yields a large variation of η = 1.75, which indicates a large difference in aromatic domain rigidity between the *ct* and *tc* conformation (Figure 4a). Taken together, the solution phase Ac-Nf-Nf system has an ensemble of *cis* and *trans* pairs, as implied by close DFT energies, which possess varied aromatic side chain structures, as indicated by the distinct ρ–λ surfaces. Consequently, upon assembly, this ensemble would be ill suited to accommodate one another in the formation of hydrophobic domains with mixed amide backbone populations. This kind of poor long-range tessellation would yield an amorphous assembly structure, as observed by experiment.^36^ In contrast, amide conformations in FF are constrained to the *trans* state, and so purity in side chain structuring allows for the well-defined benzene orientations observed in their crystal structures.

### 3.2 Tripeptoid Assembly Predisposition

**Figure 5:**
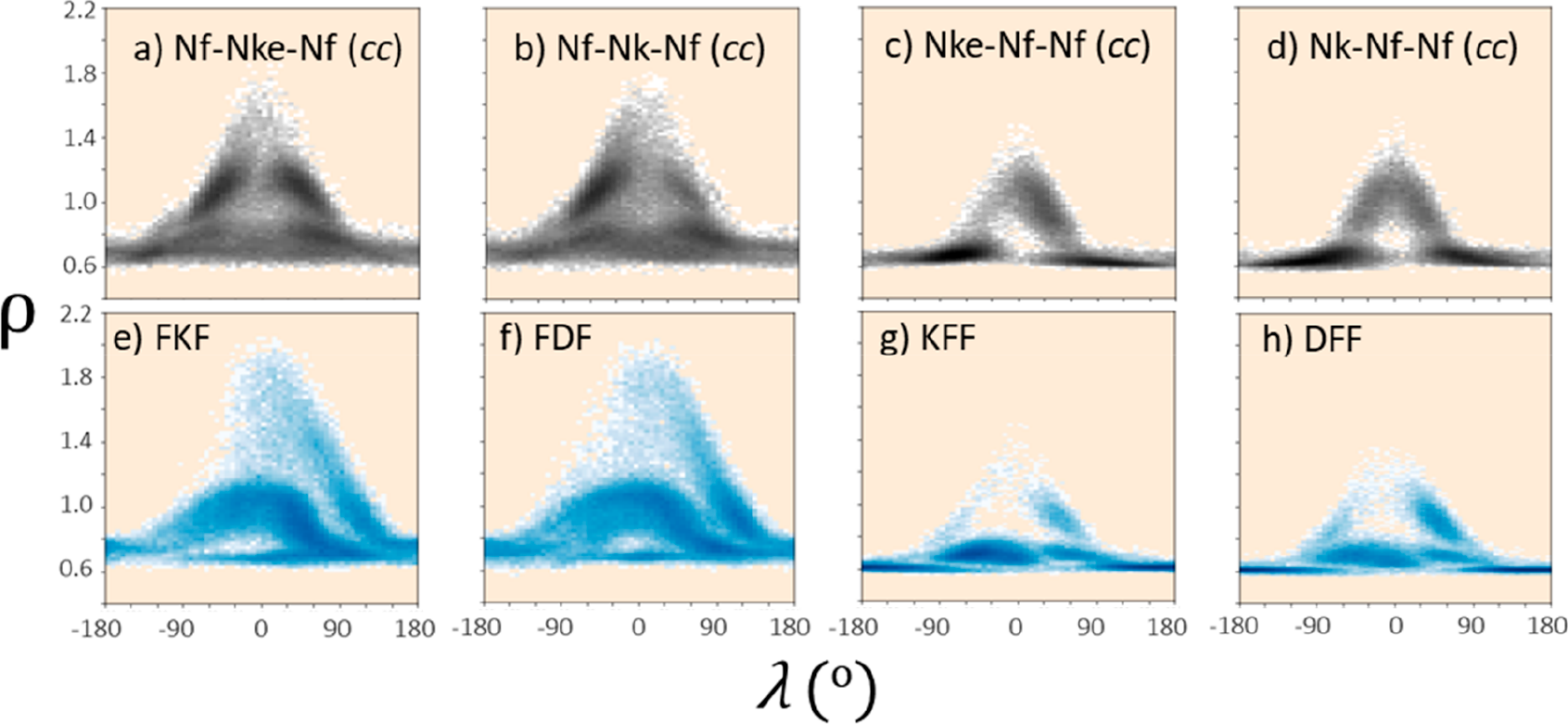
(a–d) ρ vs λ surfaces for tripeptoids **2**–**5** in the *cis–cis* state as well as (e–h) for the tripeptides FDF, FKF, DFF, and KFF. The beige background corresponds to regions unsampled in the trajectory. Assembly conditions and morphologies for the tripeptides can be found in ref (31). A dependence of sampling on sequence pattern, which is conserved between peptoids and peptides, is revealed through this analysis. Plots of all backbone combinations are detailed in SI Section 3.1.

The previous section indicates that our approach is sufficient to distinguish the dynamic features of the dipeptoid system. For the case of tripeptoid assembly, recently characterized experimentally by Lau et al.,^37^ side chain dynamics are also found to be strongly correlated to amide backbone conformation, as revealed through analysis of ρ vs λ surfaces. The results for all backbone combinations are detailed in SI Section 3.1. For illustration, the *cc* conformations are shown in Figure 5a–d, which reveal a distinct difference in sampling between assemblers with the Nf-X-Nf (Figure 5a,b) and X-Nf-Nf (Figure 5c,d) sequence motifs, where X is the cationic, lysine-like Nk or Nke (Figure 1).

Specifically in the case of Nf-X-Nf, the surface qualitatively seems to be more readily explored within the 50 ns time scale trajectory over the population of simulated molecules. To verify our approach, we applied this analysis to some well-known tripeptide assemblers and found that sequence patterning has a similar impact on sampling with the analogous FXF qualitatively exploring more of the surface (FKF and FDF, Figure 5e,f) than the XFF pattern (KFF and DFF, Figure 5g,h). These findings are corroborated by previous work of Lampel et al. in which the assembly characteristics of C-terminal amidated tripeptides composed of tyrosine (Y), phenylalanine (F), and aspartic acid (D) with sequence patterns XFF, FXF, and FFX. Both FXF trimers failed to form assemblies at pH 8 (e.g., YDF-NH_2_ and FDY-NH_2_), whereas all other patterns assembled with morphologies ranging from amorphous aggregates to opaque nanofiber gels. In this work the torsion, defined as λ in our study, was characterized by MD simulations, and it was found that the FXF pattern sequences had no well-defined torsions, whereas in contrast the XFF and FFX patterns exhibited defined sampling in λ. Therefore, for dual-aromatic trimer sequences, this “exploratory” property or disorder in aromatic torsions is contingent on patterning and relevant both in the peptide and peptoid domains.

In comparing to peptide systems, there are also suggestive correlations between both the tripeptoid Nf-Nke-Nf and the dipeptide FF. Both are experimentally observed to assemble into higher order linear morphologies, and our analysis now reveal that both sample similar ρ vs λ regions (e.g., ρ ∼ 1.0 and λ ± 60°, contrast Figure 5a and Figure 3e). While these ρ vs λ conformations may be important in promoting assemblies with long-range order, they may not be a sufficient condition because their sampling is also observed in Nf-Nk-Nf (*cc* and *tc*), Nke-Nf-Nf (*ct* and *tc*), and Nk-Nf-Nf (*ct* and *tt*) which do not form such well-defined structures (Figures S45 and S46).

**Figure 6:**
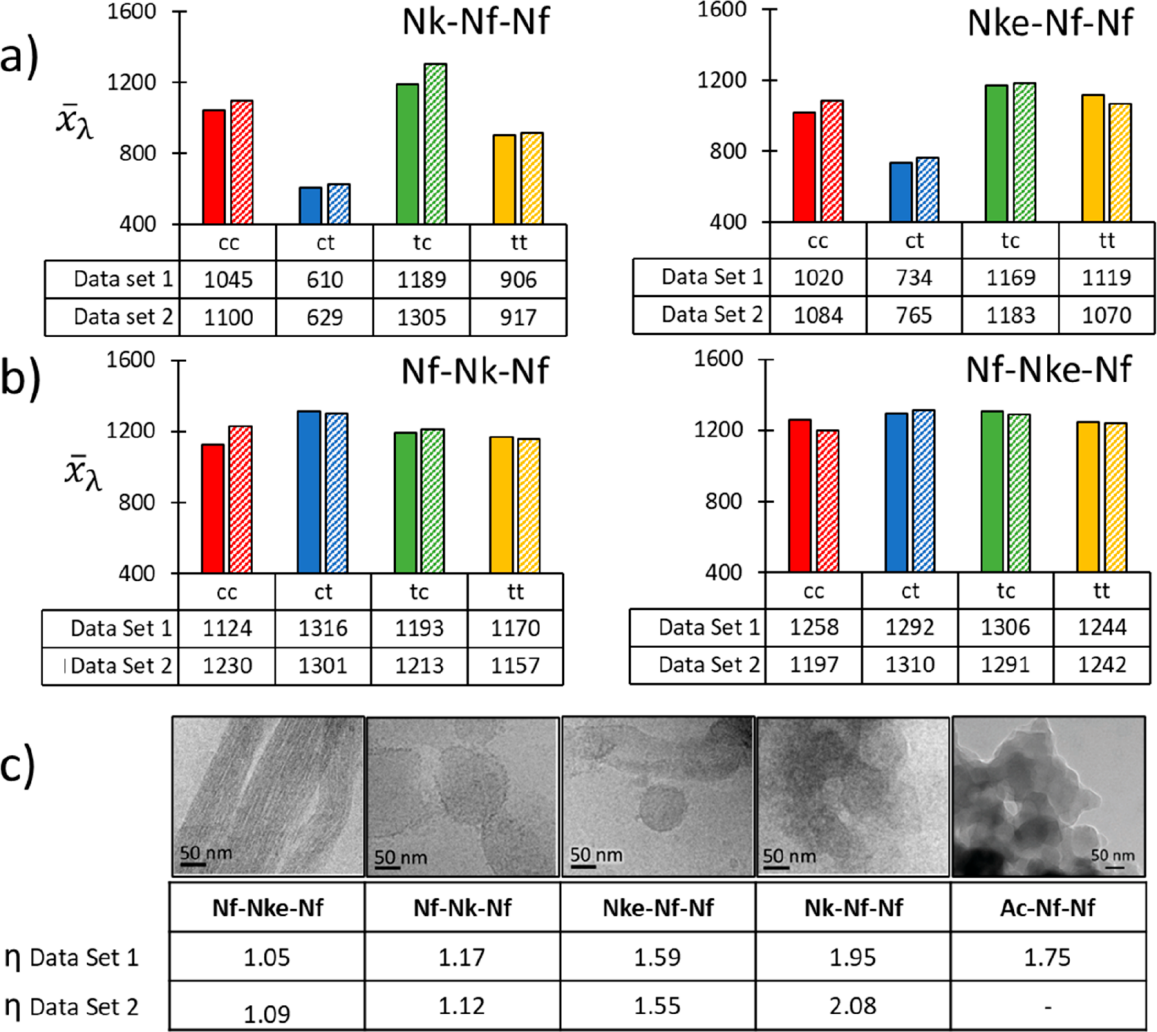
(a, b) *x̅*_λ_ values for each amide sequence are provided, from duplicate experiments and these show that for the XFF sequence the *ct* state appears to sample the λ domain less regularly (a), while for the FXF sequences this is visually more homogeneous (b). c) To enumerate this, the homogeneity parameter, η, was evaluated for the molecules in the dilute nonassembling MD systems, revealing a clear correlation between this parameter and the degree of order (morphological definition) of the nanoassemblies observed by Cryo-TEM/TEM images obtained in the original reports. Nf-Nke-Nf, Nf-Nk-Nf, Nke-Nf-Nf, and Nk-Nf-Nf in panel C reproduced with permission from ref (37). Ac-Nf-Nf panel C reproduced with permission from ref (36). Copyright 2019 RSC.

To characterize the apparent qualitative differences in λ sampling across amide states for the same molecule, a homogeneity metric was applied. First, by comparing *x̅*_λ_ values within a given sequence, it was found that sequences of the X-Nf-Nf pattern exhibit acute differences in λ sampling with respect to amide backbone conformation (Figures 6a). In contrast, for the Nf-X-Nf pattern, the *x̅*_λ_ values are more independent of the specific amide backbone conformation (Figure 6b). Furthermore, the *ct* amide conformation state (termed the *ct* motif) was found to sample the λ domain least for the XFF motif. This structure is illustrated for Nk-Nf-Nf in Figure 2.

Across the different sequences and across duplicate simulations, η was generally greater than 1.5 for XFF tripeptoids, while η is closer to 1.0 for the FXF sequences (Figure 6c). Considering the *x̅*_λmin_ average Δλ switching for these sequences (Figures 6a,b), we may estimate a hierarchy of sampling homogeneity as follows: Nf-Nke-Nf > Nf-Nk-Nf > Nke-Nf-Nf > Nk-Nf-Nf. We also note that the homogeneity across states follows the inverse of this relationship in the sequences studied. Indeed, this metric correlates well with the morphological structural “definition” apparent in the experimentally reported cyro-TEM and DLS measurements. Specifically, for Nf-Nke-Nf, a well-defined nanofiber is formed for η ∼ 1. In contrast, Nk-Nf-Nf with η ∼ 2.0 forms small ∼45 nm aggregates (Figure 6c).^37^ Thus, ignoring specific ρ vs λ regions and instead taking an ensemble view of the system for the moment, it is possible to correlate greater malleability in hydrophobic side chain sampling with the emergence of an ordered assembled structure.

On this basis, for XFF sequences, while the *ct* motif may give apparently optimal segregation of hydrophilic/hydrophobic side chains according to the conformationally pure FF dipeptide, this conformer exists in tripeptoids within a pool of other amide conformations that are structurally distinct. Because of the reduced ability to modify spatial arrangement for *ct* as implied by a high sequence η, poor tessellation within the hydrophobic domain would result, leading to growth-limited aggregates. Elsewhere tetrapeptide fibrillization has been shown to be rate limited by the formation of dimer precursors,^99^ which further suggests that the molecular arrangements within initial small aggregates will have critical implications on the subsequent assembly outcomes. Indeed, the entropic costs may be initially mitigated in the assembly process by apparently more dynamic sequences such as those with the Nf-X-Nf motif.

### 3.3 Absolute Molecular Entropy

To test our hypothesis that intrinsic dynamic properties of sequences can be informative of a predisposition for assembly, we evaluated the AME values for our peptoids of interest. To place these data in wider context, we also evaluated AME for all tripeptides corresponding to the XFF and FXF motifs that have been experimentally characterized.^31^ Among the tripeptoids, it is found that the Nk residue confers higher entropy than Nke (Table 1), which is expected because Nk contains additional methylene groups which increase flexibility. Moreover, the minimum enery conformer found for each sequence adopted the *cis*–*cis* conformation.

**Table 1:** AME Values of Tripeptoids **2**–**5**

name	molecular mass (g mol^–1^)	AME (cal mol^–1^ K^–1^)
Nk-Nf-Nf	441.6	217.4
Nke-Nf-Nf	413.5	204.7
Nf-Nk-Nf	441.6	222.8
Nf-Nke-Nf	413.5	203.9

**Figure 7:**
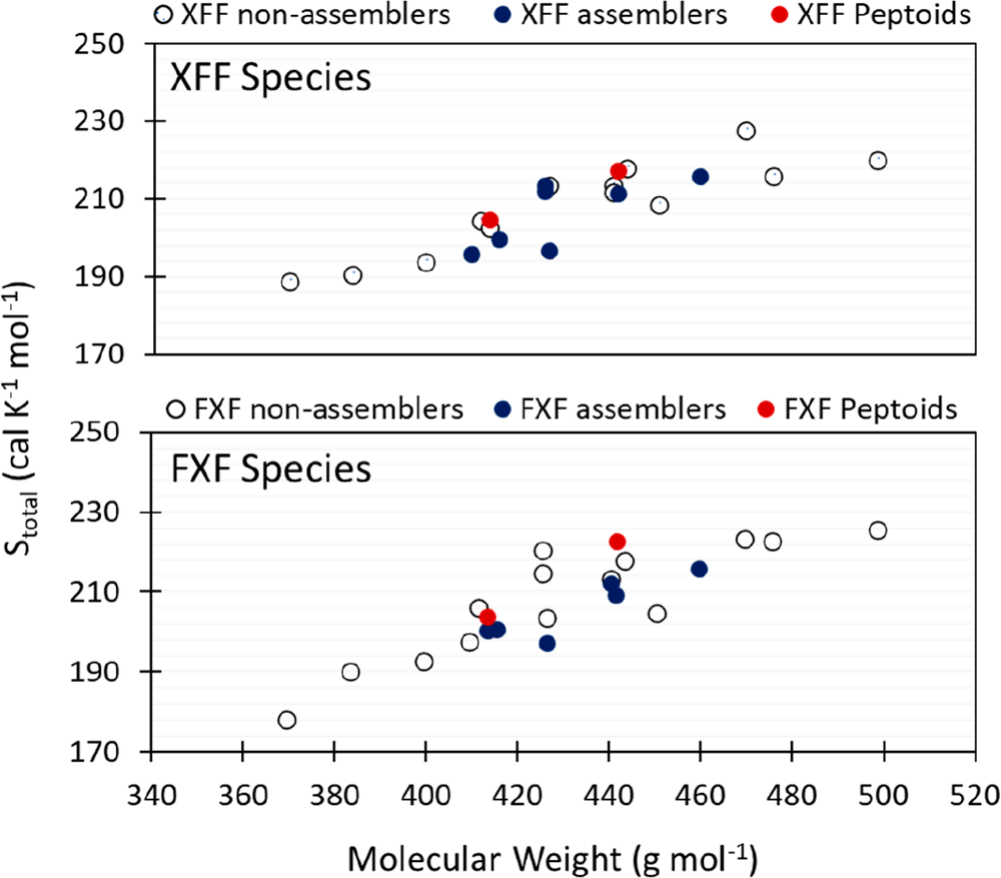
AME versus molecular weight for all canonical tripeptides with the XFF and FXF motif where X represents one of 20 canonical amino acids (all data available in SI Section 4). Overlaid are tripeptoids **2**–**5** that are relevant to this study (red). Nonassemblers are represented with a hollow circle and assemblers with a blue circle. All tripeptides and tripeptoids considered occupy a mass range of 360–500 g/mol, and AME values between 170 and 230 cal mol^–1^ K^–1^ were calculated.

However, we were surprised to find that AMEs for tripeptoids are on par with tripeptides of comparable molecular weight. This was counter to our expectation, given that peptoids can access many more ω and ψ/φ torsional microstates than their peptide counterparts.^67,77,100^Figure 7 shows that all tripeptoids and tripeptides roughly follow the same trend of increasing the AME with molecular weight.

Indeed, peptide assemblers and nonassemblers at similar molecular weights can exhibit similar AME values (Figure 7). More intriguing may actually be the fact that many nonassemblers are experimentally observed to assemble when converted to a heterochiral form (e.g., VFF,^95^ LFF,^101^ FIF,^102^ and FLF^102,103^), and we have included in our study a subset of heterochiral tripeptides (^d^VFF, ^d^LFF, F^d^LF, and F^d^IF) for which we also do not observe a significant entropy difference between the epimers (SI Section 4). As an example, we also measured the ρ vs λ surface for FLF and F^d^LF and observed they are qualitatively similar despite the difference in chirality (SI Section 3.2). Therefore, the potential emergence of assembly through the match or mismatch in residue chirality, which correspond to different noncovalent intermolecular interactions, may not be captured by AME. For example, it is notable that the assembling heterochiral tripeptide species all possess a mixture of aromatic and aliphatic hydrophobic side chains. Our analysis additionally draws attention to other nonassemblers such as NFF and FQF exhibiting AME within an apparent “assembly band” (415–460 g/mol, Figure 7). These nonassembling sequences are characterized by the absence of electrostatic interactions compared to carboxylate counterparts that do assemble, i.e., DFF and FDF/FEF, which highlights the importance of noncovalent interactions over intrinsic entropy.

### 3.4 Gas Phase Dynamics: ANI-1ccx MD

Given that AME does not indicate a clear distinction between assemblers and nonassemblers, we turned our attention to external factors, specifically the major role the solvent environment plays, in considering the lower apparent ability of X-Nf-Nf tripeptoids to assemble with long-range order. Toward this end, we used the ANI-1ccx potential to perform single molecule gas-phase simulations, which leverages near chemical accuracy with a reduced cost versus methods such as *ab initio* DFT.^92^

It was found in many tripeptoid cases that the ρ vs λ surfaces are in qualitative agreement with those obtained in the aqueous phase using the force field parametrized in this study (e.g., Nf-Nk-Nf (*tc*), Figure S61). For the dipeptoid Ac-Nf-Nf, qualitative agreement is found in all cases except for *cc* (Figure S62). The reason for the discrepancy is not immediately obvious.

**Figure 8:**
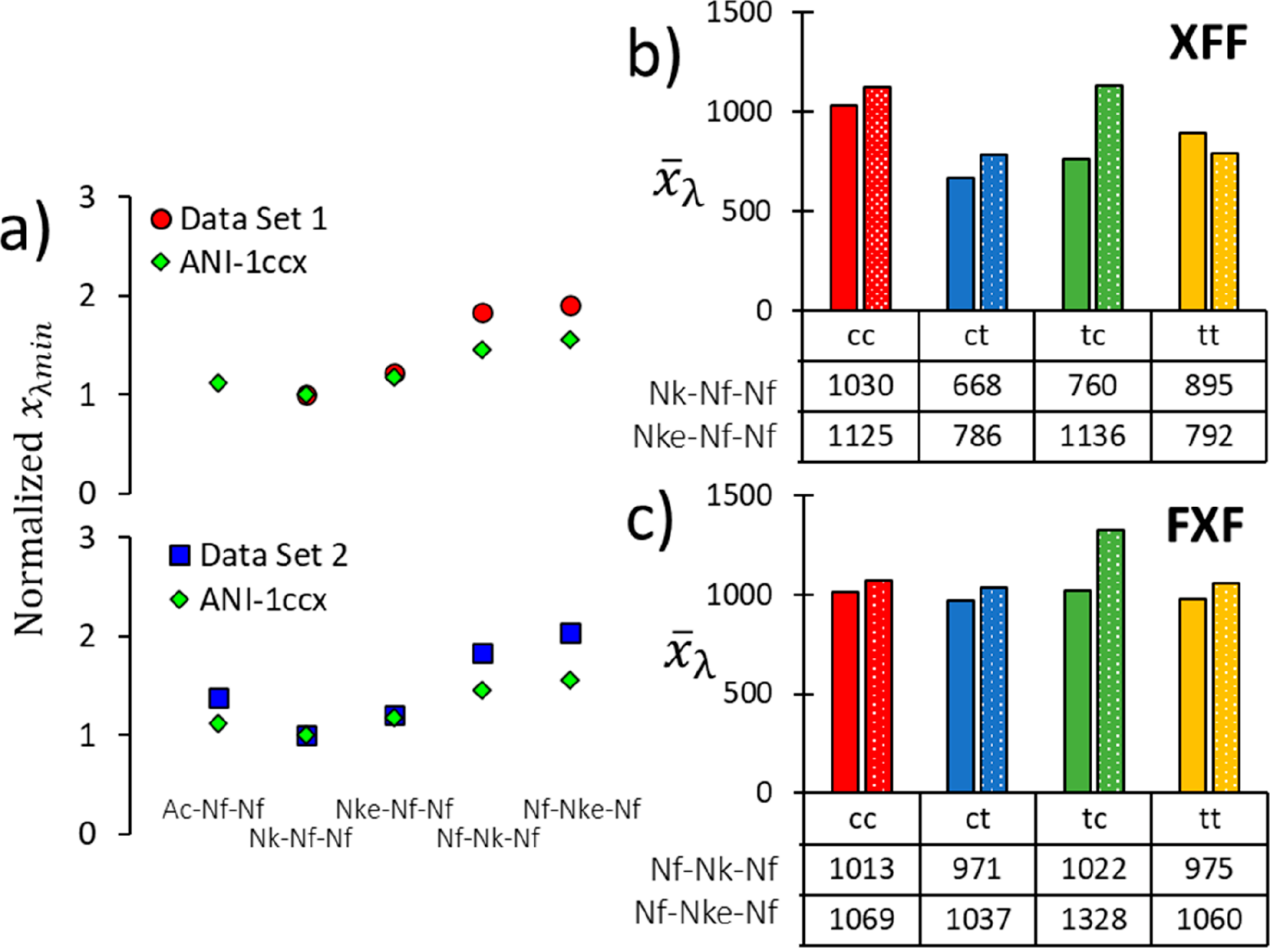
(a) Comparison of normalized *x̅*_λmin_ values between aqueous and gas-phase simulations of all peptoids of interest, showing the same general trend and indicating that solvent effects the dynamics of the Nf-X-Nf sequences more than the X-Nf-Nf. (b) Gas phase values of *x̅*_λ_ for tripeptoids Nk-Nf-Nf and Nke-Nf-Nf showing that the *ct* state remains the state with least λ sampling. (c) Gas phase values of *x̅*_λ_ and Nf-Nk-Nf and Nf-Nke-Nf indicating greater consistency between states as found in aqueous simulations, though more so for the former than the latter.

Nonetheless, by comparing *x̅*_λ_ values, it is apparent that a qualitative agreement between the sampling properties of a given sequence across amide states are consistent between aqueous and gas phase environments (Figures 6a,b and 8b,c). Thus, this is an intrinsic property of that sequence, e.g., X-Nf-Nf or Nf-X-Nf. Within these individual motifs, when η is evaluated, it was revealed that Nf-Nk-Nf is more homogeneous with respect to its backbone conformation than Nf-Nke-Nf, whereas the reverse is true in the aqueous context (Table S43). In contrast, for X-Nf-Nf tripeptoids, the trend in η values is consistent with the aqueous phase (e.g., η_Nk-Nf-Nf_ > η_Nke-Nf-Nf_), suggesting that the intramolecular dynamics of this motif are less sensitive to solvent effects.

Another indicator of this intramolecular sampling persistence is found when comparing normalized *x̅*_λmin_ values between the aqueous and gas phase. Specifically, for the X-Nf-Nf tripeptoids the normalized *x̅*_λmin_ values are essentially conserved across gas and aqueous phases conditions (Figure 8a). In contrast for Nf-X-Nf tripeptoids, it is found that the normalized *x̅*_λmin_ values are lower in the gas phase than their aqueous phase counterparts, suggesting that solvent drives sampling in this sequence to an extent. This effect may also account for the difference in η between the phases for Nf-X-Nf.

An additional corollary is that ρ in the gas phase is “capped” at 1.2–1.4, whereas in aqueous simulations sampling of ρ up to ∼1.8 is regularly observed for λ = −30° to 30° (Figures S45 and S46). When ρ > 1.0, then *R*_para_ < *R*_ipso_, and this corresponds to a burial of a gap between aromatic rings (Figure S63). The hydrophobic effect, driven by TIP3P solvation, will promote aromatic ring organization in this manner. Such an outcome will not be realized for an X-Nf-Nf type case, where aromatic rings are adjacent within the chain, precluding a monomer spaced gap emerging. On this basis, homogeneity of side chain dynamics is a molecularly intrinsic property. The influence of solvent is not uniform, however, with X-Nf-Nf sequences being less susceptible than Nf-X-Nf sequences.

## 4 Conclusion

Peptoids are achiral in their backbone and may occupy both *cis* and *trans* ω torsions. They furthermore lack intra- and interbackbone hydrogen bonding. Despite these modifications, dipeptoids and tripeptoids can still form well-defined self-assembled nanostructures. The incorporation of phenyl side chains in short assembler design is well established in analogous short peptide assemblers. For this reason, we developed a series of descriptors (ρ, λ, and η) that relate to the dynamics of aromatic rings to assess how these change with amide backbone conformation and to evaluate if this contains information about a molecule’s predisposition for assembly. By way of validation, we applied these metrics to known di- and tripeptide assemblers.

Our analysis of ρ and λ revealed that the amide backbone conformation has a significant impact on the sampling of aromatic side chain configurations for the same peptoid molecule. Additionally, it was found that the sequence pattern, FXF or XFF, where X = nonaromatic residue, also affects this molecular property for both short peptoids *and* peptides. We then evaluated how sampling of the aromatic ring torsion, λ, varies across amide states for the same molecule and found that a homogeneity of aromatic configuration sampling, i.e., η ∼ 1.0, correlates well with the formation of well-defined assembled structures. Therefore, perhaps counterintuitively, malleable aromatic configurations lend molecules an apparent predisposition to form extended structures.

Based on the observation that conformational exploration appears to be central to this difference in peptoid assembly behavior, we compared the AME for our tripeptoids to all canonical tripeptides with the XFF and FXF motif. Interestingly, AME is not correlated to assembly differences, and a comparative analysis of heterochiral tripeptide assemblers also found limited differences in AME, suggesting that intermolecular interactions may instead be driving the differences in exploration of the ρ–λ conformational space. To assess this, we performed gas-phase MD simulations with the ANI-1ccx potential and found generally qualitative agreement in ρ vs λ sampling between the gas phase and classical aqueous phase MD simulations. However, it was revealed that the sampling of FXF and the corresponding homogeneity, η, is modified to an extent by the presence of solvent, suggesting that interaction with water drives sampling in such sequences. In this effort, we have also shown that our MD force field with reparametrized partial charges can identify the relevant minima, as confirmed by additional force field characterization work and agreement of sampling with ANI-1ccx MD.

To conclude, we provide a fresh perspective on the peptide/peptoid assembly propensity by focusing on shared aromatic features as opposed to drivers such as backbone hydrogen bonding, which is not accessible for peptoids (except at chain ends). We identify that the predisposition for assembly of short peptoids is dependent on monomer patterning and the degree to which side chain dynamics are independent of specific backbone amide conformation. Additionally, compelling parallels in ρ–λ conformational space sampling exist between corresponding peptides and peptoids, at least for dimer and trimer sequences, which highlights the contribution of phenyl side chain stacking interactions in driving both peptide and peptoid assembly. We therefore believe this enhanced understanding can inform judicious sequence design for short peptoid assemblers and provides metrics which enable *in silico* property evaluation of candidate sequences.

## Data Availability

All data underpinning this publication are openly available from the University of Strathclyde KnowledgeBase at 10.15129/26c722b8-c6e2-49b2-9c9a-e9091bc0ddf2.

## Supporting Information Available

Figures S1–S72 and Tables S1–S41 (PDF)
Force field parameters (TXT)
All topology information (TXT)
