# Supplementary material for: Minimal Peptoid Dynamics Inform Self-Assembly Propensity
Source: J Phys Chem B. 2023 Dec 1;127(49):10601–14. doi: 10.1021/acs.jpcb.3c03725 (PMC10726364; doi:10.1021/acs.jpcb.3c03725)
Supplement: Supplementary file 1 — jp3c03725_si_003.pdf [file jp3c03725_si_003.pdf]

Minimal Peptoid dynamics inform self-assembly propensity

Hamish W A Swanson, King Hang Aaron Lau and [*] *Tell Tuttle*[*]

Department of Pure and Applied Chemistry, University of Strathclyde, 295 Cathedral Street, Glasgow G1 1XL, UK.

1. Partial Charge Parameterization

1.1 Methods

1.1.1 Requirements

In this work the peptoid backbone is described using the bonded parameters reported Weiser *et al.*[1] are used. However, the collation and refinement of a wide variety of parameters for peptoid simulations was the goal of this parameterization. We chose to do this for 31 relevant monomers from the literature (Figures S1) and used the CGenFF initial guess utility[2] to identify and generate bonded terms for this purpose, where relevant refinements of torsions were also done (Section S2). Additionally, we found it necessary to generate partial charge sets to correctly match the electrostatic contributions of a given residue to the force field Hamiltonian. In the CHARMM force field the electrostatic contribution is evaluated using Eq. 1.1.

$$\sum_{Coulombic} \frac{q_i q_j}{\epsilon_1 r_{ij}} \tag{1.1}$$

Where values q_i and q_j, correspond to the partial charges of atoms *i* and *j*, $\epsilon_1$is the dielectric constant (set to 1.0 in this work) and r_{ij} is the distance between charged *i* and *j*.

For polar molecules, these partial charges are fit such that the energies of multiple interactions between the molecule of interest and a TIP3P water molecule can be reproduced within 0.2

kcal/mol of these target energies. Furthermore, for residues in which optimizing stable hydrogen bonding complex is challenging (i.e., highly hydrophobic molecules) the reproduction of the HF/6-31G(d) dipole moment orientation is used to validate the partial charge set. Ideally the deviation from the orientation should be less than 20° and the magnitudes are ideally overestimated by a factor of 1.2 to 1.5 to reflect bulk-phase values.[3]

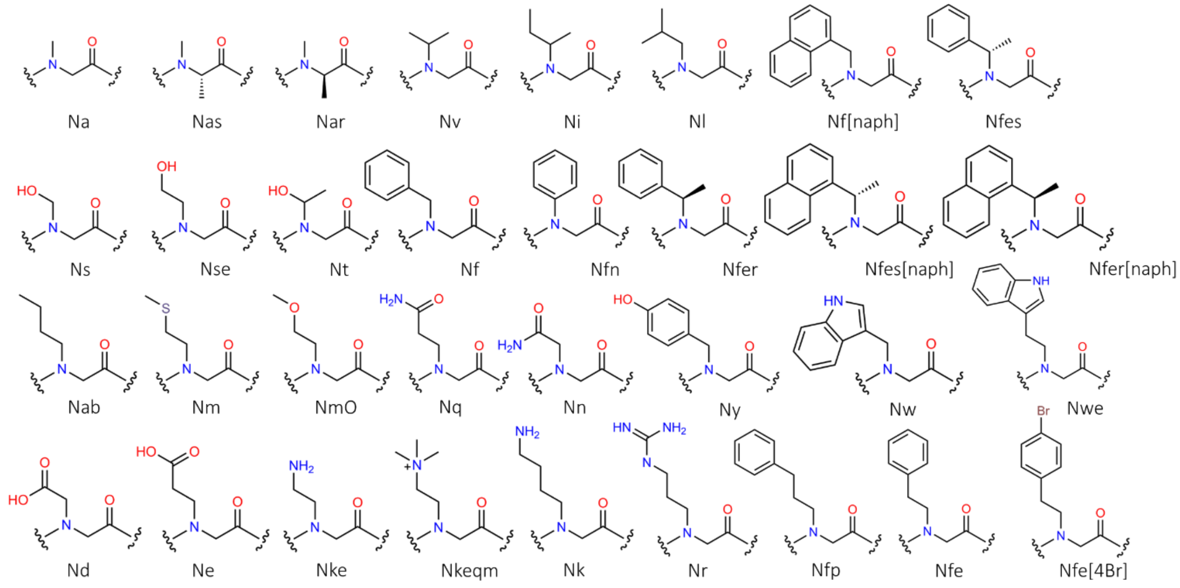

Figure S1. Peptoid monomers parameterized in this work. These capture a broad base of chemistries and are relevant within literature. Note that our naming convention can be applied to all monomers.

1.1.2 Methodology

Target Data

A molecule of interest was optimized at the MP2 level of theory with the 6-31G(d) basis (MP2/6-31+G(d) in the case of anions). Water interaction complexes are obtained in which all degrees of freedom are fixed except that of the water molecule position between the molecule and water in the TIP3P geometry; these are optimized at the HF/6-31G(d) level. The energy of this complex was then evaluated using NAMD for a given partial charge set. Note that the water interaction distance is reduced by 0.2 Å and the QM interaction energy is multiplied by a factor of 1.16 to scale for the bulk phase, this is necessary to correct for the *in vacuo* nature of these calculations.[4] The reduction in interaction distance is made because the Hartree-Fock model overestimates minimum interaction distances because dispersion contributions are absent from this model, as are many body effects.[4] Importantly, for charged compounds scaling is not necessary, as bulk property reproduction is satisfactory without scaling. No correction was applied for basis set-superposition errors at the HF/6-31G(d) level; this level of theory is used for consistency with other CHARMM additive force fields and its low associated computational cost. The interaction energy is thus represented by Eq 1.2, where the potential energy contribution of TIP3P water is set to zero within CHARMM.

$$E_{Hydrogen-Bond} = E_{complex} - E_{moleule-SPE} - E_{TIP3P-SPE} \qquad (1.2)$$

All relevant QM data was obtained using Gaussian16.[5] Atom types and new parameters for novel monomers were determined using the automated parameter generation tool[6, 7] following on from the work of Weiser *et al.* we used their previously defined NTOID atom type, which uses the Lennard-Jones parameters of a protein-like NG2S1 CGenFF nitrogen.[1] Partial charge auto generation using Force Field Tool Kit (ffTK)[8] was not done as a more in-depth parameterizations was desired to produce partial charges consistent for a class of molecules, as opposed to on an individual monomer basis. Similarly, it was also decided to not use CHARMM fixed values in cases were carbon and hydrogen atoms were in the proximity of electronegative atoms (*e.g.*, aliphatic hydrogen atoms possessing charges of +0.09).[3] We were motivated to do this to maximize the

chemical detail that might be discerned from this parameterizations scheme and to avoid the loss of potentially important detail through fitting.

Generating Partial Charge Sets

With this information in hand, it was then necessary to generate hypothetical partial charge sets and evaluate how well they reproduced the electronic topology of the residues of interest. To do this the python module, itertools,[9] to generate partial charge sets that summed to the net charge of the residue of interest. The itertools python module was used initially to generate combinations, Eq. 1.3, which satisfied this requirement from a range of input values. A grid size of 0.05e was used, typically within a range of -0.5e to 0.5e per atom, though the grid size was adjusted as required and in many cases subsequent tuning of partial charge sets was done by hand once a good estimate was found.

$$C(n,k) = \frac{n!}{(n-k)!k!} \quad (1.3)$$

$$P(n,k) = \frac{n!}{(n-k)!} \quad (1.4)$$

where n is the partial charge range (*i.e.*, input array for combinations) and k the size of the partial charge array, namely the number of atoms in the residue.

These combination sets were then used as 'seeds' from which permutations could be generated. Combinations, Eq. 1.3, are non-sequence dependent arrays, for example the combination [A, B, C] is equivalent to [B, A, C]. In contrast, permutations Eq. 1.4, are sequence dependent arrays. As such the two examples given are distinct permutations. Consequently, there are grossly more permutations for any given array than there are combinations. Therefore, for reasons of computational efficiency the generation of combinations that satisfy charge requirements first greatly cuts down the number of hypothetical partial charge sets.

Each permutation generated was then mapped onto an array of atoms for which the partial charge set was being generated. Upper and lower limits of possible partial charge values were then used to cut down this array-space, such that only permutations that made chemical sense

were permitted to pass through to the next stage of calculation. These values were determined both by comparison to related structures *e.g.*, CHARMM22 amino acids, as well as by the application of chemical knowledge. For example, carbonyl carbons will not have a negative partial charge. This discriminatory process vastly cut down the search space.

Evaluating Partial Charge Sets

The partial charge sets were applied to the molecular structure and the dipole moment magnitude. Only those which satisfactorily overestimated the HF/6-31G(d) dipole moment magnitude by 1.2 – 1.4 passed this test. Where no partial charge sets gave estimations within this range, this step was omitted, and partial charges were validated against energy before refinement for reproduction of dipole moment. These two steps taken together cut the number of permutations from several million hypothetical sets to several hundred sets for further testing.

1.1.3 Peptoid Backbone Treatment

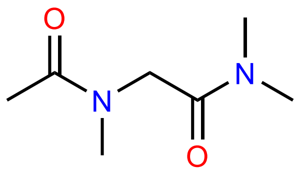

1

Figure S2. N-acetyl-sarcosine-N’, N’-dimethylamide, disarcosine peptoid.

To reduce the number of calculations required for residue parametrization it was decided to first focus on the peptoid backbone partial charges, so that a transferable representation could be produced which would then be applied in all monomers. A disarcosine peptoid (Figure S2) was used to perform this parameterization. All subsequent monomer parameterization used this subunit or variations of it. The N-terminus in this species was acylated and the C-terminus is a dimethylated nitrogen. Partial charge values for these protecting groups were taken from the work of Weiser *at al.*[1] and did not differ significantly from those of the CHARMM22 force field.

An adequate partial charge set was produced for peptoid **1** which correctly reproduced the dipole moment and orientation. The set that was selected had the smallest mean absolute error (MAE) across multiple water interactions, as calculated using Eq. 1.5,

$$MAE(\hat{\theta}) = \frac{1}{n}\sum_{i=0}^{n}\left|\hat{\theta}_i - \theta_i\right| \quad (1.5)$$

where $\hat{\theta}_i$ is the QM energy at the HF/6-31G(d) level, θ_i is the CGenFF energy for the same interaction using the generated partial charge set; this is done for each interaction and an average is taken.

Once an adequate partial charge set was produced; with an appropriate MAE, which correctly reproduced the dipole moment magnitude and orientation, it was necessary to adapt these charges for non-primary N-C_β side chains. To do this, partial charge sets were generated which summed to the net charge necessary to render the backbone neutral. The carbonyl unit had fixed partial charges, while C_α was allowed to only vary slightly and N and the C_β unit charges could vary more widely. This assumed that the greatest variation between side chain electronic topology would be at the tertiary amide nitrogen. These hypothetical charges were tested against dipole moment for secondary N-C_β side chains aliphatic and aromatic side chains. This same procedure was completed for tertiary N-C_β side chains and in this case wider variation in the C_α unit partial charge was permitted. The partial charge sets were then applied throughout the parameterization protocol.

To validate quality of dipole moments determined for partial charge sets the dipole moment magnitude was calculated as the vector magnitude. While the angle of deviation between the calculated vector and that at the HF/6-31G(d) level was calculated using Eq. 1.6.

$$cos\, \theta = \frac{\vec{u} \cdot \vec{v}}{|u| \cdot |v|} \quad (1.6)$$

1.2 Results

1.2.1 Disarcosine Backbone

Using the method outlined in Section 1. a partial charge set was generated for the disarcosine peptoid, denoted Na, for three optimized water interactions. Each TIP3P water interaction was reproduced within ±0.2 kcal/mol, with an MAE within this range. The energy results are given in Table S1. The partial charge set correctly reproduced both the dipole moment orientation as required. Similarly, the magnitude of the dipole moment was found to be 5.32 D corresponding to an overestimation of the QM value, 3.81 D, by a factor of 1.4. These data indicate that the partial charge set is an appropriate representation of the electronic topology of the disarcosine molecule.

Table S1 – Interaction energies (kcal mol^{-1}) for disarcosine peptoid using calculated partial charge set.

Complex	ΔE (HF)	ΔE (CGenFF)	ΔΔE
**Interaction-1**	-8.18	-8.01	-0.18
**Interaction-2**	-4.90	-4.76	-0.15
**Interaction-3**	-4.90	-5.05	0.14
		MAE	0.16

The partial charges and atom types used in the work of Mirijanian *et al.*[10] and Weiser *et al.*[1] were applied to this test system and the same energy calculations were performed. This was done to confirm the necessity of partial charge parameterization. In the case of the disarcosine model presented for the MFTOID force field, it was found that the water interaction energies were underestimated, Table S2. Each interaction was out-with the acceptable CGenFF limits, as was the resultant MAE value. Despite this the resultant dipole moment was overestimated by a factor of 1.23 as required. In the MFTOID parameterization procedure the reproduction of water

interaction energies was not included in partial charge validation. Instead, bulk properties and dipole moment were used.

Table S2 – Interaction energies (kcal mol^{-1}) for disarcosine peptoid using MFTOID partial charge set.

Complex	ΔE (HF)	ΔE (CGenFF)	ΔΔE
**Interaction-1**	-8.18	-7.64	-0.54
**Interaction-2**	-4.90	-3.27	-1.63
**Interaction-3**	-4.90	-3.64	-1.27
		MAE	1.15

The origin of the partial charge set reported by Weiser *et al.* for disarcosine is not explicitly stated. These charges were the least successful at reproducing the electronic topology of disarcosine, with each interaction energy being underestimated, giving rise to the largest MAE for these partial charge sets, Table S3. An overestimation of the dipole moment magnitude by a factor of 1.18 was obtained, which is slightly lower than that required for the CGenFF. The ambiguity surrounding the source of these partial charges makes it difficult to define specifically what is the origin of this underestimation.

Table S3 – Interaction energies (kcal mol^{-1}) for disarcosine peptoid using CGenFF-WS peptoid model.

Complex	ΔE (HF)	ΔE (CGenFF)	ΔΔE
**Interaction-1**	-8.18	-7.33	-0.85
**Interaction-2**	-4.90	-3.17	-1.73
**Interaction-3**	-4.90	-3.61	-1.30
		MAE	1.29

It is evident through water-complex interaction energies that current CHARMM based peptoid force fields underestimate this non-bonded energy contribution and highlights that it is not valid to transfer partial charge parameters for other side chains without adequate parameterization.

In Table S4 the partial charges for each CHARMM representation of the disarcosine residue, as well as the Merz-Kollman (MK) charges at the MP2/6-31G(d) level of theory is shown. We considered a deviation of ± 0.1e from the partial charges for the other peptoid force fields to be significant, this value corresponds to two times the grid size used in generating the partial charge sets. For carbonyl and peptoid nitrogen the deviation between partial charges for the calculated set and the other CHARMM models is not considered to be significant, Table S4. Despite this the close similarity between the CHARMM models gives us confidence in the calculated value for these units in the peptoid backbone.

Table S4 – Partial charges for carbonyl system and peptoid nitrogen; calculated, in literature forcefields and at the HF/6-31G(d) level.

	**C**	**O**	**N**
**Calculated**	0.580	-0.580	-0.350
**MFTOID**	0.510	-0.510	-0.420
**Wesier *et al.***	0.530	-0.530	-0.350
**HF/6-31G***	0.544	-0.598	-0.081

At a first glance, on a per atom basis, the calculated partial charge values for both the C_α unit and the C_β unit are significantly different from other peptoid models, Table S5. However, when the net charges are considered a reasonable agreement with previous findings exists. For the C_α unit, the difference from the MFTOID is 0.08e, the previous CGenFF model by 0.042e and the MK charges by 0.099e. An even better agreement between the calculated values and these data is found for the C_β unit, with net charges differing by 0.01e from the MFTOID, 0.037e for the CGenFF-WS model and 0.183 from the MK charges. These agreements between the partial charge subunits of the peptoid backbone indicate clear similarities between the calculated partial charge values, those from of previous peptoid models

Table S5 – Partial charges for C_α and C_β units; calculated, in literature forcefields and at the HF/6-31G(d) level.

	**C_α**	**$H_\alpha1$**	**$H_\alpha2$**	$\sum q$	**C_β**	**$H_\beta1$**	**$H_\beta2$**	**$H_\beta3$**	$\sum q$
**Calculated**	-0.470	0.300	0.300	0.130	-0.251	0.157	0.157	0.157	0.220
**MFTOID**	0.030	0.090	0.090	0.210	-0.060	0.090	0.090	0.090	0.210
**Wesier *et al.***	-0.008	0.090	0.090	0.172	-0.087	0.090	0.090	0.090	0.183
**HF/6-31G(d)**	-0.200	0.130	0.101	0.031	-0.368	0.160	0.120	0.129	0.041

Considering the consistency between partial charge values between the peptide nitrogen, carbonyl and C_β units, the calculated values are in general agreement with models that have come before. The major difference being the now correct interaction energy reproduction. The most significant difference between previous CHARMM models and this partial charge set exists for the net charge of the C_α unit. This is therefore likely to be the sub-unit of the backbone which most required partial charge parameterization for improved interaction-energy reproduction.

Table S6. Peptoid partial charge sets for primary, secondary, and tertiary C_β unit peptoid backbones.

**N-C_β**	**C**	**O**	**N**	**C_α**	**$H_\alpha1$**	**$H_\alpha2$**	**C_β**	**$H_\beta1$**	**$H_\beta2$**	**$H_\beta3$**
**1°**	0.580	-0.580	-0.350	-0.470	0.300	0.300	-0.251	0.157	0.157	0.157
**2° Type 1**	0.580	-0.580	-0.450	-0.500	0.300	0.300	-0.150	0.250	0.250	-
**2° Type 2**	0.580	-0.580	-0.400	-0.500	0.300	0.300	-0.200	0.250	0.250	-
**2° Type 3**	0.580	-0.580	-0.450	-0.350	0.300	0.300	-0.220	0.210	0.210	-
**3° Type 1**	0.580	-0.580	-0.300	-0.350	0.200	0.200	0.250	0.000	-	-
**3° Type 2**	0.580	-0.580	-0.150	-0.250	0.175	0.175	0.050	0.000	-	-
**4 Type 1**	0.580	-0.580	-0.05	-0.300	0.175	0.175	0.000	-	-	-

The partial charge sets for secondary and tertiary backbones, generated using the procedure outlined in Section 1.1, are presented in Table S6. These were applied to the peptoid monomers

parameterized in the following sections. Additional variations of the backbone partial charge set were generated for secondary, tertiary, and quaternary peptoid sidechain types. These differ most significantly in the C_β unit and to a lesser extent the C_α unit for both substitution types. The use of these is validated by the agreement between CGenFF dipole moment magnitude and orientation estimates and those at the HF/6-31G(d) level found in Section 1.2.2 for non-polar sidechains. These differ most significantly in the C_β unit and to a lesser extent the C_α unit.

1.2.2 Non-Polar Side Chains

Partial charges for the non-polar side chains parameterized in this work were generated from two sources. The backbone partial charge set was selected from those defined in Section 1.2.1, for secondary side chains both types were tried and those with the best dipole moment values were adopted. For side chain partial charges, at a distance from heteroatoms, fixed CHARMM values were adopted for aliphatic and aromatic groups; typically, these were taken directly from the closest related CHARMM22 amino acid or from the CGenFF topology files for closely related side chains (*e.g.,* naphthalene or halogenated benzene). Generally, these satisfy the CHARMM requirement for dipole moment overestimation of the *in-vacuo* HF/6-31G(d) dipole moment by a factor of 1.2 – 1.5 of dipole moment overestimation (Figure S3a). This is achieved for almost all the 14 side chain types. Nab, which contains an *n*-butyl side chain attached to the peptoid amide nitrogen (Figure S1). The dipole moment is underestimated at 1.74 D versus a QM value of 3.27 D. It is thought that this is because the fixed peptoid backbone types were selected for side chains with greater side chain bulk (*e.g.,* aromatic rings in phenylalanine analogues) or branched aliphatic units (*e.g.,* leucine, isoleucine, and valine analogues).

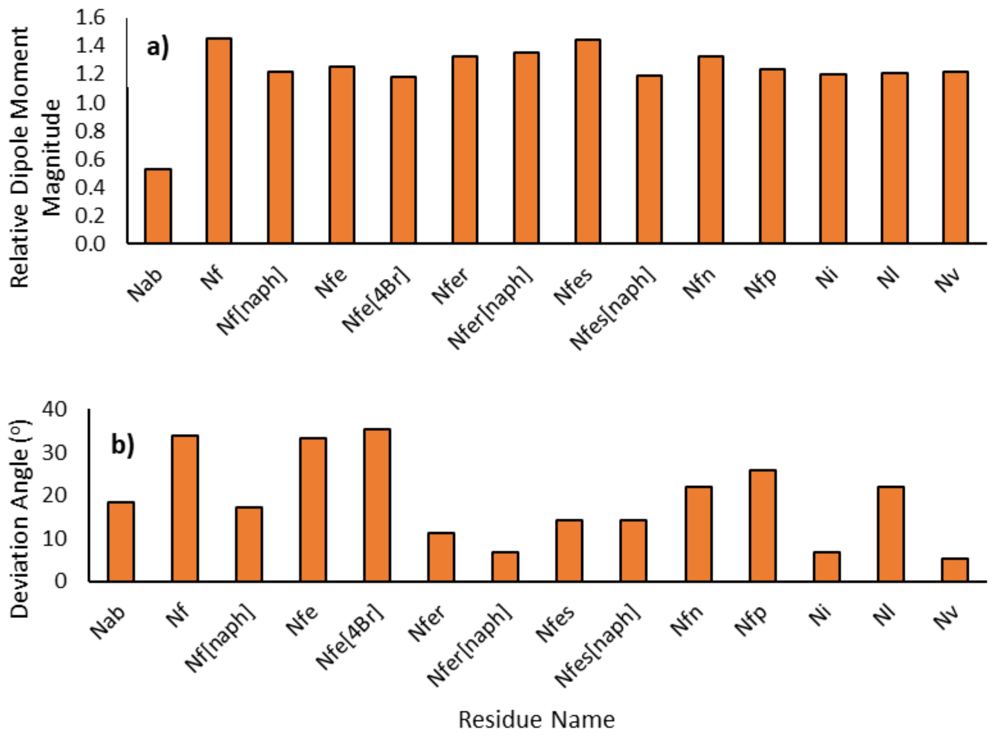

Figure S3. Relative dipole moment magnitude per non-polar side chain residue. Ideal CGenFF overestimation range, by a factor by a factor of 1.2 to 1.5 a), Angle of deviation between dipole moment vector for calculated and HF/6-31G(d) partial charges for non-polar residues b).

The average deviation between the dipole moment vector using the calculated charges the HF/6-31G(d) dipole moment was found to be 19° ± 10° for the non-polar side chains (Figure S3b). Considering the fixed nature of the backbone partial charge and the approximation involved in adopting fixed CHARMM partial charges these deviations are thought to be reasonable. It can be concluded that the adoption of fixed backbone partial charges is valid for non-polar side chains. For most residues, the dipole moment is overestimated as required and the deviation angles from the dipole moment orientation at the HF/6-31G(d) level are also reasonable. As such the fixed backbones were applied to polar side chains in Section 1.2.3.

1.2.3 Polar Side Chains

The MAE values for water interactions, for polar side chain partial charge sets are generally good (Figure S4a). Of a total of 11 side chains, 9 are within the range of ± 0.2 kcal/mol as required by the CGenFF across all QM hydrogen bonding interactions. It is evident that both Nas and Nq do

not fit these limits by a significant margin. This is a consequence of the large variation between optimized interaction complexes obtained for these structures. In the case of Nq this is manifest in the primary amide, by comparing the *cis* (HD1) and *trans* (HD2) proton partial charges for each interaction, in Table S7, the source of this deviation is clear.

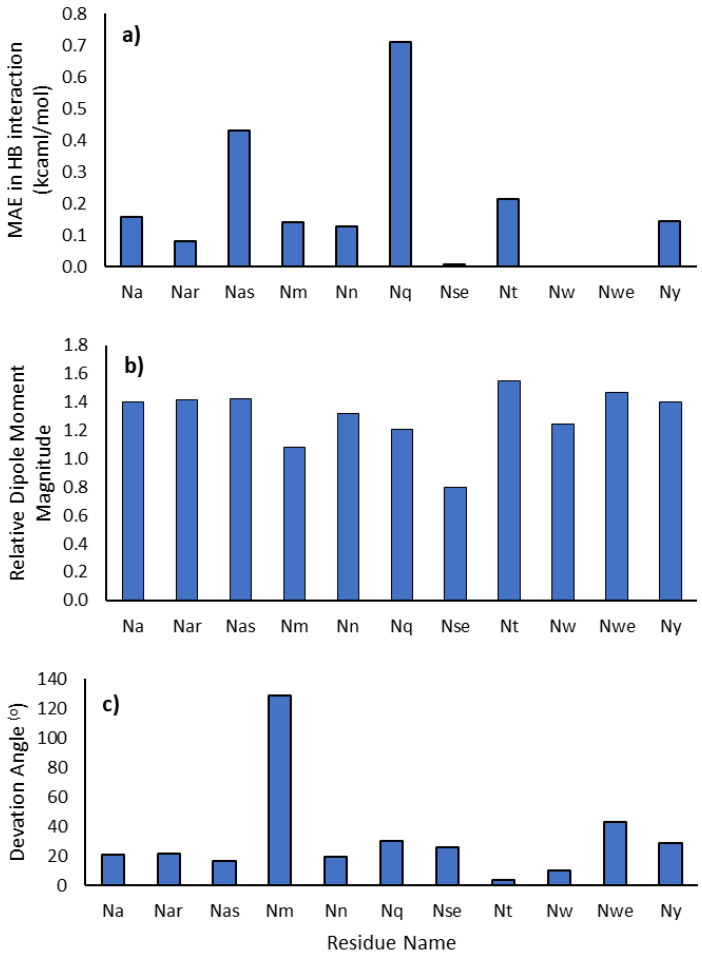

Figure S4. MAE between water interaction energy via calculated partial charges and HF/6-31G(d) optimized structures, for multiple hydrogen bonding complexes of polar peptoid residues a), Relative dipole moment magnitude per polar side chain residue. Ideal CGenFF overestimation range, by a factor by a factor of 1.2 to 1.5 b), Angle of deviation between dipole moment vector for calculated and HF/6-31G(d) partial charges for polar residues c).

The partial charge distribution across the amide -NH_2 unit is asymmetric, with a larger density of charge on the proton *trans* to the carbonyl nitrogen. One optimized interaction lies within the *cis* plane and another optimized towards the *trans* proton; these redistribute the charge of the amide protons in a manner which is somewhat symmetrically opposite depending on which

complex is considered. Therefore, the first complex energy is reproduced well and the second poorly (Table S12). Following the precedent of the CHARMM22 force field glutamine residue asymmetry exists for the fixed partial charges and we selected a partial charge set which follows this choice in charge assignment. This case is representative of the limitations of a fixed partial charge set representation of electron density as both interactions occur within a solvated system, though one is described better electrostatically. Note this was not evident for Nn (asparagine peptoid derivative) as the water molecules were optimized in a different arrangement which was less polarized as that for Nq.

Table S7 – Partial charges for Nq side chain primary amide; as well MK partial charges and the CHARMM22 glutamine fixed charges.

	**C**	**O**	**N**	**HD1(cis)**	**HD2(trans)**
**HF/6-31G(d)**	0.900	-0.639	-1.111	0.460	0.448
**Calculated**	0.650	-0.650	-0.680	0.350	0.330
**CHARMM22 (Gln)**	0.550	-0.550	-0.620	0.320	0.300

The significant MAE value for Nas (Figure S4a) is also observed as a consequence of the challenge associated with super-imposing partial charges for multiple interactions onto one structure. Further complexity was added to this case by the decision to apply the partial charge set from enantiomer Nar to the Nas system, on the assumption that partial charge is conserved between enantiomers. Such an approach proved successful for the non-polar enantiomers Nfes/Nfer and Nfes[naph]/Nfer[naph] (Figure 3a) and so it was adopted in this case also.

The dipole moment overestimation, as stipulated by the CGenFF protocol is generally satisfied for the polar side chains (Figure S4b). Notably Nm underestimates the magnitude of the dipole moment and like Nab is an extended linear side chain. This further supports the idea that the selected side chain types do not satisfactorily overestimate dipole moment magnitudes. Significant underestimation of the dipole moment is also seen for Nse, which is an ethyl derivative of the peptoid analogue of the amino acid serine. Though only three atoms long as opposed to

four for Nab and Nm, the effect of poor extended side chain reproduction also appears to be expressed in Nse.

For the monomer Nm significant deviations between the angle of the calculated dipole moment and that at the HF/6-31G(d) level exist (Figure S4c). It is important to note however that Nab has a below average vector deviation angle which indicates that poor dipole moment magnitude and vector reproduction are not coupled properties. The deviation angle is also poor for Ns and Nwe, though the basis for this is not clear. It is also important to note that for many residues the angle of deviation between the calculated and QM vectors are reasonable.

Table S8 – Nar interaction energies (kcal mol^{-1}).

Complex	ΔE (HF)	ΔE (CGenFF)	ΔΔE
**Interaction-1**	-8.20	-8.22	0.02
**Interaction-2**	-4.61	-4.56	-0.05
**Interaction-3**	-8.13	-8.31	0.17
		MAE	0.08

Table S9 – Nas interaction energies (kcal mol^{-1}).

Complex	ΔE (HF)	ΔE (CGenFF)	ΔΔE
**Interaction-1**	-8.34	-7.54	-0.80
**Interaction-2**	-9.94	-9.58	-0.36
**Interaction-3**	-3.53	-3.40	-0.14
		MAE	0.43

Table S10 – Nm interaction energies (kcal mol^{-1}).

Complex	ΔE (HF)	ΔE (CGenFF)	ΔΔE
**Interaction-1**	-5.57	-5.40	-0.17
**Interaction-2**	-4.02	-3.91	-0.11
		MAE	0.14

Table S11 – Nn interaction energies (kcal mol^{-1}).

Complex	ΔE (HF)	ΔE (CGenFF)	ΔΔE
**Interaction-1**	-11.75	-11.87	0.13
**Interaction-2**	-11.19	-11.06	-0.13
		MAE	0.13

Table S12 – Nq interaction energies (kcal mol^{-1}).

Complex	ΔE (HF)	ΔE (CGenFF)	ΔΔE
**Interaction-1**	-8.32	-8.20	-0.13
**Interaction-2**	-14.13	-12.32	-1.81
**Interaction-3**	-11.66	-11.85	0.19
		MAE	0.71

Table S13 – Nse interaction energies (kcal mol^{-1}).

Complex	ΔE (HF)	ΔE (CGenFF)	ΔΔE
**Interaction-1**	-8.38	-8.37	-0.01
		MAE	0.01

Table S14 – Nt interaction energies (kcal mol^{-1}).

Complex	ΔE (HF)	ΔE (CGenFF)	ΔΔE
**Interaction-1**	-6.35	-6.61	0.26
**Interaction-2**	-8.69	-8.37	-0.32
**Interaction-3**	-7.92	-7.99	0.06
		MAE	0.21

Table S15 – Nw interaction energies (kcal mol^{-1}).

Complex	ΔE (HF)	ΔE (CGenFF)	ΔΔE
**Interaction-1**	-13.46	-13.36	-0.10
		MAE	0.10

Table S16 – Nwe interaction energies (kcal mol^{-1}).

Complex	ΔE (HF)	ΔE (CGenFF)	ΔΔE
**Interaction-1**	-6.81	-6.85	0.04
		MAE	0.04

Table S17 – Ny interaction energies (kcal mol^{-1}).

Complex	ΔE (HF)	ΔE (CGenFF)	ΔΔE
**Interaction-1**	-6.06	-6.17	0.11
**Interaction-2**	-9.74	-9.92	0.18
		MAE	0.14

1.2.4 Charged Side Chains

Of the six charged monomers parameterized in this work the MAE values for the lysine analogues are least good, with a very large MAE for Nke of up to 4.24 kcal/mol (Figure S5a). This difference

exists through the presence of strong intramolecular hydrogen bonds between these side chains and the backbone carbonyl group in the optimized geometries. In this case it was also found that the only hydrogen bond interactions which could be stably optimized at the HF/6-31G(d) level for the system were those in which the TIP3P water molecule interacted strongly with the amine protons only. The polarization of proton partial charges made it difficult to fix the same value for each proton in a symmetric manner, which was necessary to fix partial charges also. Consequently, when one proton reproduced the energy within the required CGenFF limits, the other significantly disagreed.

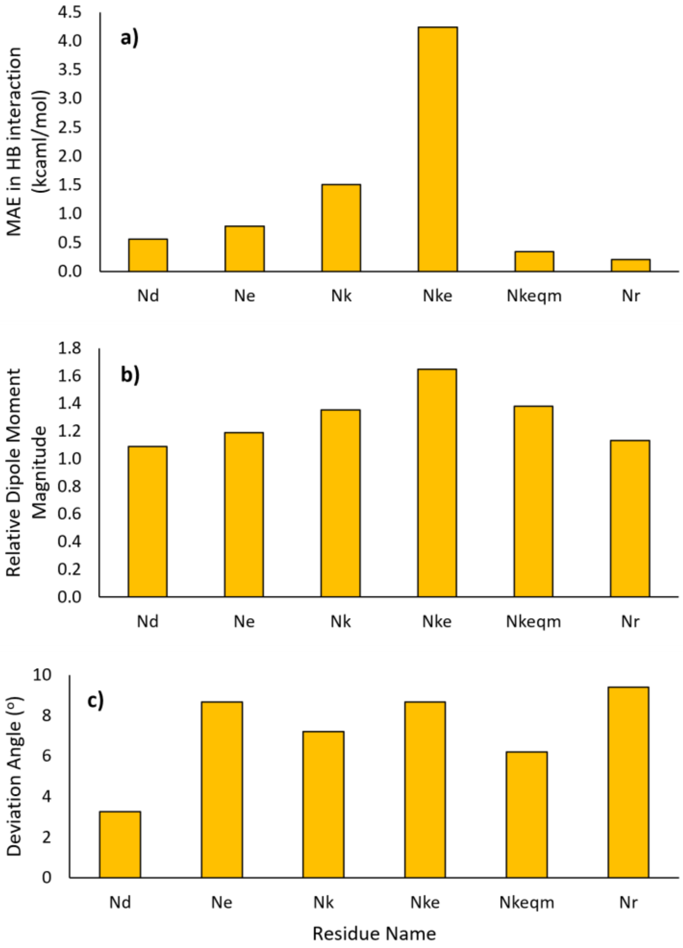

Figure S5. MAE between water interaction energy via calculated partial charges and HF/6-31G(d) optimized structures, for multiple hydrogen bonding complexes a), relative dipole moment magnitude per polar side chain residue. Ideal CGenFF overestimation range, by a factor by a factor of 1.2 to 1.5 b), angle of deviation between dipole moment vector for calculated and HF/6-31G(d) partial charges for charged residues c).

As an alternate strategy, partial charge parameterization for Nk/Nke instead began from the CHARMM22 lysine protonated amine group. The energy deviation was reduced as much as possible, whilst retaining the same kind of partial charge structuring. It was assumed that this would be conserved between these side chain types. On this basis, energy was not strongly weighted as a metric of parameterization quality and instead dipole moment parameters were considered. The same superposition of partial charge arrangement was also the cause of the less than satisfactory MAE for Ne.

As discussed, the dipole moment magnitude and angle of deviation were important parameters for validating the lysine derivatives. It is evident in Figure S5b that while these values are not ideal, they are reasonable. Note Nke has a worse relative dipole moment magnitude than Nk though this is still reasonable. The requirement to reproduce dipole moment parameters for charged groups is not explicitly stated in the CGenFF protocol, though it remains as an intrinsically good indicator of partial charge set quality.

Perhaps the best result for the charged residues is their dipole moment vector deviation angles, with an average of 7.2 ± 2.1° (Figure S5c). The high quality of the orientation reproduction likely arises from the fact that partial charges are typically large and concentrated on charge bearing atoms; this likely outweighs the other charge-coordinate contributions of atoms, which constitute a greater proportion of the dipole moment forming centers (*i.e.*, when everything is less polarized the weighting of dipole moment contributions is more disperse and therefore leads to potentially more angle deviation). It can be concluded that these partial charges are sufficient to represent these charged peptoid residues.

Table S18 – Nd interaction energies (kcal mol^{-1}).

Complex	ΔE (HF)	ΔE (CGenFF)	ΔΔE
**Interaction-1**	-19.30	-19.86	0.57
**Interaction-2**	-15.41	-14.86	-0.56
		MAE	0.56

Table S19 – Ne interaction energies (kcal mol^{-1}).

Complex	ΔE (HF)	ΔE (CGenFF)	ΔΔE
**Interaction-1**	-19.38	-20.16	0.78
**Interaction-2**	-16.92	-16.13	-0.79
		MAE	0.78

Table S20 – Nk interaction energies (kcal mol^{-1}).

Complex	ΔE (HF)	ΔE (CGenFF)	ΔΔE
**Interaction-1**	-15.36	-16.98	1.62
**Interaction-2**	-15.25	-16.65	1.40
		MAE	1.51

Table S21 – Nke interaction energies (kcal mol^{-1}).

Complex	ΔE (HF)	ΔE (CGenFF)	ΔΔE
**Interaction-1**	-11.08	-17.82	6.73
**Interaction-2**	-15.52	-17.27	1.74
		MAE	4.24

Table S22 – Nkeqm interaction energies (kcal mol^{-1}).

Complex	ΔE (HF)	ΔE (CGenFF)	ΔΔE
**Interaction-1**	-10.15	-10.28	0.13
**Interaction-2**	-11.55	-11.74	0.19
**Interaction-3**	-9.82	-10.52	0.70
		MAE	0.34

Table S23 – Nr interaction energies (kcal mol^{-1}).

Complex	ΔE (HF)	ΔE (CGenFF)	ΔΔE
**Interaction-1**	-16.41	-16.13	-0.28
**Interaction-2**	-15.97	-16.10	0.13
**Interaction-3**	-17.62	-17.82	0.20
		MAE	0.20

1.2.5 Charged Termini

We performed partial charge optimization for a series of charged N-termini with either secondary (Nf, Nk and Nq) or tertiary sidechains (Nfes and Ni). In our effort we sought to create a general set which might be used as a 'patch' generally for a given peptoid termini and so the optimization was done in such a way as to generalize across residues. Therefore, specific interactions meant that the MAE in water – complex interaction energies are in some cases significantly greater than the target ± 0.2 kcal/mol bounds (*e.g.*, Figure 6a, Nq).

These cases once again highlight the inherent approximation that partial charges represent and that fitting them can be challenging for multiple interaction complexes which differ greatly in the organization of electron density. It is notable however, the degree of dipole moment overestimation (Figure 6b) is good, with between 1.2 and 1.5 for almost all termini except for Nfes. Also, the angle between the calculated and HF/6-31G(d) dipole moment is also generally around or less than 20° as required for a good partial charge set (Figure 6c).

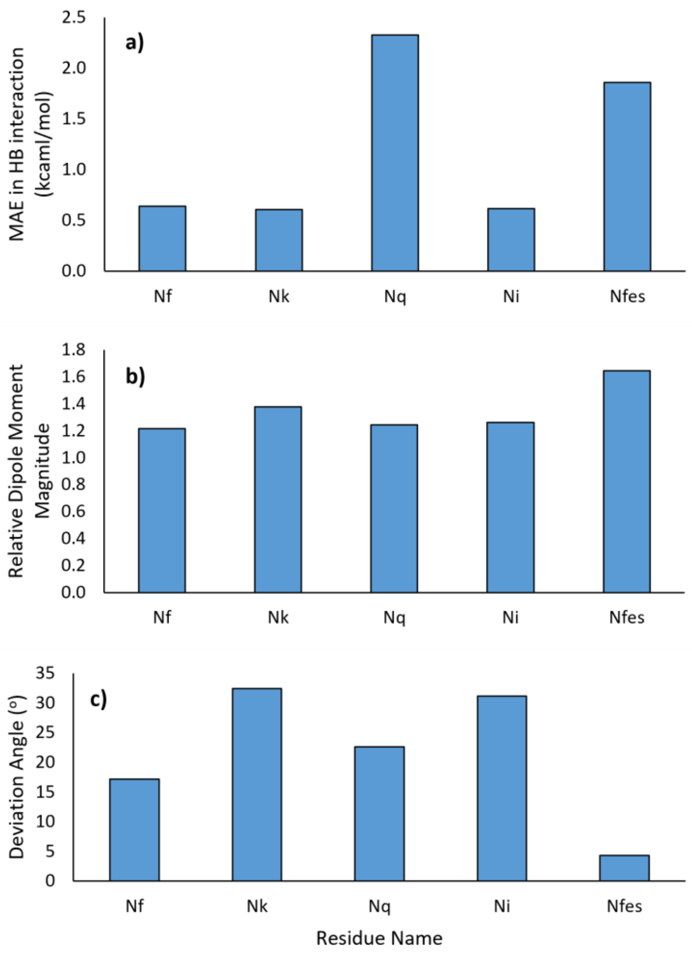

Figure S6. MAE between water interaction energy via calculated partial charges and HF/6-31G(d) optimized structures for charged termini, for multiple hydrogen bonding complexes a), relative dipole moment magnitude per polar side chain residue. Ideal CGenFF overestimation range, by a factor by a factor of 1.2 to 1.5. b), angle of deviation between dipole moment vector for calculated and HF/6-31G(d) partial charges for charged residues c).

Table S24 – Nf N-terminus interaction energies (kcal mol^{-1}).

Complex	ΔE (HF)	ΔE (CGenFF)	ΔΔE
**Interaction-1**	-9.18	-10.62	1.44
**Interaction-2**	-12.58	-12.48	-0.10
**Interaction-3**	-15.12	-15.50	0.38
		MAE	0.64

Table S25 – Nk N-terminus interaction energies (kcal mol^{-1}).

Complex	ΔE (HF)	ΔE (CGenFF)	ΔΔE
**Interaction-1**	-20.72	-20.11	-0.61
**Interaction-2**	-20.72	-20.11	-0.61
		MAE	0.61

Table S26 – Nq N-terminus interaction energies (kcal mol^{-1}).

Complex	ΔE (HF)	ΔE (CGenFF)	ΔΔE
**Interaction-1**	-12.88	-12.34	-0.55
**Interaction-2**	-11.20	-17.11	5.91
**Interaction-3**	-15.99	-15.47	-0.52
		MAE	2.33

Table S27 – Ni N-terminus interaction energies (kcal mol^{-1}).

Complex	ΔE (HF)	ΔE (CGenFF)	ΔΔE
**Interaction-1**	-16.57	-16.13	-0.44
**Interaction-2**	-11.63	-12.47	0.84
**Interaction-3**	-16.27	-15.70	-0.57
		MAE	0.62

Table S28 – Nfes N-terminus interaction energies (kcal mol^{-1}).

Complex	ΔE (HF)	ΔE (CGenFF)	ΔΔE
**Interaction-1**	-9.18	-13.50	4.32
**Interaction-2**	-12.65	-12.11	-0.53
**Interaction-3**	-14.63	-13.90	-0.73
		MAE	1.86

2. Bonded Term Parameterization

2.1 Torsion Optimization Method

In the cases where parameter optimization was required target data was obtained at the MP2/6-31G(d) level of theory using Gaussian16.[5] The torsions for which fitting was done were those which had a penalty score of > 10 during the initial parameter guess when a given structure was uploaded to the CGenFF webpage.[2] Where scores were lower than this it was assumed that the torsion or other parameters for that matter were of a reasonable quality. Each optimized structure, except for structures associated with N-termini, was a single peptoid monomer with an acetylated N-terminus and a dimethylated C-terminus. An example, acetyl-sarcosine-N',N'-dimethylamide, is given in Figure S2.

Relaxed torsion scans were done within the range 0 to ± 180° with a step of either 12 ^{o} or 15° both in positive and negative directions (opt=ModRedundant) as consistent with the Forcefield Toolkit (ffTk) methodology. This program was then used to optimize torsions following the methodology outlined in the tutorial associated with this publication.[11] To generate the scan plots illustrated in Section 2.2 the optimised structure of each scan coordinate was extracted from the log file in *.xyz format and converted to *.pdb format using OpenBabel.[12] The energy was then evaluated for this structure with namd 2.14[13] using the optimised parameter set and the torsion were measured using MDAnalysis.[11] In parallel the MP2/6-31G(d) energies and torsion data was obtained using Gaussian16.[5] For comparison the energies were normalized against the minimum energy structure at each level of theory.

Generally, the torsions presented in the parameter file associated with this work (jp3c03725_si_001.txt) are deemed to be of a good quality, however torsion fitting is known to be challenging and room for improvement exists. Therefore, we regard this parameter set as a starting point from which the peptoid can build ever improving atomistic models. Each torsion plot is labelled with respect to the atoms within a given residue that it represents, for comprehension of these torsions we refer the reader to the topology file associated with these residues (jp3c03725_si_002.txt).

2.2 Torsion Optimization Results

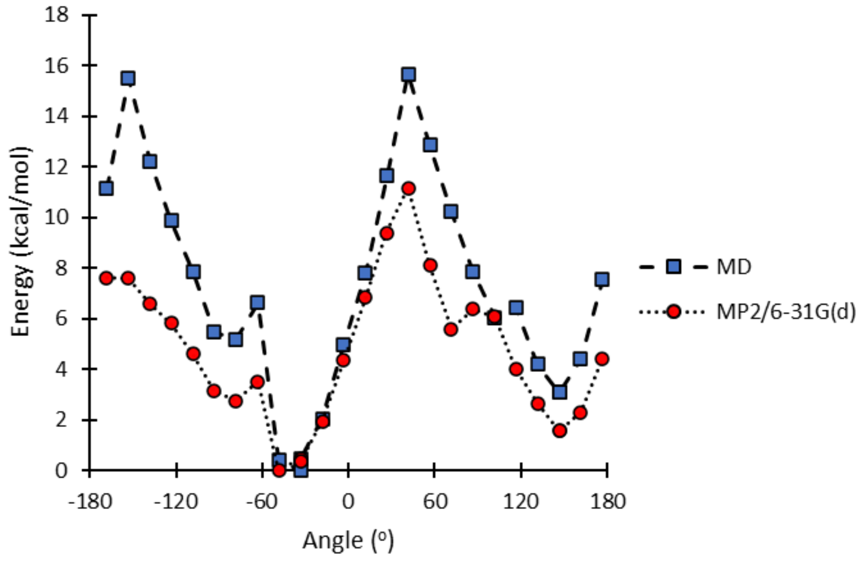

Figure S7. Nar - CG2O1-CG311-NTOID-CG331.

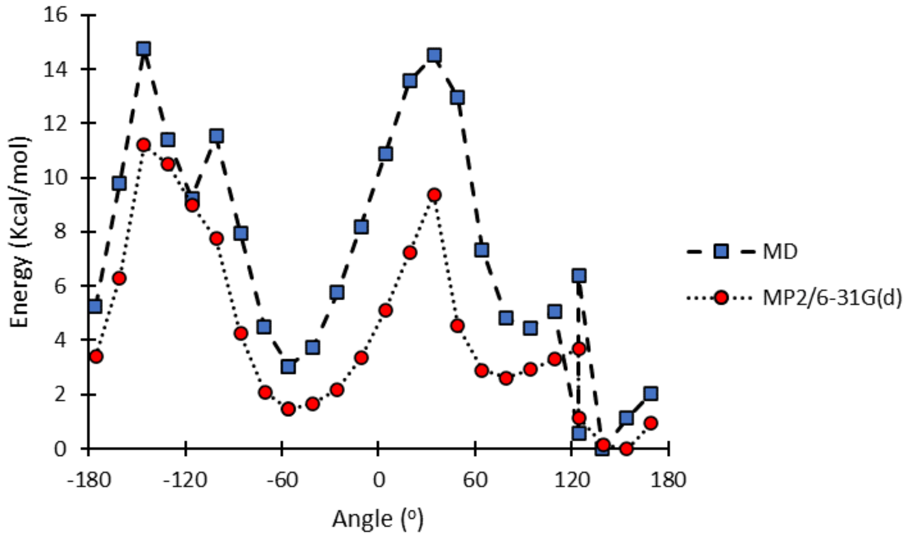

Figure S8. Nar - CG2O1-CG311-NTOID-CG2O1.

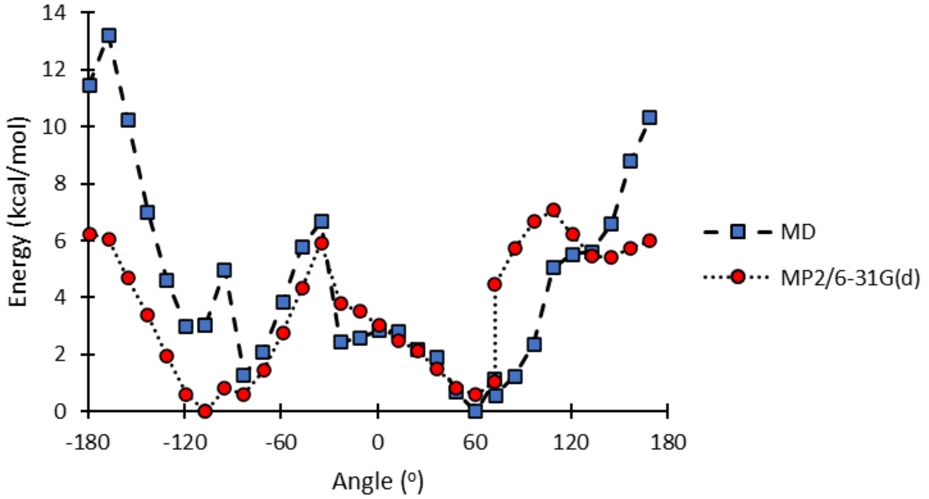

Figure S9. Nfn - CG2O1-CG321-NTOID-CG2R61.

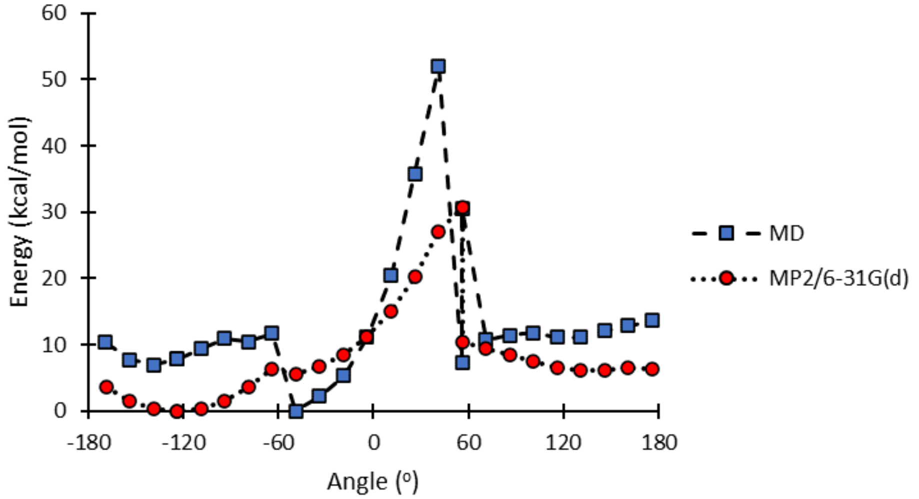

Figure S10. Nke - CG321-NTOID-CG321-CG324.

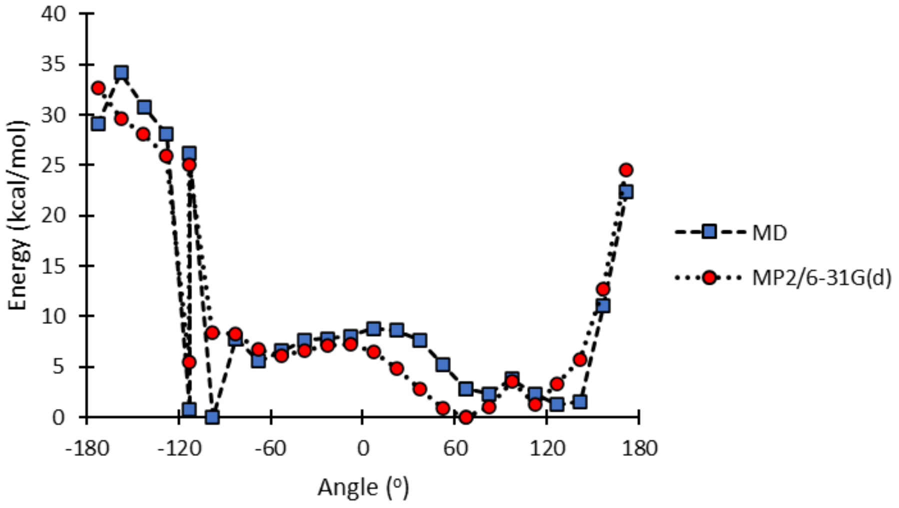

Figure S11. Nke - CG2O1-NTOID-CG321-CG324.

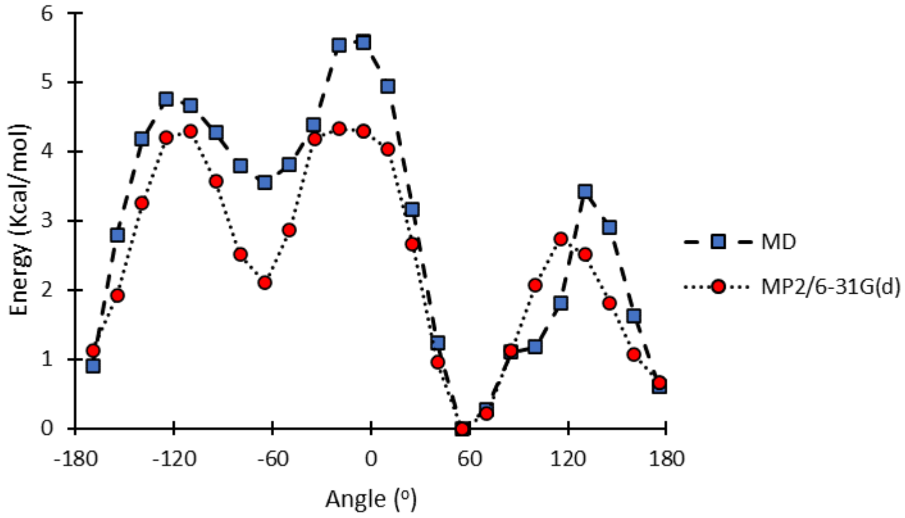

Figure S12. Nl - NTOID-CG321-CG311-CG331.

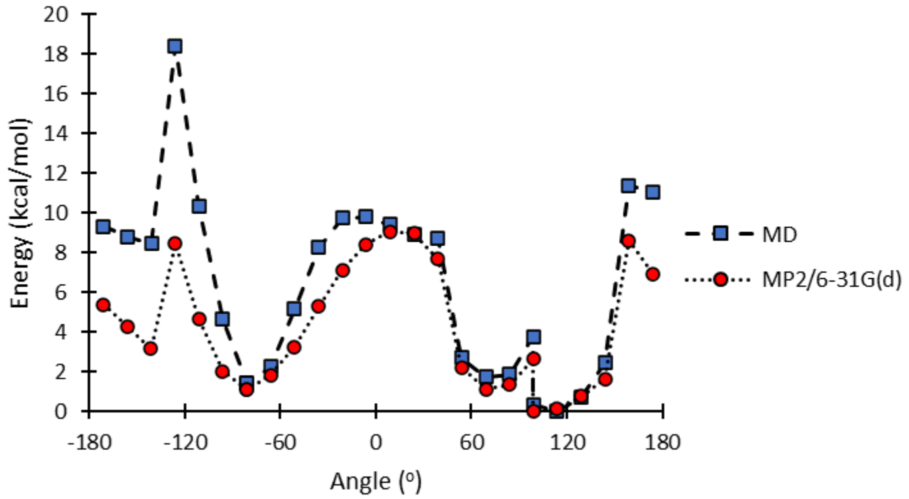

Figure S13. NI - CG2O1-NTOID-CG321-CG311.

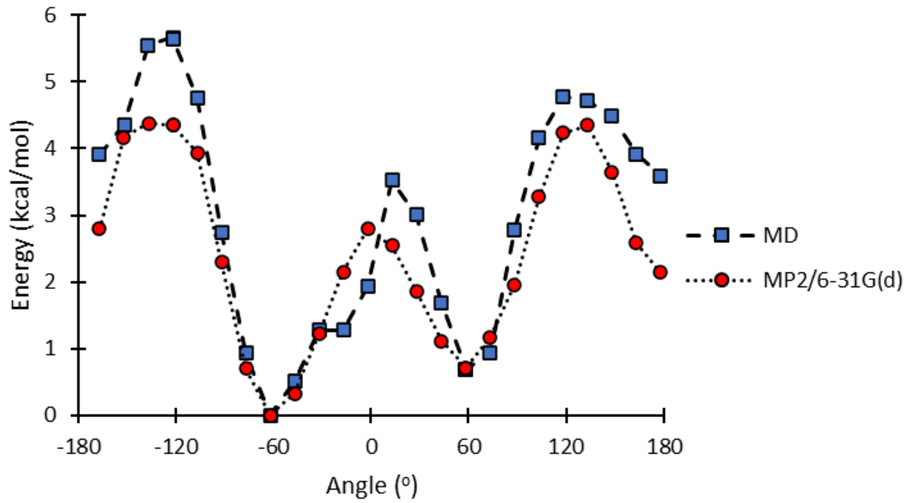

Figure S14. NI - NTOID-CG321-CG311-HGA1.

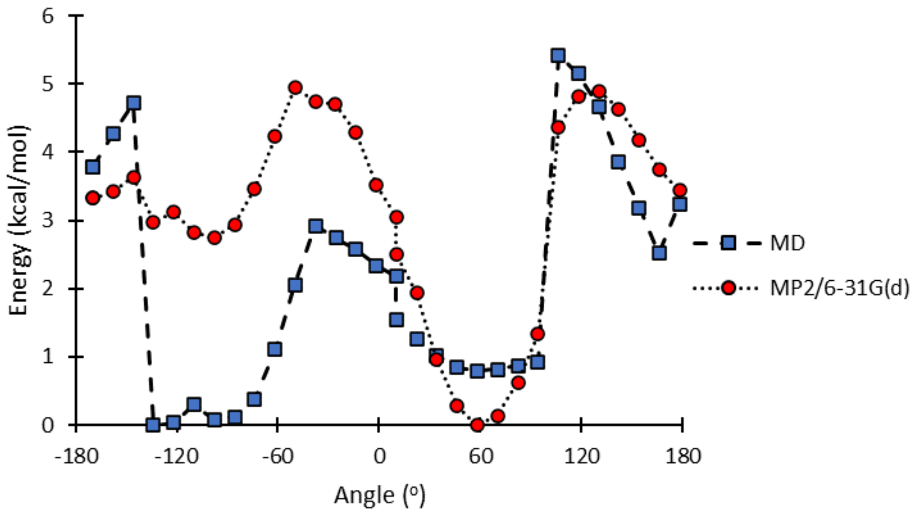

Figure S15. Nt - HGP1-OG311-CG311-NTOID.

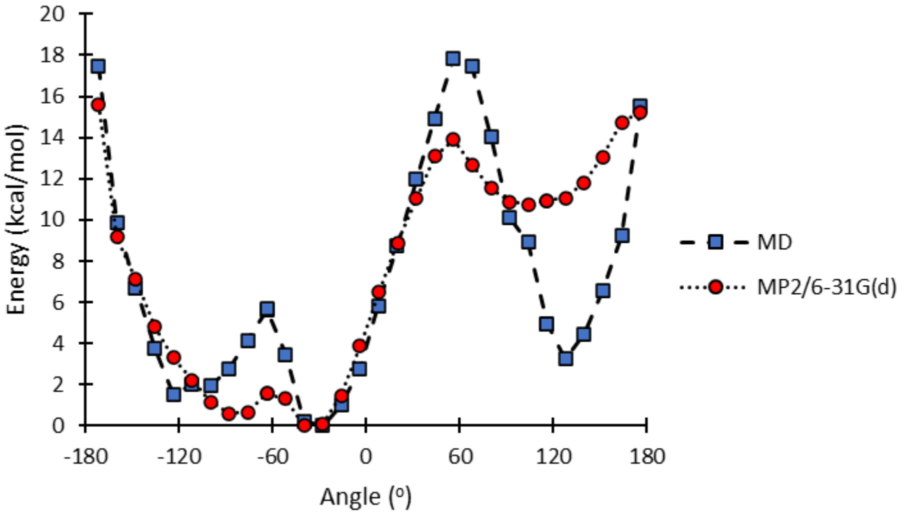

FigureS16. Nt - CG331-CG311-NTOID-CG321.

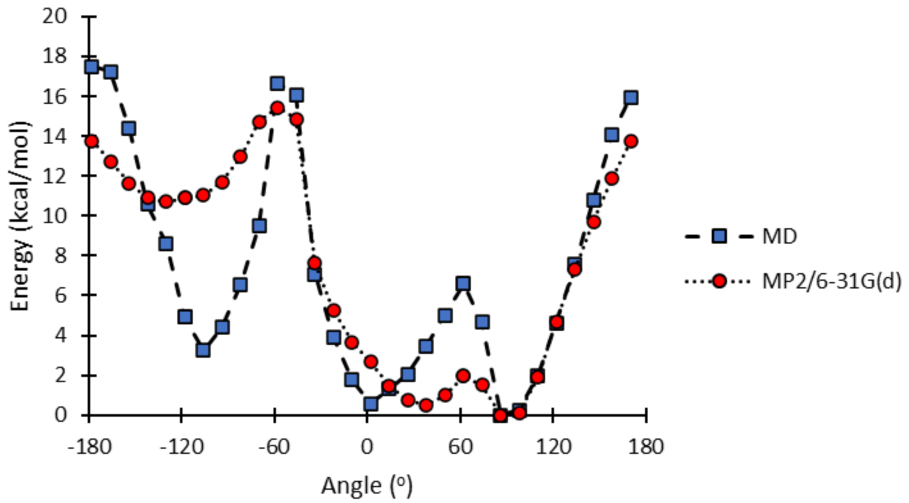

Figure S17. Nt - CG331-CG311-NTOID-OG311.

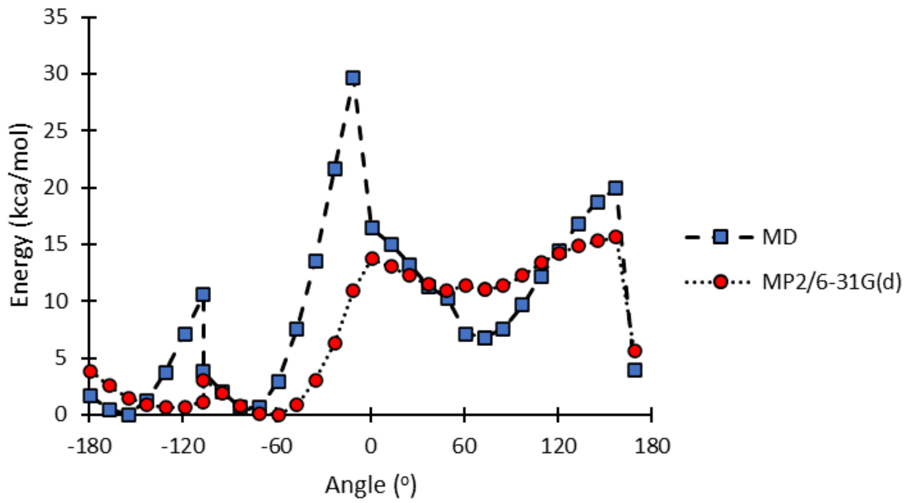

FigureS18. Nt - OG311-CG311-NTOID-CG2O1.

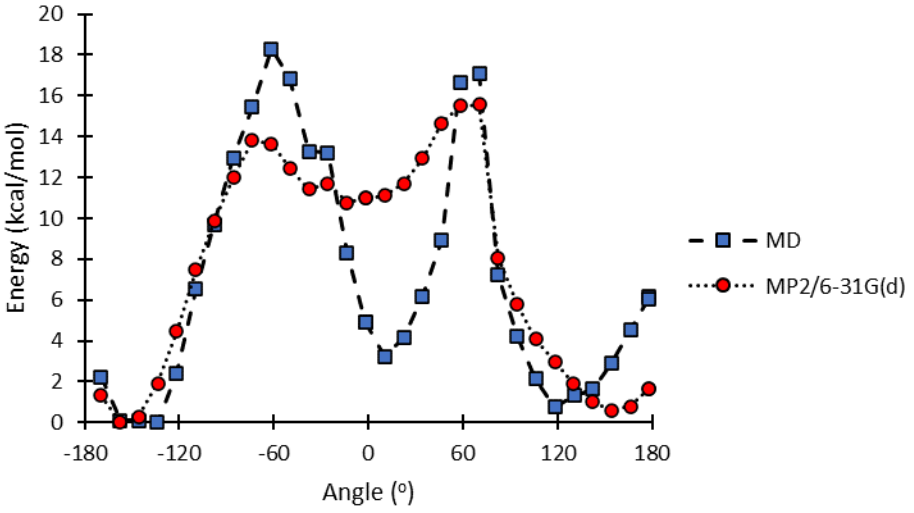

Figure S19. Nt - HGA1-CG311-NTOID-CG321.

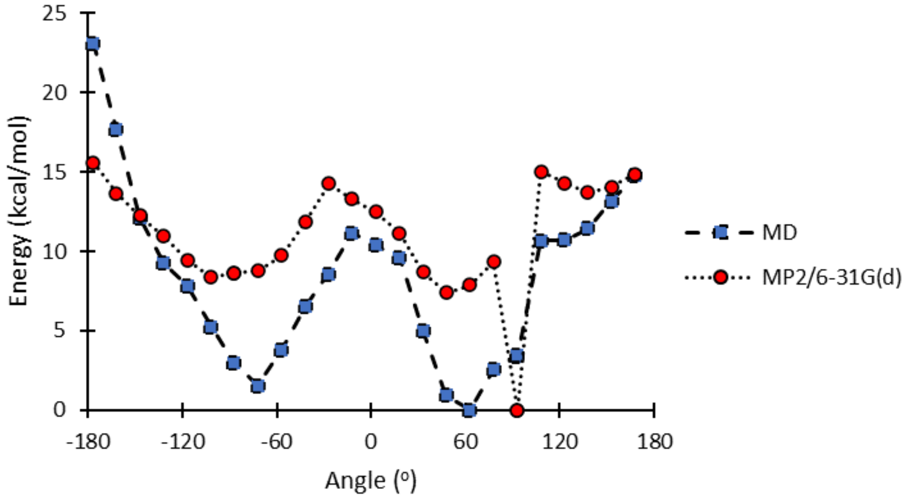

FigureS20. Nt - CG2O1-CG321-NTOID-CG311.

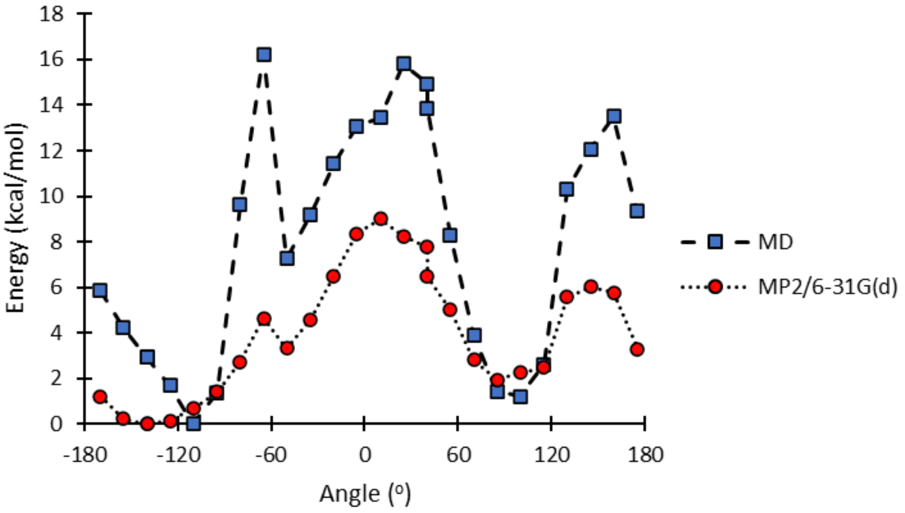

FigureS21. Nw - CG2O1-NTOID-CG321-CG2R51.

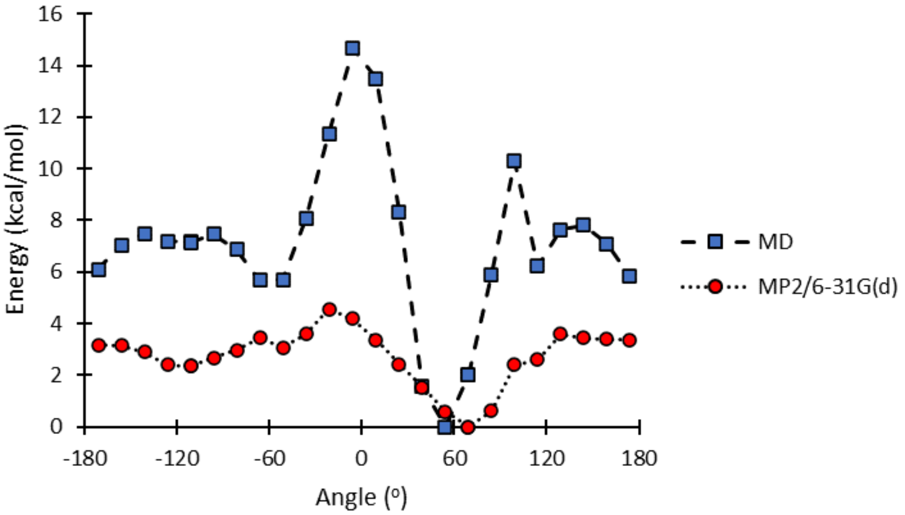

Figure S22. Nw - NTOID-CG321-CG2R51-CG2RC0.

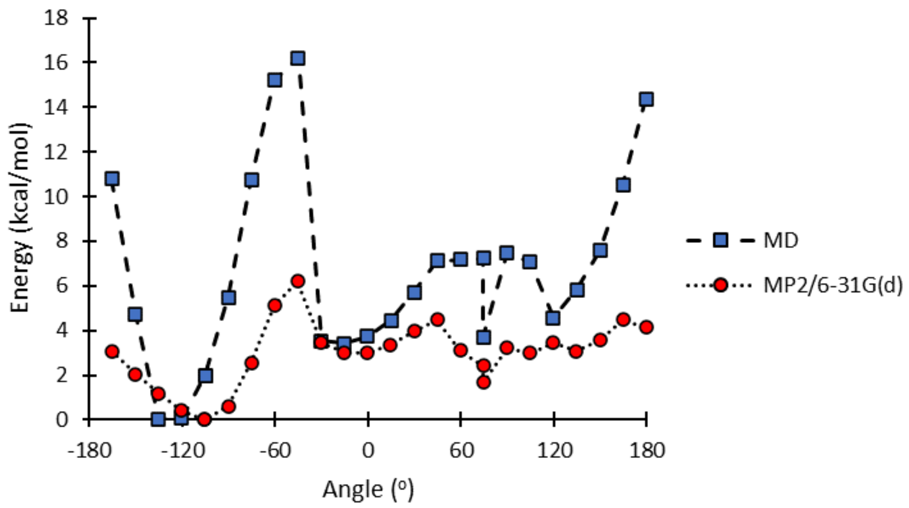

FigureS23. Nw - NTOID-CG321-CG2R51-CG2R61.

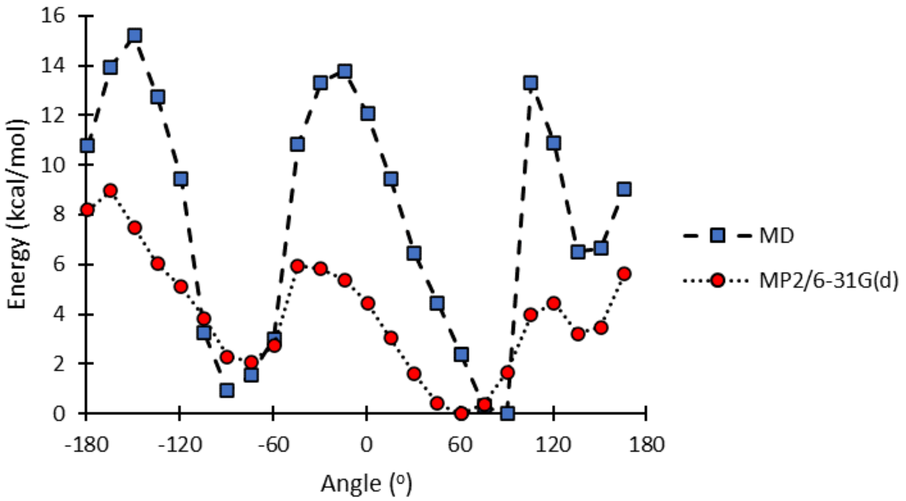

Figure S24. Nw - CG321-NTOID-CG321-CG2R51.

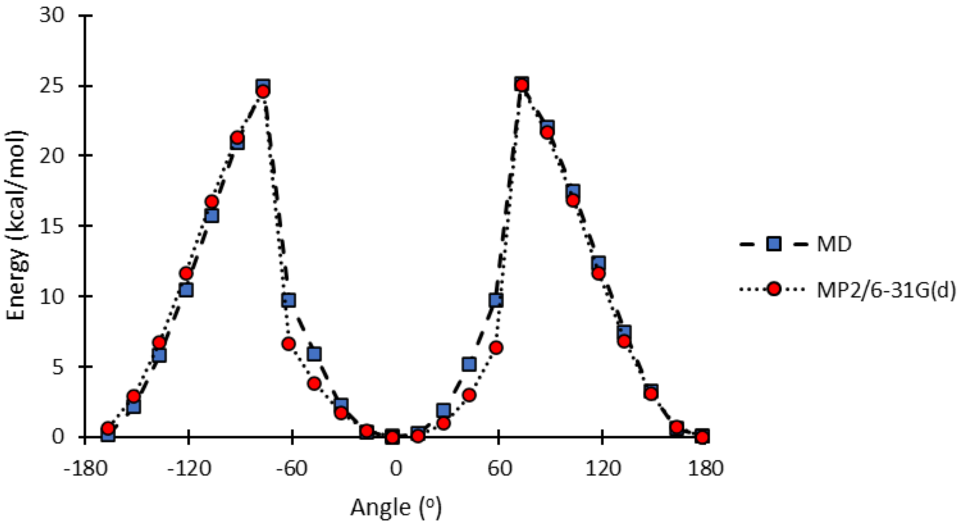

Figure S25. Nf (N-terminus) - CG324-CG2O1-NTOID-CG331 (to represent next sequence CG321).

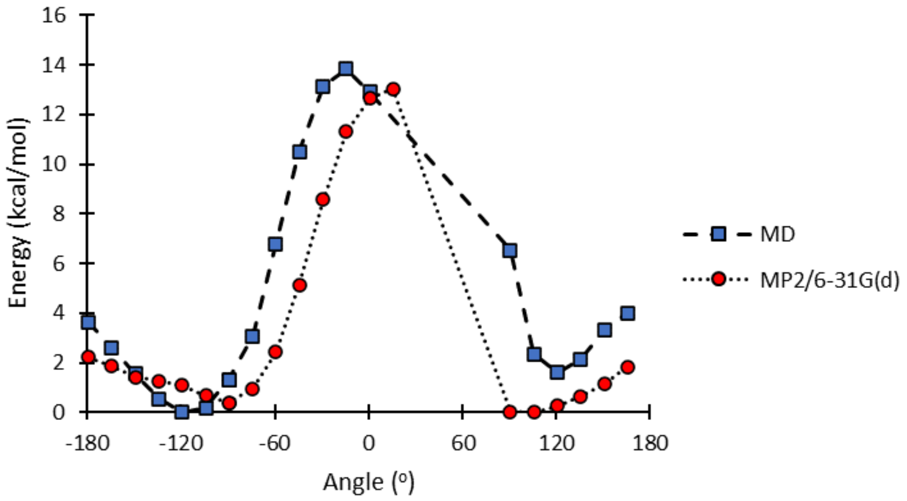

Figure S26. Nf (N-terminus) - CG2O1-CG324-NG3P2-CG324 (Note: no stable scan coordinates from 13 - 90º, though sampling of this dihedral and ANI potential are consistent; refinement of this torsion will be the focus of further work).

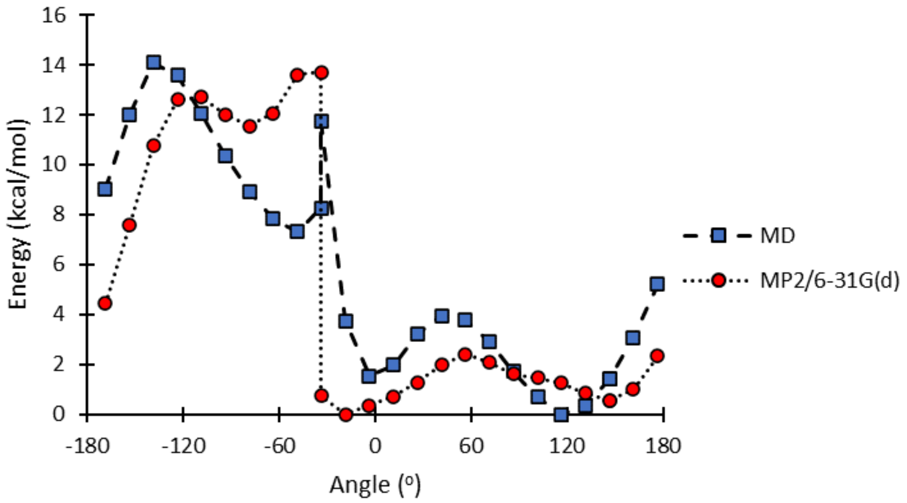

Figure S27. Nf (N-terminus) - CG2O1-CG324-NG3P2-HGP1.

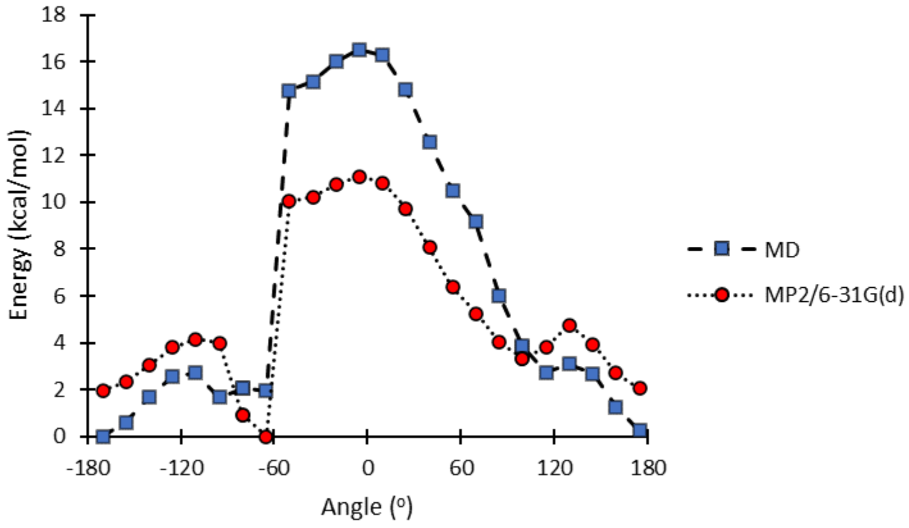

Figure S28. Nk (N-terminus) - CG324-CG321-CG321-CG324.

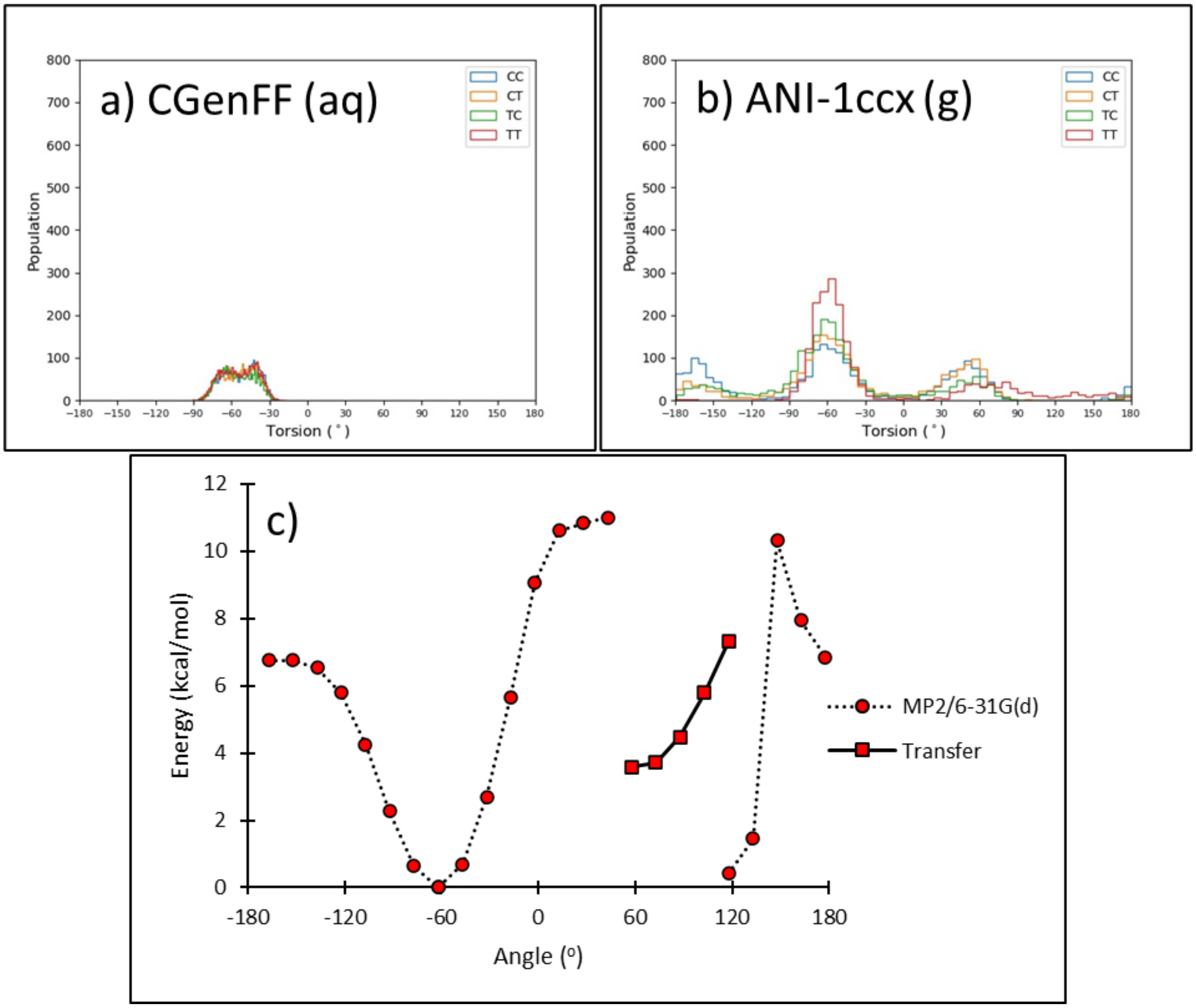

Figure S29. Nke (N-terminus) - HGP1-NG3P2-CG324-CG324. This torsion pertains to rotation about the protonated N-terminus. The CGenFF potential used samples an angle of ~ -60° for all backbone states, a) which is one of the two minima found in the gas phase ANI-1ccx 1 ns simulation for all backbone states of Nke-Nf-Nf b) as further validation that this sampling is appropriate, we analyzed the MP2/6-31G(d) scan and found -60° to be a minimum for this torsion. (Note: it was identified that a proton transfer (N-terminus to adjacent carbonyl) within the 60° – 120° range (squares and solid line) occurred, which cannot occur in the atomistic simulation and so these datapoints are ignored in the scan.

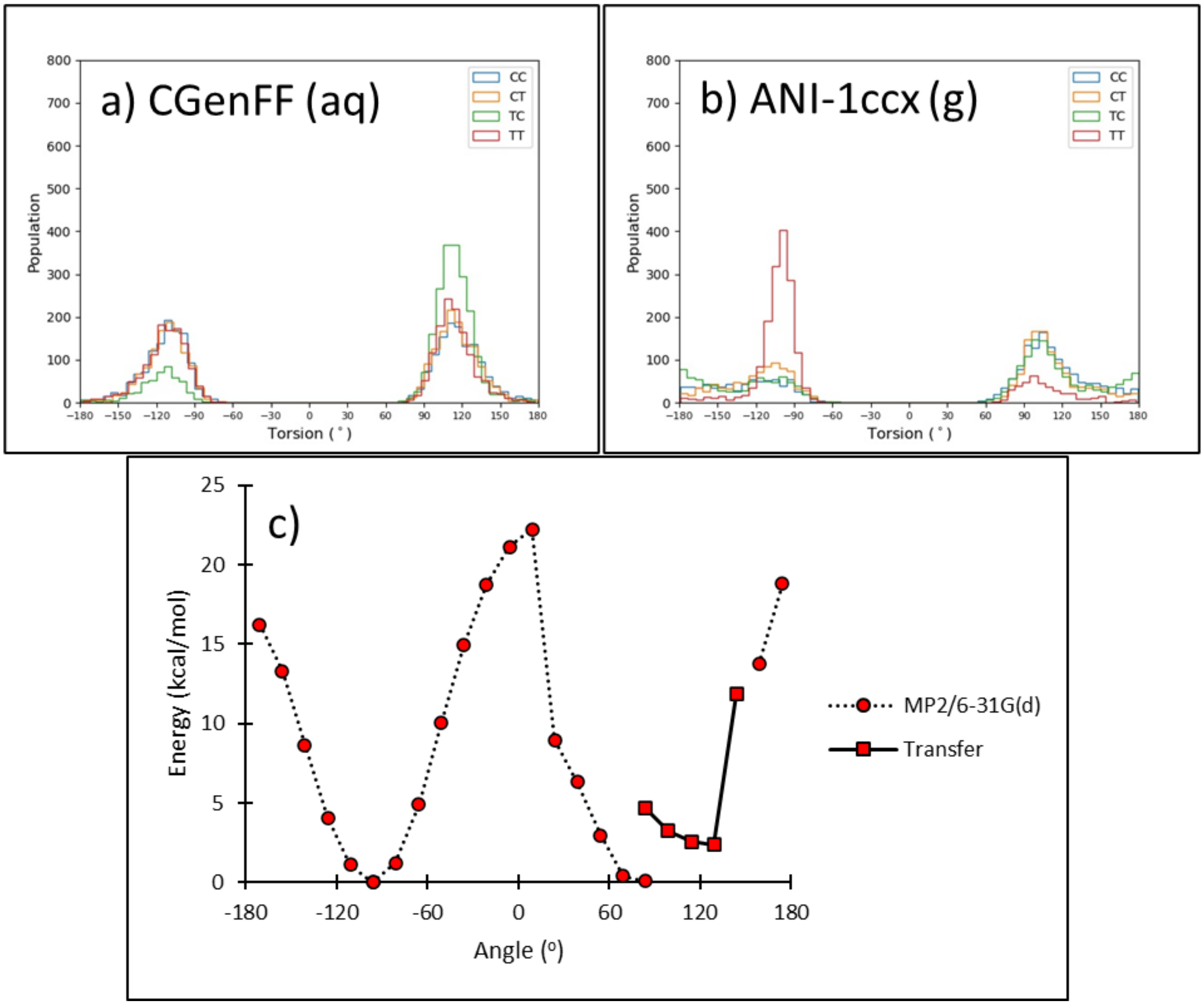

Figures S30. Nke (N-terminus) - CG324-NG3P2-CG324-CG2O1. The CGenFF potential used samples an angle of ~± 120° for all backbone states a), while for the ANI-1ccx 1 ns simulation for all backbone states of Nke-Nf-Nf samples the same minima meaning that the torsion potential used here is an accurate description of higher-level energies b). The MP2/6-31G(d) has similar torsion minima which is further validation of this selection c), however a proton transfer occurred (N-terminus to adjacent carbonyl) within the 60° – 120° range (squares and solid line) and so detail is missing from this segment of the PES.

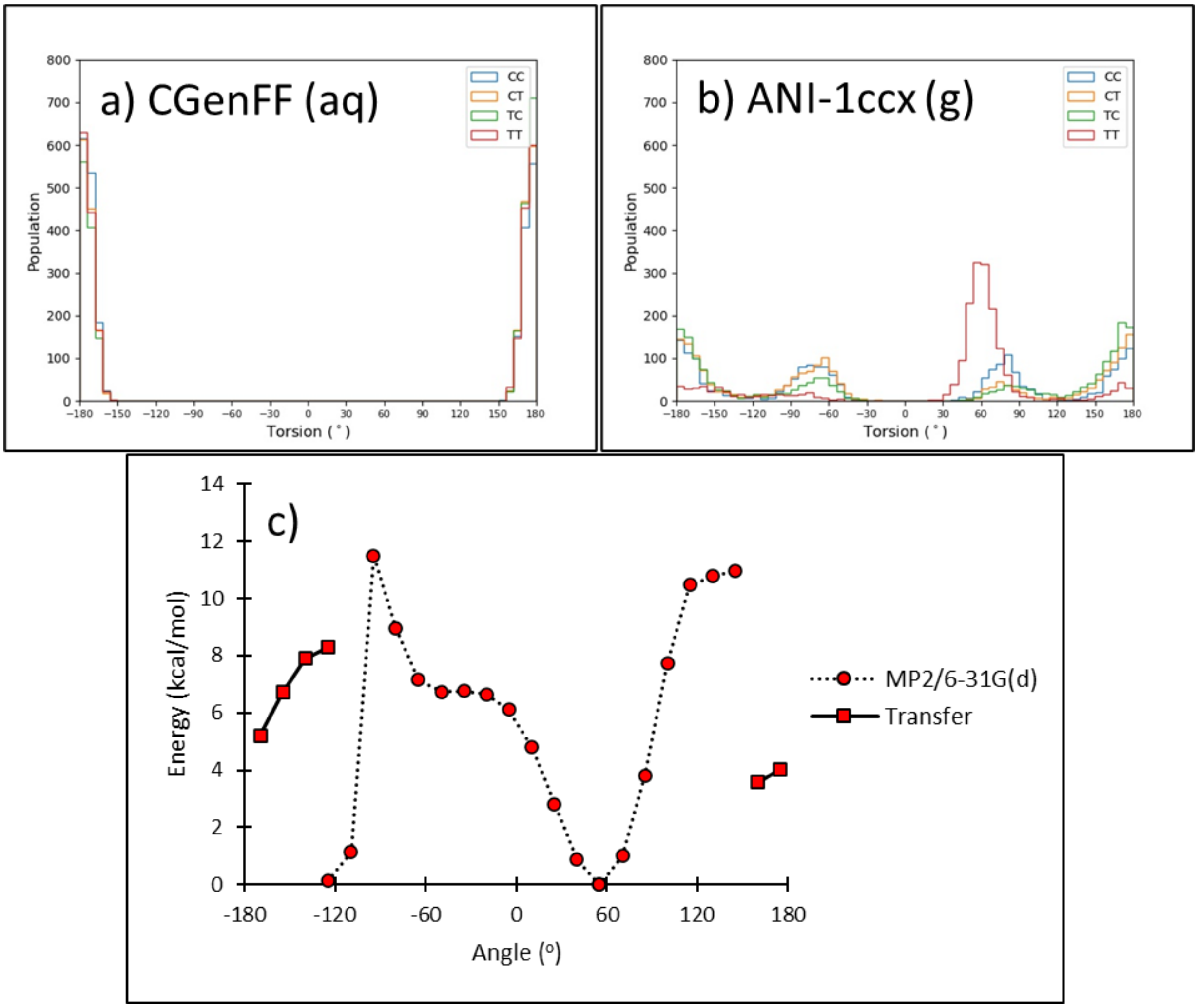

FigureS31. Nke (N-terminus) - CG324-NG3P2-CG324-CG324. The CGenFF potential used samples an angle of $\pm$ 180º for all backbone states a), while for the ANI-1ccx 1 ns simulation for all backbone states of Nke-Nf-Nf samples both $\pm$ 60º and $\pm$ 180º, meaning that the former minima are missed with our torsion potential. This acute torsion corresponds to a hydrogen bond between the protonated amine on the sidechain and the adjacent carbonyl and the favorability of this in the gas phase is obvious (and supported by the MP2/6-31G(d) scan, c)). However, we anticipate that the extended Nke torsion would likely predominate in solution, with the amine and N-terminus interacting with solvent and so while this may be an area for improvement in our parameter set it is not thought that it will have severe consequences on the results obtained for Nke-Nf-Nf, particularly because Nke is not part of the aromatic pattern.

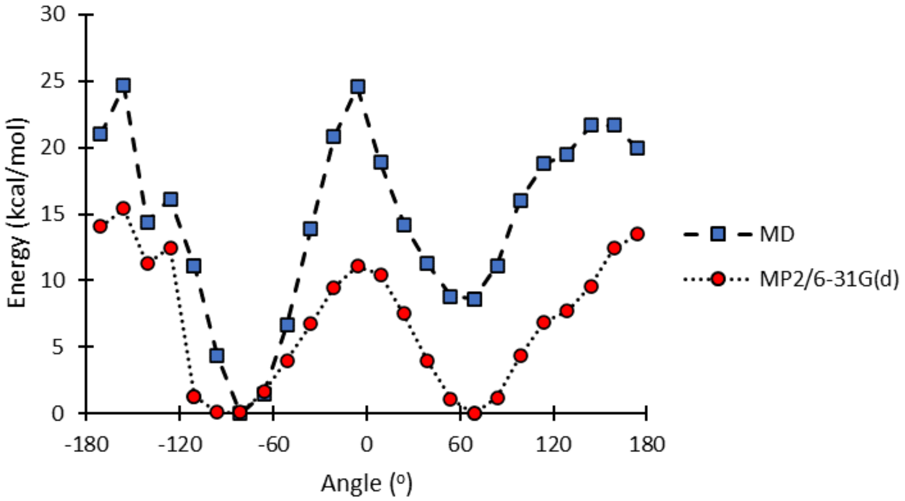

Figure S32. Nke (N-terminus) - NG3P2-CG324-CG324-NG3P3.

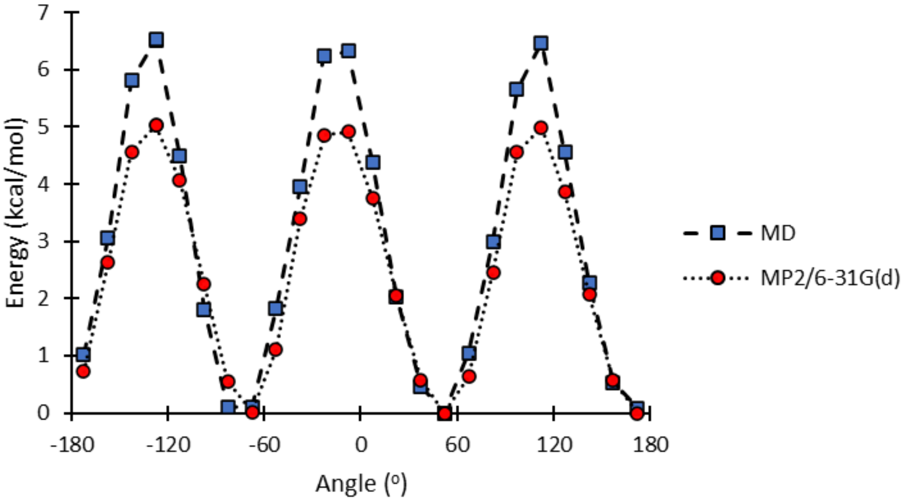

Figure S33. Nke (N-terminus) CG324-CG324-NG3P2-HGP1.

2.3 Dipeptoid ϕ/ψ Sampling Method

2.3.1 Simulation Details

A single dimer of Ac-$(Na)_2$, Ac-$(Nf)_2$ and Ac-$(Nfes)_2$ in either the *cc* or *tt* states were inserted into a 3.8 x 3.8 x 3.8 nm box and solvated with TIP3P water. To generate a Gromacs readable topology file from the CGenFF peptoid parameters the TopoTools VMD plugin was used (command line: `topo writegmxtop`).[14]

The structure was initially minimized using the steep integrator for 100,000 steps (emtol = 100). A Verlet cutoff-scheme was used. Lennard-Jones interactions were switched at 1.0 nm to zero at a cutoff of 1.2 nm (vdw-modifier = force-switch). Electrostatic interactions were calculated beyond the short-range cutoff of 1.2 nm using particle-mesh Ewald (PME) summation, employing a grid spacing of 0.12 nm. Additional setting for CHARMM simulations within Gromacs can be found on the Gromacs website.[15] Gromacs versions 2020.3 and 2020.7 were used for these simulations.[16] These core settings were used in all subsequent steps. All bonds containing hydrogen were constrained using the LINCS algorithm.[17] To ensure adequate coupling of velocities the molecule and solvent were treated as separate coupling groups throughout. The reference pressure was 1.01325 bar in all relevant steps and a temperature of 298.15 K was used throughout.

The system was initially simulated in the NVT ensemble algorithm (1,000,000 steps, ts = 1 fs, tau-t = 1 ps, 1 ns) and initial velocities were generated according to a Maxwell distribution at the given temperature using a random seed. Following this a short 200,000 step NPT ensemble treatment of the system was applied with isotropic pressure coupling using a Parrinello-Rahman barostat with a compressibility of $4.5x10^{-5}$ bar^{-1} (200,000 steps, ts = 1 fs, tau-t = 1 ps, tau-p = 1 ps, duration = 0.2 ns). Both stages made use of the v-rescale algorithm to maintain temperature. A production simulation was then performed with Nose-Hoover temperature coupling (50,000,000 steps, ts = 2 fs, tau-t = 1 ps, tau-p = 2 ps, duration = 100 ns). The system was integrated using the leap-frog algorithm. This approach follows that used by Zhao *et al*. in the simulation of peptoid nanosheets and helices.[18]

2.3.2 Analysis Method

With the simulation data in hand, we then measured the ϕ/ψ preferences. A peptoid backbone can occupy up to 12 relevant minima within the centrosymmetric Ramachandran surface: $C_{7\beta}^{+/-}$, $\alpha_D^{+/-}$ and $\alpha^{+/-}$.[19 20 21] Due to the center of symmetry the plus and minus signs indicate where the backbone conformation sits relative to the line of symmetry. A minus corresponds to a right-handed (RH) structure and a plus corresponds to a left-handed structure (LH).

The first characterization of these preferences at the DFT level was by Moehele and Hoffmann at the MP2/6-31G(d) level of theory,[22] and subsequently other studies, informed by experimental NMR and crystal structures have followed. [19, 20] Recently it has been shown through the thorough exploration of this surface using metadynamics sampling and comparisons to DFT that peptoid forcefields can accurately capture this surface.[21, 23] However arguably the most rigorous analysis was done by Weiser and Santiso, who characterized the complete Ramachandran surface of N-N-dimethylacetamide (Ac-Na-N(CH_3)$_2$) using DFT at the MP2/6-31G(d) level of theory.[1] Given the various sources of Ramachandran minima positions, derived from both computational and experimental studies, we provide estimates from literature of the locations of these minima which guide our analysis of minima sampling from simulations (Table S29).

Table S29 – Ramachandran region minima

**Contributor**	**φ/ψ Minima**			
	α_D(+)	α_D(-)	$C_{7\beta}$(+)	$C_{7\beta}$(-)
Butterfoss *et al*. (ref [19])	90º ,175º	280º,180º	120º,285º	240º,70º
Spencer *et al*. (ref [20])	70º, 180º	290º,180º	-	-
Wesier *et al*. (ref 1)	60º, 180º	270º,180º	130º,290º	230º,65º
Our Test	80º, 175º	280º,180º	120º,290º	230º,70º

With our central positions for the and $\alpha_D^{+/-}$ and $C_{7\beta}^{+/-}$ minima defined we then measured the occupancy of these states by counting all ϕ/ψ combinations from the MD trajectory which fell within a 30° radius of these coordinates.

2.3.3 Dimer Ac-(Na)$_2$

The Ramachandran surface sampling for the first residue of Ac-(Na)$_2$ is qualitatively in good agreement with previous higher level measurements (Figure S34). In the *cis* state (ω ≈ 0°), approximately equal sampling of α_D^+ and α_D^- is observed which would be expected given that these states are isoenergetic (Table S30). Additionally some sampling of $C_{7\beta}^-$ is observed though this is only 4.2 % of measured ψ/ϕ combinations which is consistent with it being a local minima. In the *trans* state (ω ≈ 180°) sampling of all four expected regions is observed which indicates that the distinction in ϕ/ψ sampling with respect to ω is captured. Notably this is not a four way split and that the $C_7^{+/-}$ states are not as readily sampled despite being isoenergetic with α_D^+ and α_D^-. This is thought to be the case for two reasons: (1) the CGenFF torsion potentials used in our model, fit by Weiser and Santiso, have a 2 kcal/mol barrier between $\alpha_D^{+/-}$ and $C_{7\beta}^{+/-}$ states and this may impede exloration of these minima and, (2) the shape of the $C_{7\beta}^{+/-}$ wells are ovular and not perfectly captured with our circular approximation. Despite this we believe that these deficiencies are not sufficeint to preclude the use of this forcefield.

The behaviour of residue two is not equivalent to that of residue one with the *cis* state sampling the local minima α^+ and α^- regions most readily and for the *trans* state the global minima $C_{7\beta}^{+/-}$ is most readily sampled. Previously high level calculations of peptoid ϕ/ψ surfaces have focused on dimethyl capped C-termini while in these instances our model uses an amidated C-terminus,[1, 19] as these termini are less sterically constrained than the former we expect that the Ramachandran surface may be distinct and that a direct comparison is not appropriate. Generating this extra data is not trivial and is beyond the scope of this work. The parameters used for the ϕ and ψ angles in these models are those from Weiser and Santiso's original publication and sampling is qualitaitvely in sensible regions of the Ramachandran surface.

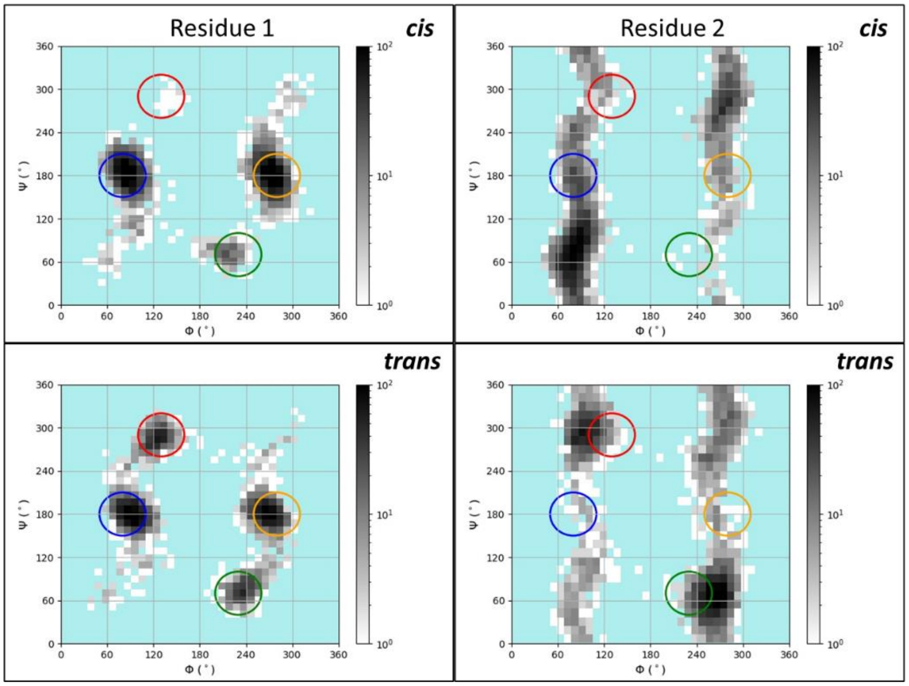

Figure S34. ϕ/ψ sampling for Ac-(Na)$_2$ over 100 ns (4000 frames) showing that for residue 1 in both *cis* and *trans* states are in good agreement with both theoretical and experimental estimates of ϕ/ψ minima. For residue 2 the sampling is distinct from these estimates, though QM data for comparison of this torsion Ramachandran plot is not available at this time for comparison (number of bins = 30). In all cases the blue background is used to show regions which are unsampled in the trajectory.

Table S30 – Ocucpancy of $\alpha_D{}^{+/-}$ and $C_{7\beta}{}^{+/-}$ for residue one of Ac-(Na)$_2$

**Minima Region**	**% occupancy *cis***	**% occupancy *trans***
$\alpha_D{}^{+}$	35.6	30.2
$\alpha_D{}^{-}$	37.8	24.7
$C_{7\beta}{}^{+}$	0.4	14.3
$C_{7\beta}{}^{-}$	4.2	10.9

2.3.4 Dimer Ac-(Nf)$_2$

The ϕ/ψ preferences for Ac-(Nf)$_2$ in the *cc* and *tt* states were also measured (Figure S35). It was found that for the *cis-1* (residue one) sampling of the $\alpha_D{}^{-}$ was dominant, 76 % of all measured points, as expected and in agreement with the Weiser and Santiso Nf representation in TIP3P

water, where $\alpha_D$$^{+/-}$ are the global minima. For the *trans-1* sampling of $\alpha_D$$^-$ should be greater than that of $C_{7\beta}$$^{+/-}$ as the latter is a local minimum. In our representation this behavior is preserved with 54.8 % of all measured points occupying $\alpha_D$$^{+/-}$ regions and 18.8 % sampling $C_{7\beta}$$^{+/-}$ minima (Table S31).

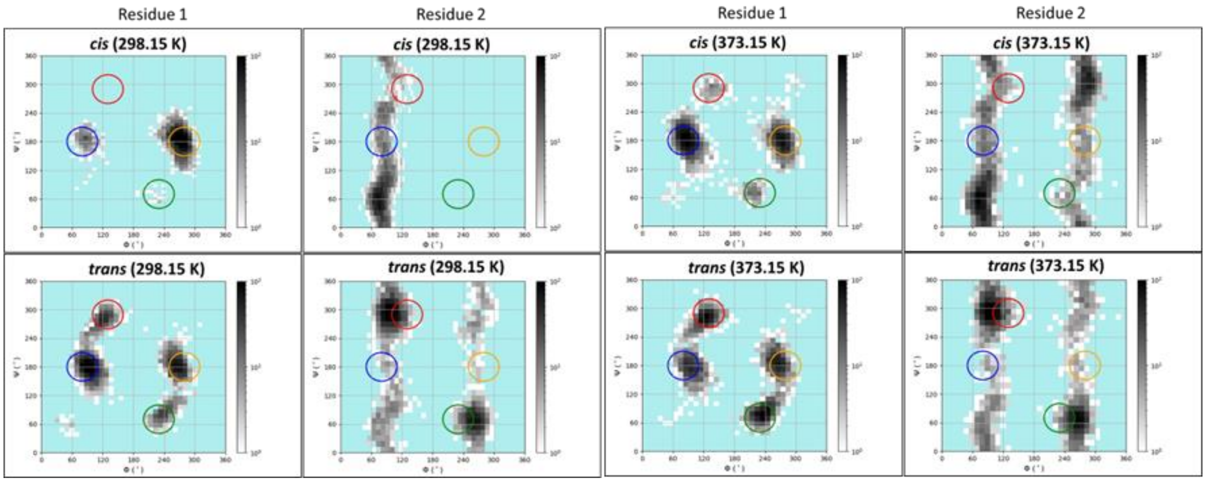

Figure S35. ϕ/ψ sampling for Ac-(Nf)$_2$ over 100 ns (4000 frames) showing that for residue 1 is in good agreement with both theoretical estimates of ϕ/ψ minima identified by Weiser and Santiso. Simulation at 373.15 K confirms that the poor sampling for the *cis* state in 298.15 K is the reason why the sampling of the $\alpha_D$$^-$ region is exaggerated and instead this is not a bias for this state within the model. (number of bins = 30).

The over sampling of the $\alpha_D$$^-$ minima by the *cis-1* was of marginal concern, as it could imply that our representation of Nf was not isoenergetic in $\alpha_D$$^{+/-}$ states. To test this, we increased the system temperature to 373.15 K, to enhance sampling of the Ramachandran surface, and repeated the simulation from the same starting coordinates. In the 373.15 K simulation it was found that for the *cis-1* state, the $\alpha_D$$^{+/-}$ minima was still the major conformer as expected (*e.g.*, 66.4 % of sample points), and of these more than half of samples were in the $\alpha_D$$^+$ minima. This result indicates to us that the states are indeed isoenergetic and that slow exploration of the Ramachandran surface at the lower temperature was the cause for this apparent bias.

For the *trans* state the occupancy of the $\alpha_D$$^{+/-}$ states decreased (29.7 %) and sampling of the local $C_{7\beta}$$^{+/-}$ minima increased to be the major conformer (41.5 %). The change towards increased $C_{7\beta}$$^{+/-}$ sampling with higher temperature agrees with our previous assertion that accessing this minimum from $\alpha_D$$^{+/-}$ conformations is controlled by a barrier between these states.

Table S31 – Occupancy of $\alpha_D^{+/-}$ and $C_{7\beta}^{+/-}$ for residue one of Ac-(Nf)$_2$ at varied temperature.

Temperature	Minima Region	% occupancy *cis*	% occupancy *trans*
**298.15 K**	$\alpha_D{}^+$	7.2	33.8
	$\alpha_D{}^-$	68.8	21.0
	$C_{7\beta}{}^+$	0.0	11.4
	$C_{7\beta}{}^-$	0.5	7.4
**373.15 K**	$\alpha_D{}^+$	41.1	13.6
	$\alpha_D{}^-$	25.3	16.1
	$C_{7\beta}{}^+$	2.8	19.1
	$C_{7\beta}{}^-$	3.4	22.4

It was deemed prudent to assess whether the occupancy of various minima on the φ/ψ surface could impact sampling of the ρ-λ surface, given that our simulations were done at 298.15 K, when the $\alpha_D{}^-$ confromer was the majority sampes minima in the *cis* state. As such ρ-λ plots were compared at both temperatures (Figure S36). The regions of sampling for these descriptors were in good qualitative agreement at both temperatures, however for the λ ~ 90° well in the 298.15 K *cis* conformation is less well sampled than its -90° counterpart which indicates these parameters are correlated. This provides some explanation for the apparent symmetry/asymmetry observed in these plots for tripeptoids (Section 3.1, such as Nf-Nk-Nf (tc)) and the consistent bias towards positive λ values in the tripeptide cases (Section 3.2), where less of the Ramachandran surface can be explored.

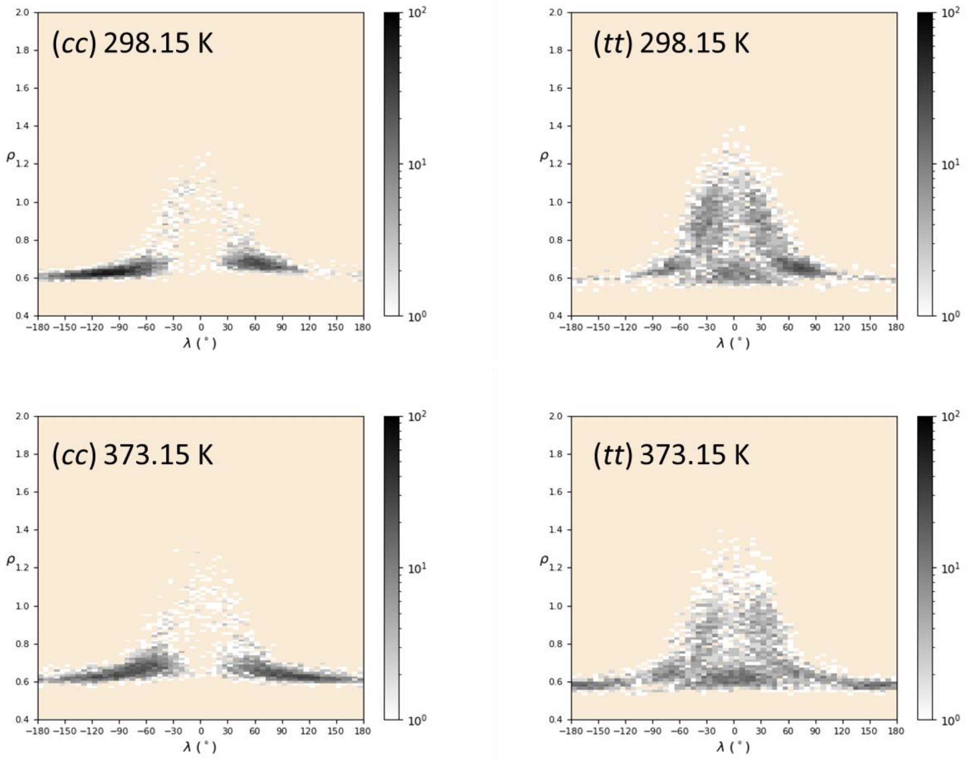

Figure S36. ρ *vs.* λ plots for Ac-Nf-Nf at both 298.15 K (temperature used in dimer and trimer study) and 373.15 K in both *cc* and *tt* states. For *cc* at 298.15 K most of the ϕ/ψ sampling was in the α_D- minima at 298.15 K (Figure S35), whereas at 373.15 K sampling was better balanced between the two minima; irrespective sampling of the ρ vs. λ plots are qualitatively the same indicating that the dynamics of these torsions are not significantly coupled (number of bins = 60).

To address this observation in greater detail we assessed the inter-relation of λ/ψ/ϕ by producing 2d plots of each torsional pair at 298.15 and 373.15 K for cc and tt Ac-$(Nf)_2$. The first cis residue in the cc state shows a λ/ψ density at λ ≈ ± 90°, ψ≈180° at both temperatures, with a more even sampling at the higher temperature. For the λ/ϕ combination we observe four regions, λ ≈ ± 60 – 90° and ϕ ≈ 90° or 270°, where access after 100 ns to ϕ ≈ 90° is limited. Again the use of a higher temperature accelerates access to multiple ϕ states (Figure S37).

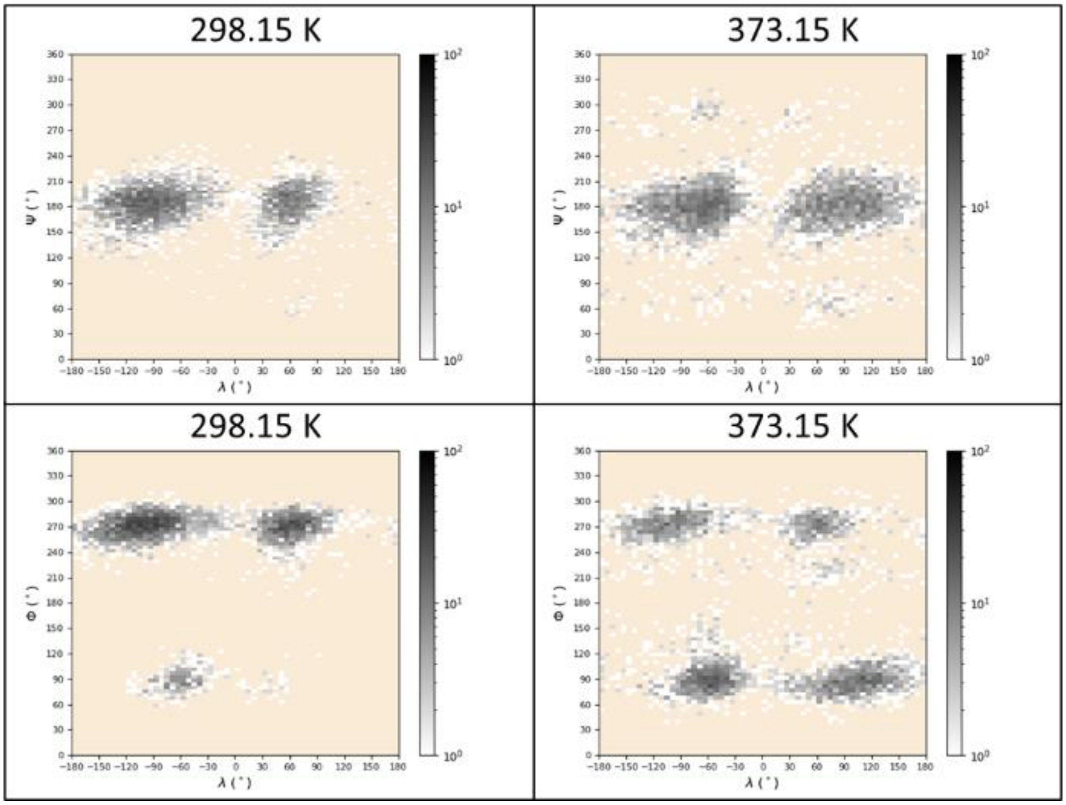

Figure S37. 2D histogram plots of λ/ψ and λ/ϕ for residue 1 in cc Ac-(Nf)2 at 298.15 and 373.15 K in TIP3P water over the duration of 100 ns (bin size = 6°, 4000 frames, 1 molecule). Symmetry is clear in the λ/ϕ torsions however this is not observed in the λ/ϕ dimension due to the superposition of states; evidencing that sampling in λ is fast while sampling in ϕ is comparatively slow and so convergence in λ will occur within 50 ns.

To import further physical insight, we compare all three dimensions at various time points across the 100 ns trajectory (Figure S38, 12.5, 25, 50 and 100 ns). Viewing this plot through the λ/ψ dimension, the quadrants of λ/ϕ state sampling becomes indistinguishable through superposition. The meaning of this is such that while sampling in ϕ may be slow, convergence in the λ dimension will occur comparatively quickly. Indeed, sampling of both λ ≈ ± 90° can be seen to have occured within 50 ns. At timepoints less than this convergence is in progress. By 100 ns access to λ ≈ - 90°, ϕ ≈ 90° which is superimposable on λ ≈ - 90°, ϕ ≈ 270° has been realized. This same outcome is observed at 373.15 K though convergence is naturally faster and more equal across all four quadrants (Figure S39). As the duration of simulation is consistent in the multiple molecule study, we are confident that convergence will have been realised.

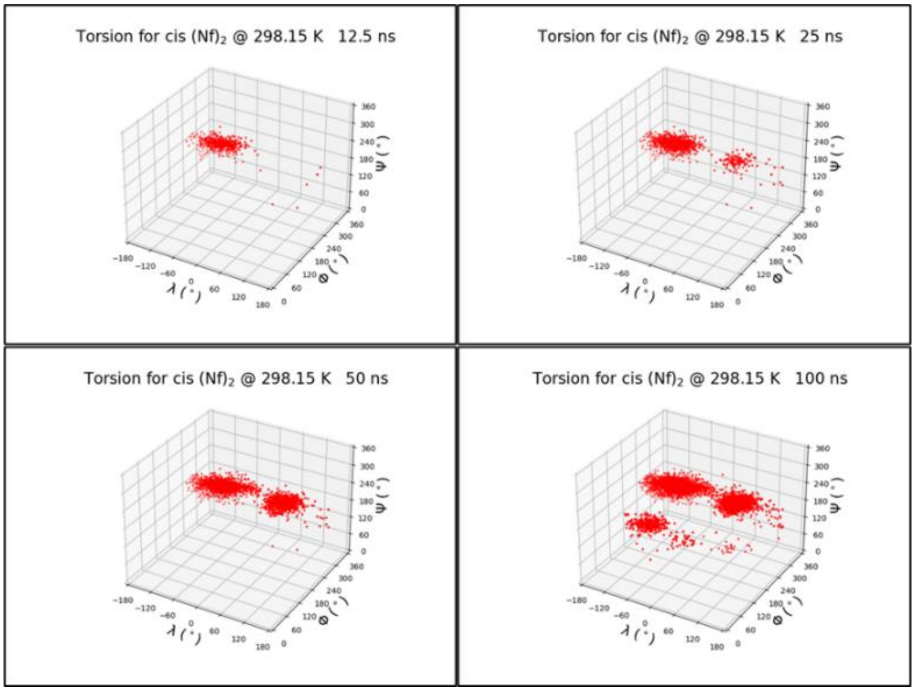

Figure S38. 3D plot of λ/ϕ/ψ states for residue 1 in cc Ac-$(Nf)_2$ at 12.5, 25, 50 and 100 ns in a trajectory at 298.15 K. Here symmetry in λ/ϕ can be seen and the loss of specific ϕ for a given λ when viewed as λ/ψ can be understood at 100 ns. Adequate sampling of both λ states is thus obtained by 50 ns.

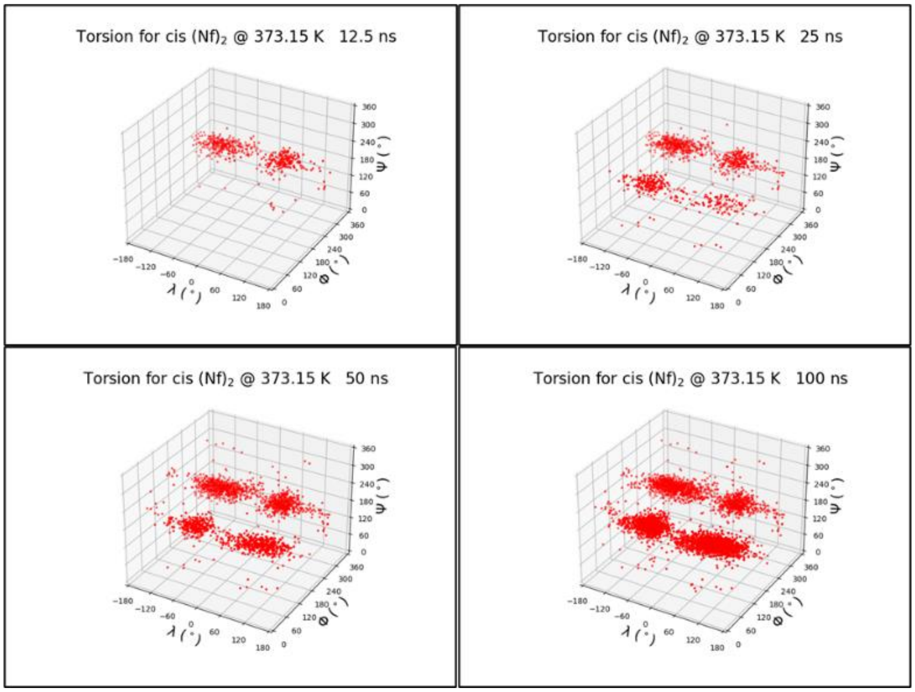

Figure S39. 3D plot of λ/ϕ/ψ states for residue 1 in cc Ac-$(Nf)_2$ at 12.5, 25, 50 and 100 ns in a trajectory at 373.15 K. Confirming the same result as found for the system at 298.15 K, however as expected convergence occurs much faster.

A similar outcome is found for the first trans residue in tt Ac-(Nf)$_2$ when 2d property plots are considered the λ/ψ distributions are centred on ψ ~ 180° and ψ ~ 90°, 270° which translate into the $\alpha_D^{+/-}$ and $C_{\beta7}^{+/-}$ states within the ϕ/ψ plot (Figure S40). Furthermore, a symmetric λ/ϕ plot can be found which, like the cis case, is superimposable when viewed in the λ/ψ dimension though this is harder to visualise as access to the $C_{\beta7}^{+/-}$ states makes the 3d-dimensional plots more convoluted (Figures S41 and S42). Importantly the accessible λ states have reasonably converged by 50 ns at 298.15 K and on this basis it can be concluded that sampling in this dimension is representative of all accessible states in the multimolecular systems.

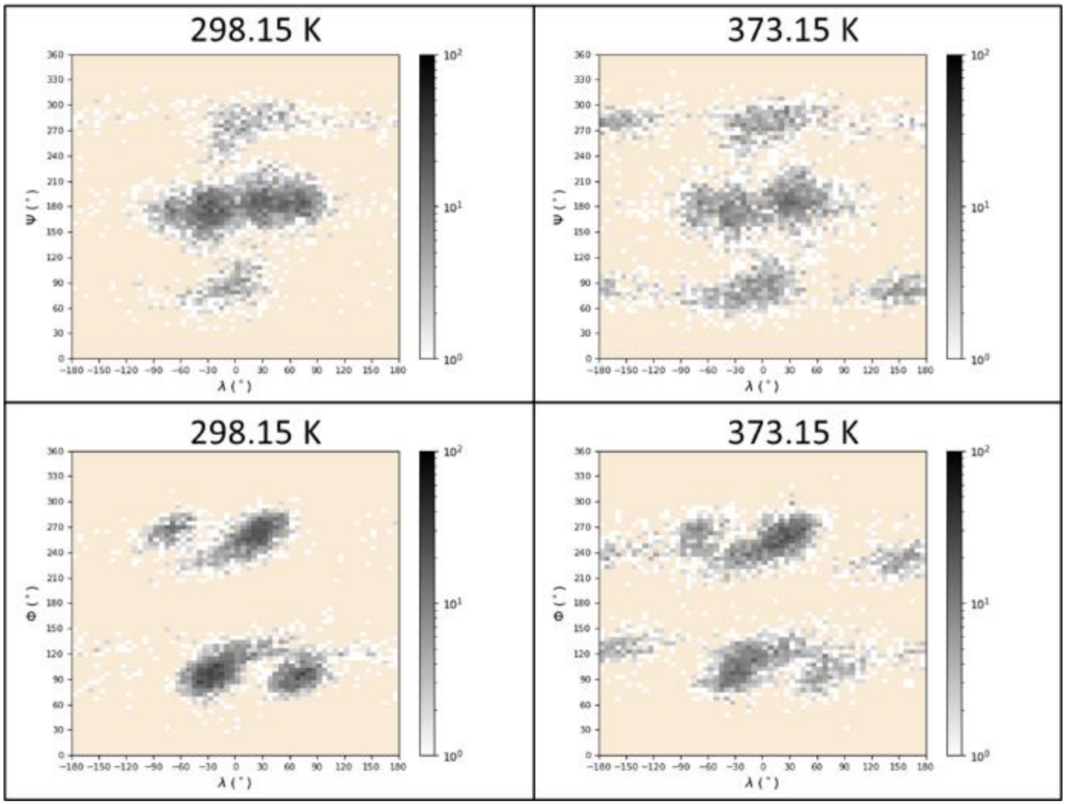

Figure S40. 2D histogram plots of λ/ψ and λ/ϕ for residue 1 in tt Ac-(Nf)$_2$ at 298.15 and 373.15 K in TIP3P water over the duration of 100 ns (bin size = 6°, 4000 frames, 1 molecule). Here again it is possible to see super-position of λ/ϕ states which become indistinguishable in the λ/ϕ dimension as in the *cis* case.

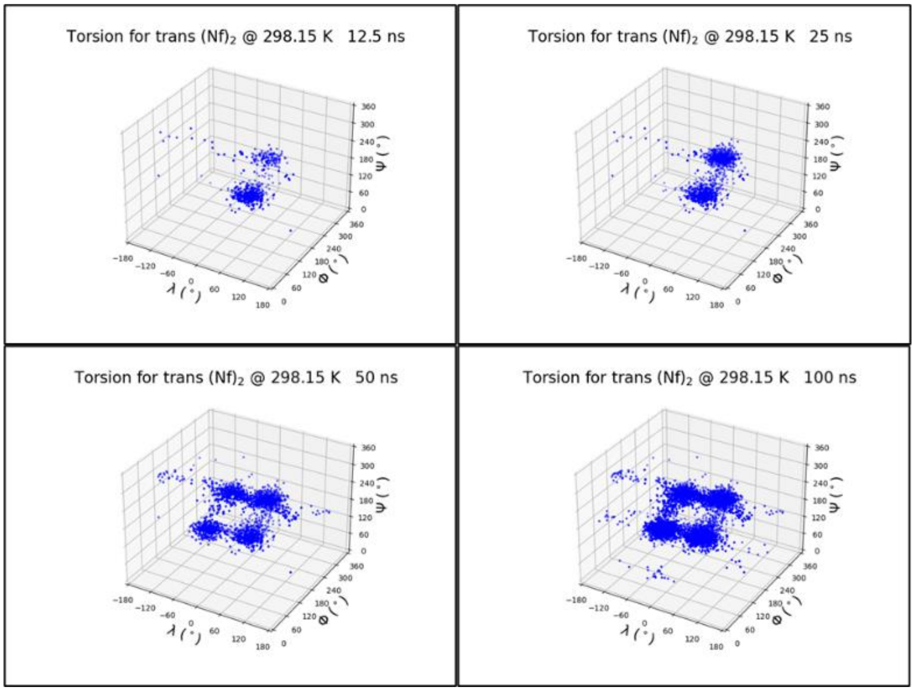

Figure S41. 3D plot of λ/ϕ/ψ states for residue 1 in tt Ac-$(Nf)_2$ at 12.5, 25, 50 and 100 ns at 298.15 K.

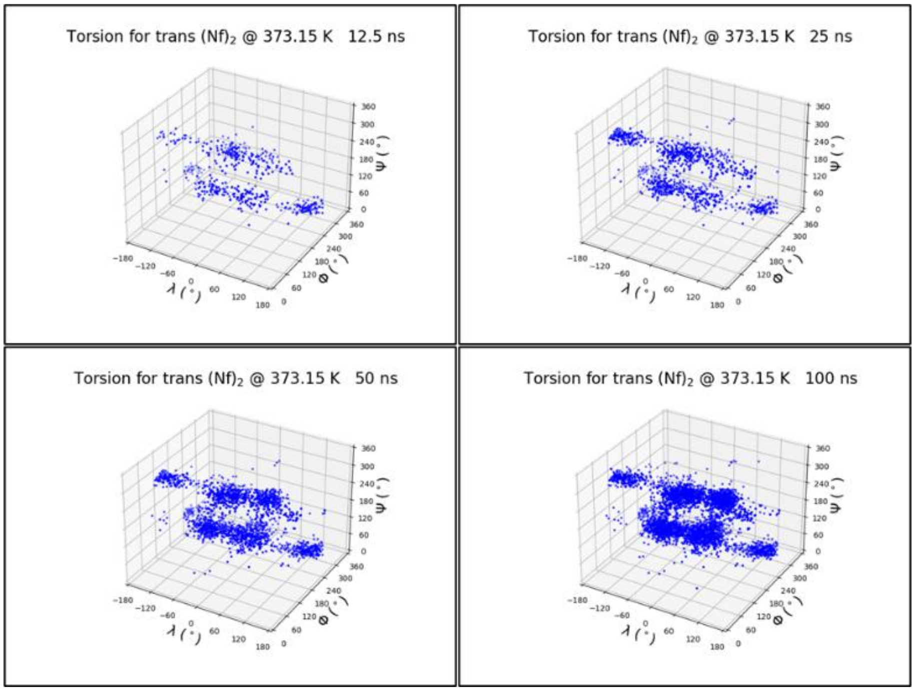

Figure S42. 3D plot of λ/ϕ/ψ states for residue 1 in tt Ac-$(Nf)_2$ at 12.5, 25, 50 and 100 ns at 373.15 K.

2.3.5 **Dimer Ac-(Nfes)$_2$**

The ϕ/ψ sampling preferences for *cis*-1 in Ac-(Nfes)$_2$ is for the α_D minima (Figure S43), though as expected this is biased towards the $\alpha_D$$^-$ minima which corresponds to right handed structrue formation (Table S32) Experimentally this preference is realised in the formation of right-handed polyproline type I (PPI) helices[24] and it is established that a ϕ ≈ 290^o and ψ≈ 180^o gives rise to an ideal right handed helix.[20, 25] Recently DFT and atomistic metadynamics studies have shown that Nfes actually has a slight energetic preference to form left-handed helices,[23] with the $\alpha_D$$^-$ minima being less stable than its $\alpha_D$$^+$ counterpart by -0.41 kcal/mol for the dodecamer Nfes$_{12}$.[21] The sampling observed in our forcefield does not imply this difference, but additional simulations, with enhanced sampling, would be required to interrogate this further. Sampling by the *trans-1* state is predominately focused on both α_D states and also the α$^{+/-}$ states, which through enhanced sampling of dodecamer Nfes$_{12}$ is about 2-3 kcal/mol above α_D (Figure S43). So too are the $C_{7\beta}$ states, however these are not accessed in our simulations; this may be due to the barrier height which precluded exploration of this region of the Ramachandran plot in this simulation and further analysis of will be the foucs of further work.

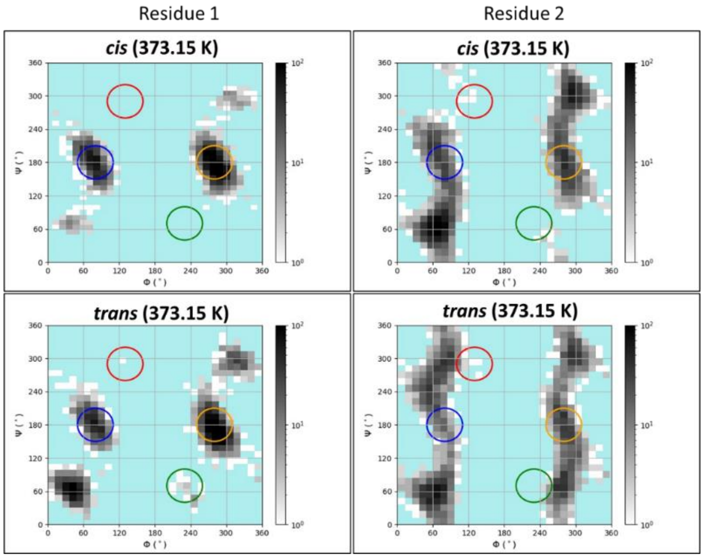

Figure S43. ϕ/ψ sampling for Ac-(Nfes)$_2$ over 100 ns (4000 frames) at 373.15 K to enhance sampling of the surface. Here we find reasonable agreement with expected samplign for residue 1 in both *cis* and *trans* states; however the near absence of the $C_{7\beta}$$^{+/-}$ state and instead sampling of $\alpha$$^{+/-}$ was suprising and further investigation of this PES will be required as further work (number of bins = 30).

Table S32 – Occupancy of $\alpha_D^{+/-}$ and $C_{7\beta}^{+/-}$ for residue one of Ac-(Nfes)$_2$ at 373.15 K.

Temperature	Minima Region	% occupancy *cis*	% occupancy *trans*
**373.15 K**	$\alpha_D{}^+$	22.044	13.297
	$\alpha_D{}^-$	50.137	36.491
	$C_{7\beta}{}^+$	0.0	0.025
	$C_{7\beta}{}^-$	0.0	0.525

2.3.6 Peptoid Helix Simulation Details

A hexameric peptoid (Nk-Nfes-Nfes)$_2$ was solvated with TIP3P water and chloride ions in a 4 x 4 x 4 nm box. This was minimized using the settings outlined in Section 2.3.1. The following treatment was performed, firstly an NVT ensemble was applied to the system with velocity generation; velocity rescaling was to a reference temperature of 298.15 K with a time constant of 1.0 ps (3000000 steps, ts = 1 fs, 3 ns). Following this an NPT ensemble was applied using the same temperature coupling and isotropic Berendsen pressure coupling, with a time constant of 1.0 ps, compressibility of 4.5x10^{-5} bar^{-1}, target pressure of 1.01325 bar (3000000 steps, ts = 2 fs, 6 ns). The production run employed the NPT ensemble with Nose-Hoover temperature coupling, isotropic Parrinello-Rahman pressure coupling for which a time coupling of 2.0 ps was used (50000000 steps, ts = 2 fs, 100 ns).

2.3.7 Helix (Nk-Nfes-Nfes)$_2$ Results

As an additional test of our simulation parameters, we simulated the dynamics of a peptoid helix fragment (Nk-Nfes-Nfes)$_2$ from which antimicrobial peptoids can be derived.[26] The starting structure was based on the PPI type helix, which is stable in the $\alpha_D{}^-$ minima ($\phi \approx 290^o$, $\psi \approx 180^o$ and $\omega \approx 0^o$). Here we found that the sampling of residues 2 – 4 exactly occupy this minima as would be expected for the right handed helix (Figure S44). The additional sampling of $\alpha^{+/-}$ minima for residue 1 can be accounted for in that the *i – 1* residue is an achiral N-terminal Nk residue and so the sequential enforcement Ramachandran preference is only operative from the *i + 1* Nfes residue (residue 2). Meanwhile for residue 5 it is found that sampling of both α_D minima occurs

and we attribute this to the same effect, where no stabilisation of α_D- can occur from the *i + 1* residue as this is the C-terminal chain end. Notably, in the X-ray crsytal structure obtained for a peptoid helix tetramer, Nfes[naph], by Stringer *et al.* found ϕ = 75.7° at the C-terminus.[24]An important qualitative observation from these simulations is the facial aromatic amphiphilicity which arises from this sequence pattern through. Reproducing this *in silico* is an important target feature giving its structural importance to peptoid helices as has been well established by experiment.[27 28 29]

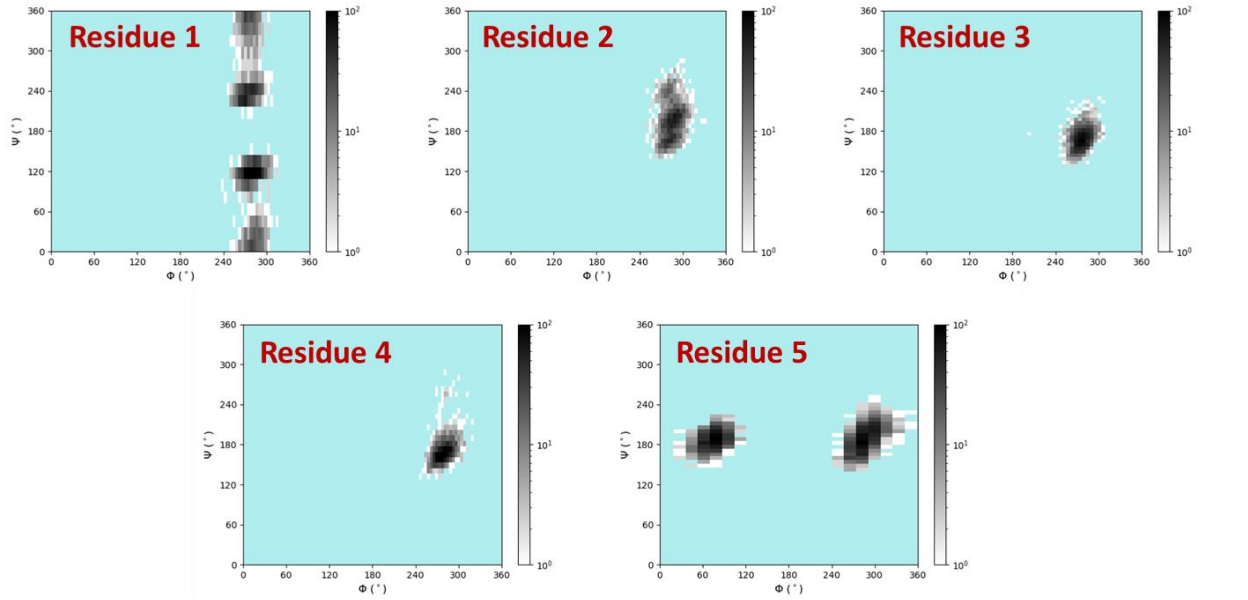

Figure S44. ϕ/ψ sampling for helical peptoid fragement (Nk-Nfes-Nfes)$_2$ on a per torsion pair basis. Sampling of the α_D- region is an indicator that our representation of the Nfes residue, within a helical structure, adopts the correct backbone torsions. The increased degrees of freedom at residue 1 and 5 are thought to be due to the absence of helix enforcing residues at one flank of either torsion. (number of bins = 30).

2.3.8 System Compositions and Concentrations

Solvent Mixture Calculation (Ac-Nf-Nf):

Taking a 1 mL volume of a 70 % acetonitrile: 30 % water solution, with 0.7 mL of acetonitrile (ρ = 0.786 g/mL, mol. wt. = 41.05 g/mol) and 0.3 mL of water (ρ = 0.997 g/mL, mol. wt. = 18.015 g/mol) at standard conditions.

$$n_{H_2O} = \frac{(0.3 \cdot 0.997)}{18.015} = 0.0166 \text{ moles}$$

$$n_{acn} = \frac{(0.7 \cdot 0.786)}{41.05} = 0.0134 \text{ moles}$$

$$\textit{Ratio of moles} = \frac{n_{H_2O}}{n_{acn}} = \frac{0.0166}{0.0134} = \underline{1.239}$$

Of 2500 solvent molecules, 1116 acetonitrile molecules and 1384 water molecules are used:

$$n_{H_2O} = 6.02214076 \text{x} 10^{23} \cdot 1384 = 2.29819 \text{ x} 10^{-21}$$

$$n_{acn} = 6.02214076 \text{x} 10^{23} \cdot 1116 = 1.85316 \text{ x} 10^{-21}$$

$$\textit{Ratio of moles} = \frac{n_{H_2O}}{n_{acn}} = \frac{2.29819 \text{ x} 10-21}{1.85316 \text{ x} 10-21} = \underline{1.240}$$

System Concentration Calculation:

Box volume = 4 x 4 x 4 nm = 64 nm^3 → 6.4 x 10^{-23} L

Moles Molecule = 25/6.022 x 10^{-23} = 4.151 x 10^{-23} mol

Concentration = 4.151 x 10^{-23}/6.4 x 10^{-23} = 0.648 M

3. Additional ρ vs. λ Data

3.1 ρ vs. λ for all amide conformations

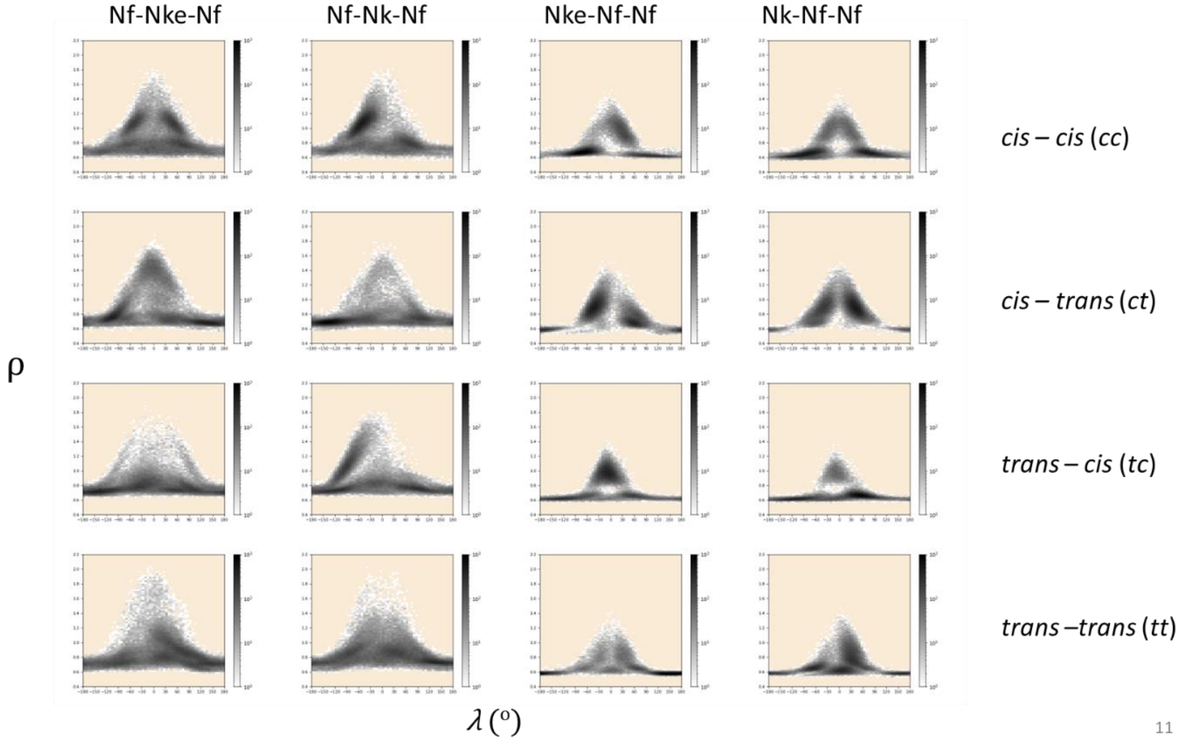

Figure S45. ρ vs. λ plots for all amide conformations (*cis – cis, cis – trans, trans – cis* and *trans – trans*) for tripeptoids Nf-Nke-Nf, Nf-Nk-Nf, Nke-Nf-Nf and Nk-Nf-N data set 1.

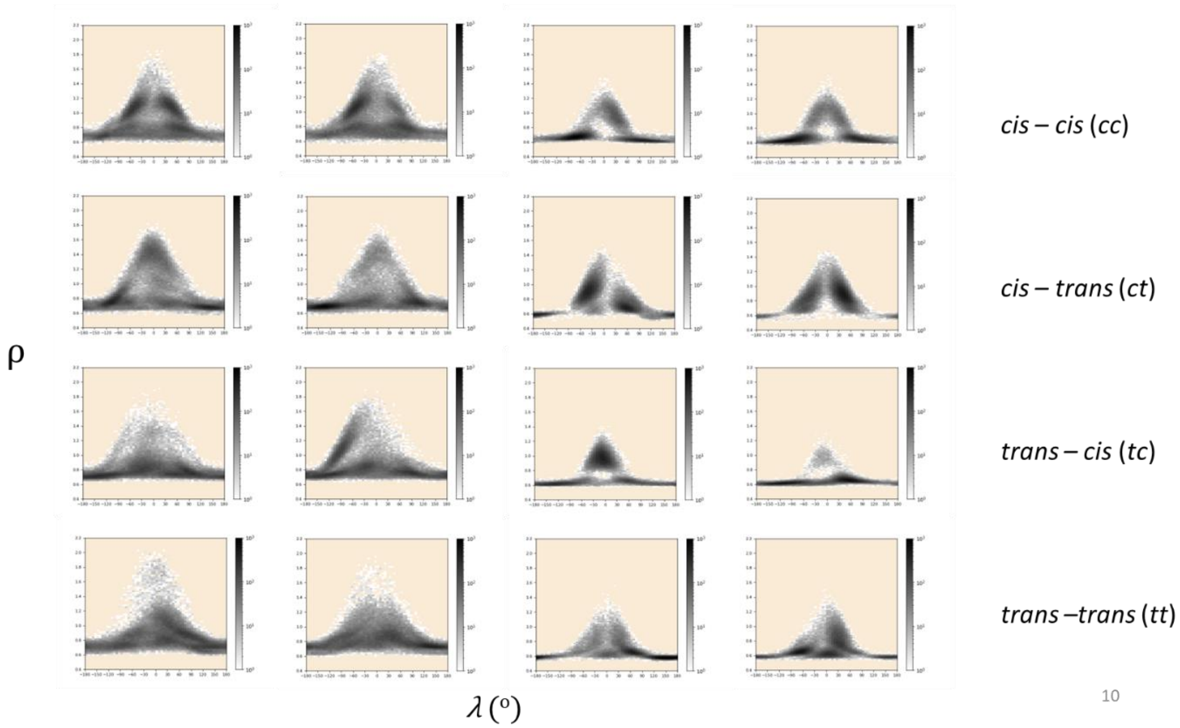

Figure S46. ρ vs. λ plots for all amide conformations (cis – cis, cis – trans, trans – cis and trans – trans) for tripeptoids Nf-Nke-Nf, Nf-Nk-Nf, Nke-Nf-Nf and Nk-Nf-Nf data *s*et 2.

3.2 ρ vs. λ for duplicate peptide simulations

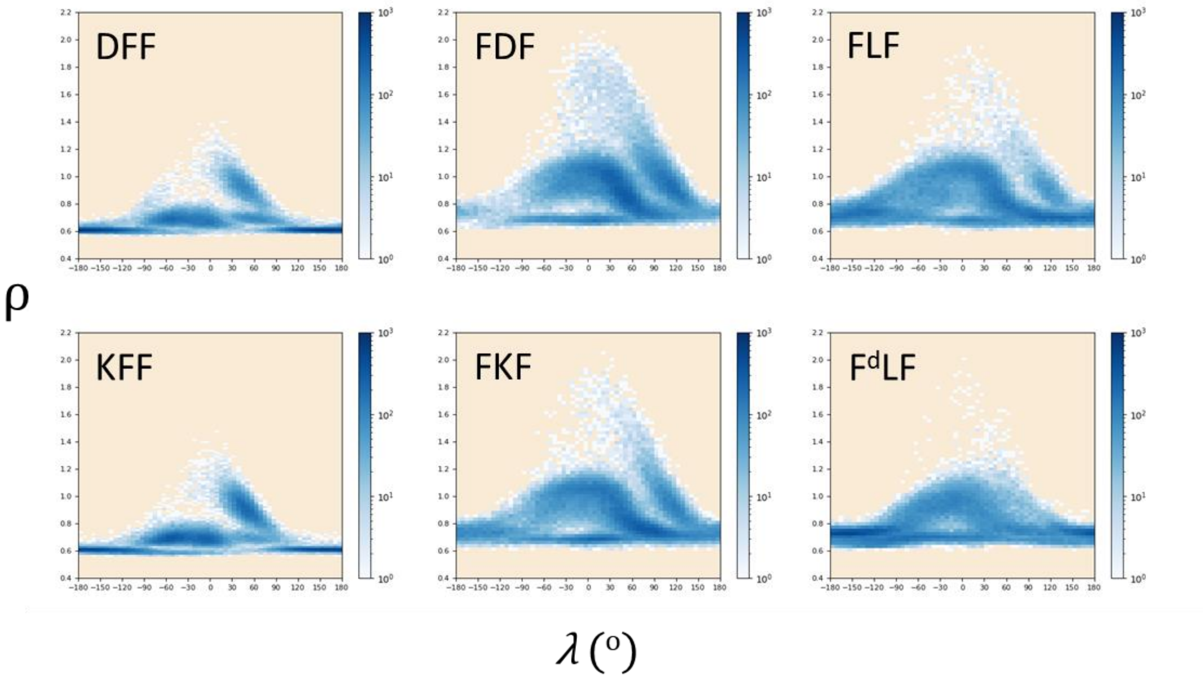

Figure S47. ρ vs. λ plots for all tripeptides in this study, derived from repetition of simulation using the same starting structure as in Figure 4 from the main manuscript. For FLF and F^dLF a close relation between phenyl sidechain dynamics is observed despite differences in backbone chirality; these results agree with the AME evaluations which indicate that intermolecular interactions may be of greater importance to differences in assembly propensity than intramolecular contacts.

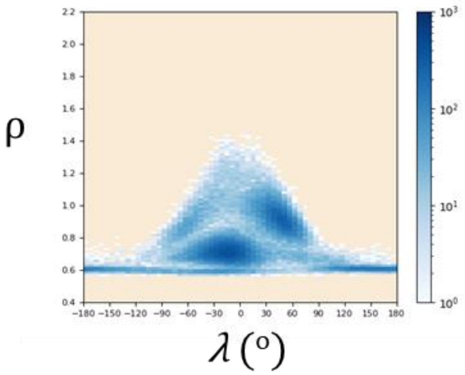

Figure S48. ρ vs. λ plots for all dipeptide FF using same starting structure as data set for which the ρ vs. λ plots was generated in Figure 3.

3.3 Validation of averaging $\overline{x}_{\lambda}$ across all amide states

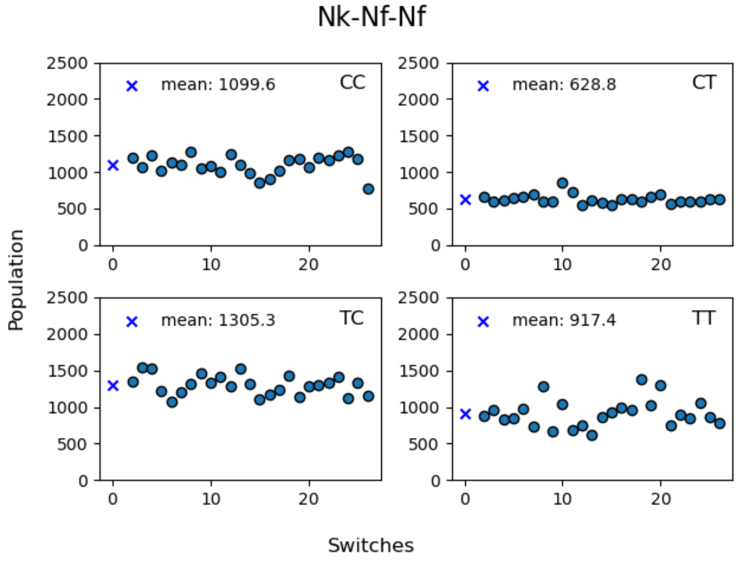

Figure S49. $\sum|\Delta\lambda| > 20^{o}$ for tripeptoid Ac-Nf-Nf for each amide state combination showing these to be consistent and thus making it reasonable to represent them as an average property (Data Set 1).

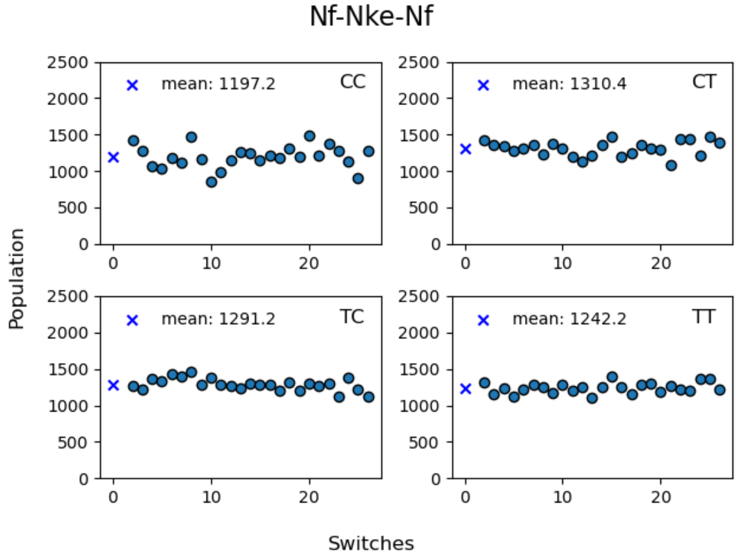

Figure S50. $\sum|\Delta\lambda| > 20^{o}$ for tripeptoid Nf-Nke-Nf for each amide state combination showing these to be consistent and thus making it reasonable to represent them as an average property (Data Set 2).

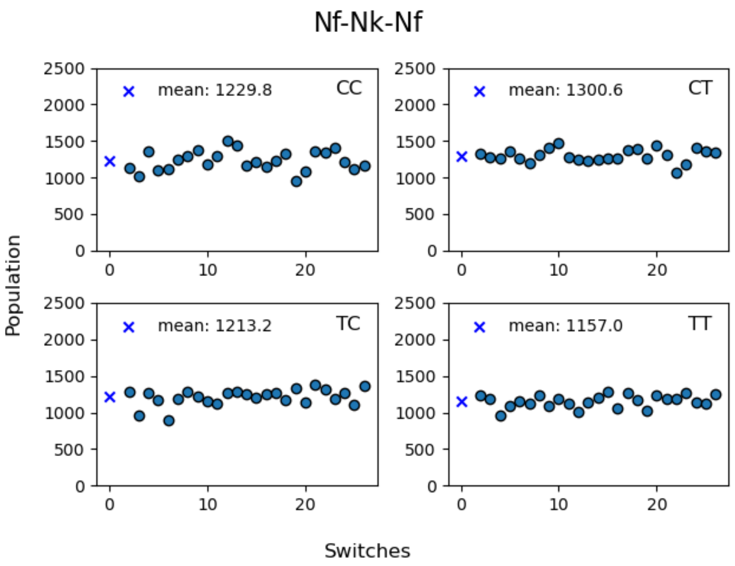

Figure S51. $\sum|\Delta\lambda| > 20^o$ for tripeptoid Nf-Nk-Nf for each amide state combination showing these to be consistent and thus making it reasonable to represent them as an average property (Data Set 2).

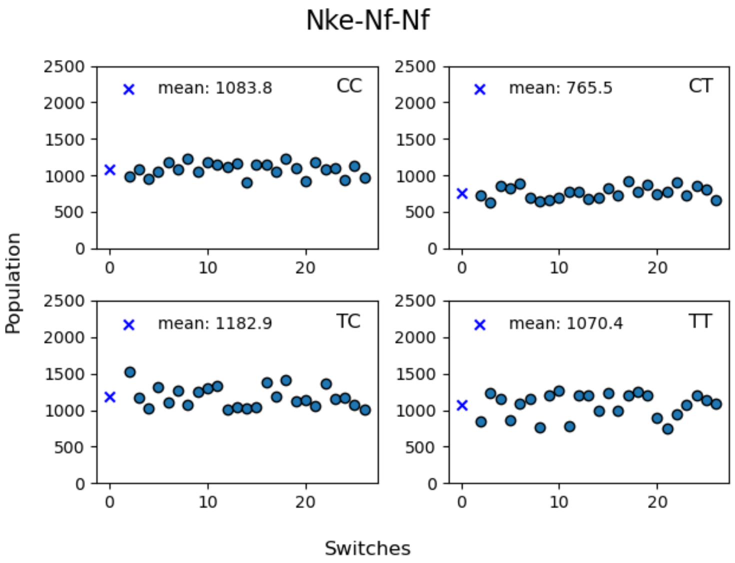

Figure S52. $\sum|\Delta\lambda| > 20^o$ for tripeptoid Nke-Nf-Nf for each amide state combination showing these to be consistent and thus making it reasonable to represent them as an average property (Data Set 2).

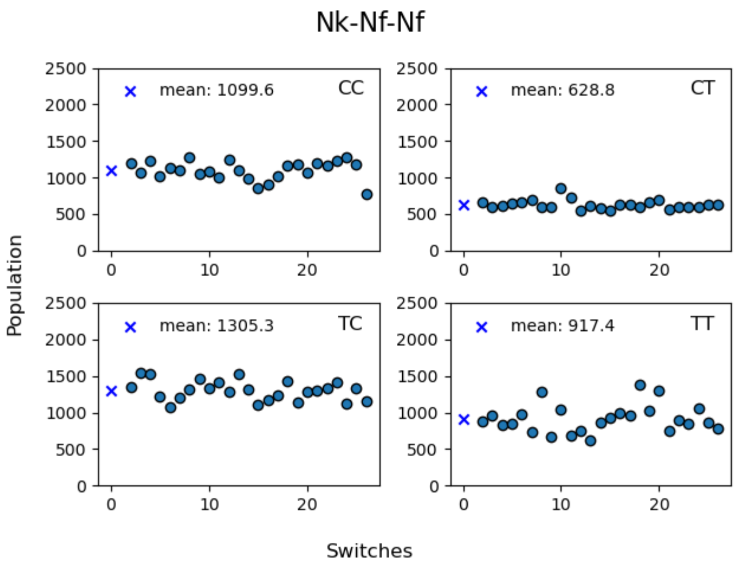

Figure S53. $\sum|\Delta\lambda| > 20^o$ for tripeptoid Nk-Nf-Nf for each amide state combination showing these to be consistent and thus making it reasonable to represent them as an average property (Data Set 2).

To assess the extent to which variation in $\bar{x}_\lambda$ effects η we performed bootstrapping analysis to generate mean $\bar{x}_\lambda$values using replacement (n = 1000). It was found that the bootstrapped means for a given dataset conformed to a narrow and generally normal distribution (Tables S33 and S34), this is reflected by the fact that the 95% confidence interval is narrow and is illustrated pictorially in Figures S54 – S58. Further the associated mean standard deviation was small and the 95 % confidence interval being generally narrow. On this basis it can be concluded that the estimated η is insensitive to molecule choice. Additionally, we estimated η with the bootstrapped means and found good agreement with the original values and trend reported.

Table S33 – Bootstrapping statistics obtained for Data Set 1 where n = 1000 for each array.

Species	State	$\overline{x}_{\lambda}$ bootstrap	$\overline{\sigma}_{\lambda}$ bootstrap	95 % Confidence interval $\overline{x}_{\lambda}$		95 % Confidence interval $\overline{\sigma}_{\lambda}$		η
Ac-Nf-Nf	cc	1299.9	89.8	1266.0	1333.0	67.0	114.2	1.7
	ct	845.9	55.7	824.1	869.5	32.6	75.0	
	tc	1474.6	137.1	1418.4	1529.2	110.0	160.5	
	tt	1228.3	83.3	1192.6	1261.0	57.4	105.7	
Nk-Nf-Nf	cc	1045.3	111.7	1001.1	1089.9	90.5	132.4	1.9
	ct	610.1	73.6	581.5	642.6	44.9	102.1	
	tc	1187.9	166.4	1119.0	1255.5	129.3	200.4	
	tt	903.9	135.6	849.2	958.8	93.8	175.6	
Nke-Nf-Nf	cc	1020.5	87.1	986.5	1054.8	62.1	112.0	1.6
	ct	733.7	71.6	706.1	762.6	52.4	89.8	
	tc	1169.9	114.7	1126.6	1218.6	74.9	149.8	
	tt	1116.7	175.1	1044.4	1182.5	107.1	226.5	
Nf-Nk-Nf	cc	1123.7	145.4	1069.2	1183.0	110.7	178.2	1.2
	ct	1315.4	79.1	1280.1	1347.9	50.9	103.7	
	tc	1194.2	119.1	1144.6	1237.4	70.4	163.6	
	tt	1170.0	65.3	1145.6	1196.7	50.5	78.5	
Nf-Nke-Nf	cc	1258.1	131.0	1203.3	1310.9	93.4	163.9	1.0
	ct	1292.0	96.1	1248.3	1328.4	62.2	124.7	
	tc	1306.1	62.6	1279.8	1329.9	42.2	84.0	
	tt	1244.3	47.7	1226.4	1263.6	36.4	58.2	

Table S34 – Bootstrapping statistics obtained for Data set 2 where n = 1000 for each array.

Species	State	$\bar{x}_{\lambda}$ bootstrap	$\bar{\sigma}_{\lambda}$ bootstrap	95 % Confidence interval $\bar{x}_{\lambda}$		95 % Confidence interval $\bar{\sigma}_{\lambda}$		η
Nk-Nf-Nf	cc	1099.1	121.8	1045.8	1146.5	84.5	157.3	2.1
	ct	628.7	58.8	605.4	654.0	33.5	87.7	
	tc	1305.6	128.3	1256.2	1358.3	97.1	154.3	
	tt	917.2	183.3	851.0	992.1	122.3	235.3	
Nke-Nf-Nf	cc	1084.3	90.2	1048.1	1119.3	68.8	108.7	1.5
	ct	765.0	83.1	733.8	798.5	66.9	99.5	
	tc	1183.3	137.1	1132.8	1245.2	103.4	171.4	
	tt	1071.1	161.9	1007.1	1136.2	123.7	191.5	
Nf-Nk-Nf	cc	1230.3	129.7	1180.6	1281.6	98.6	158.3	1.1
	ct	1300.0	86.4	1262.8	1330.2	62.9	111.6	
	tc	1214.3	106.9	1169.8	1253.5	64.3	147.6	
	tt	1156.7	81.9	1123.3	1186.8	58.0	102.5	
Nf-Nke-Nf	cc	1198.6	149.4	1139.8	1256.1	105.1	187.2	1.1
	ct	1309.3	100.0	1269.5	1346.8	76.1	123.2	
	tc	1292.2	80.5	1260.2	1324.1	56.9	102.6	
	tt	1242.9	69.4	1216.6	1269.6	50.6	88.1	

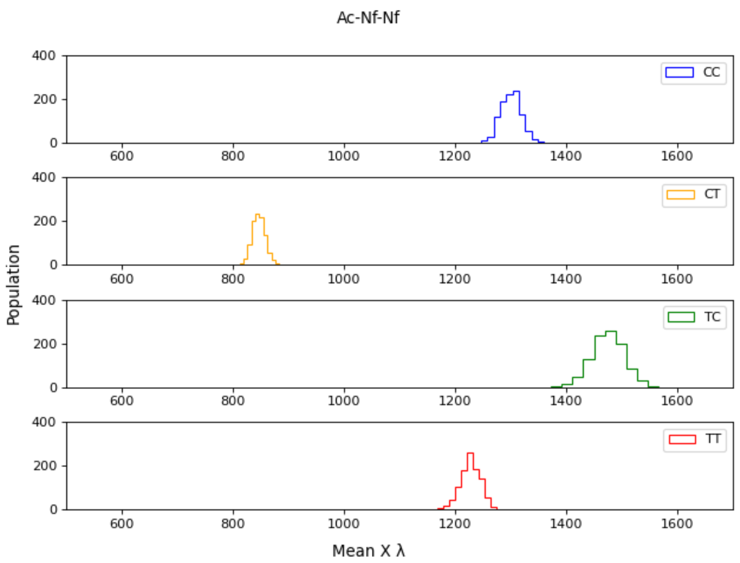

Figure S54. Bootstrapped mean $\bar{x}_\lambda$ values (n = 1000) for Ac-Nf-Nf across amide sequences cc, ct, tc and tt showing that these are normally distributed and thus confirming that $\bar{x}_\lambda$ is not affected by specific molecules within the simulation set (n = 25).

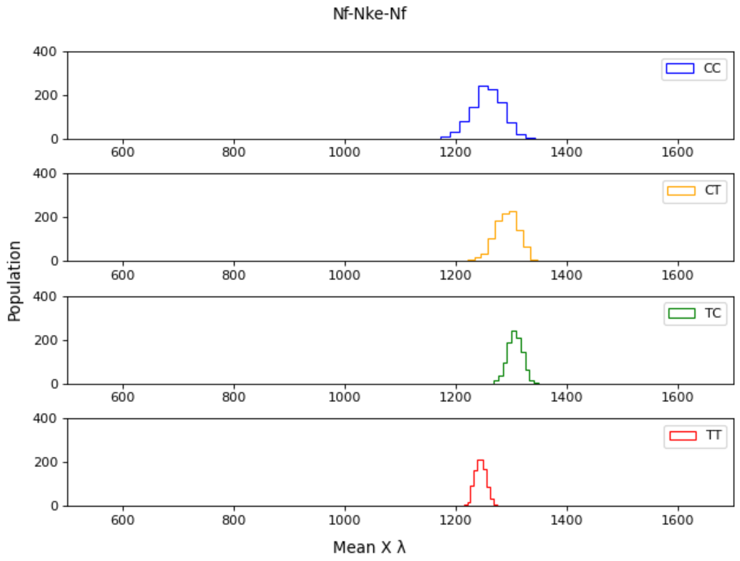

Figure S55. Bootstrapped mean $\bar{x}_\lambda$ values (n = 1000) for Nf-Nke-Nf across amide sequences cc, ct, tc and tt showing that these are normally distributed and thus confirming that $\bar{x}_\lambda$ is not affected by specific molecules within the simulation set (n = 25).

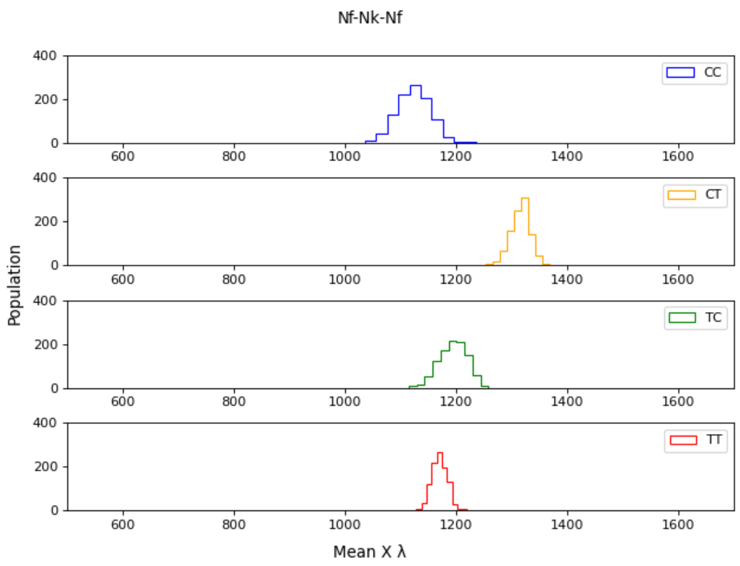

Figure S56. Bootstrapped mean $\bar{x}_\lambda$ values (n = 1000) for Nf-Nk-Nf across amide sequences cc, ct, tc and tt showing that these are normally distributed and thus confirming that $\bar{x}_\lambda$ is not affected by specific molecules within the simulation set (n = 25).

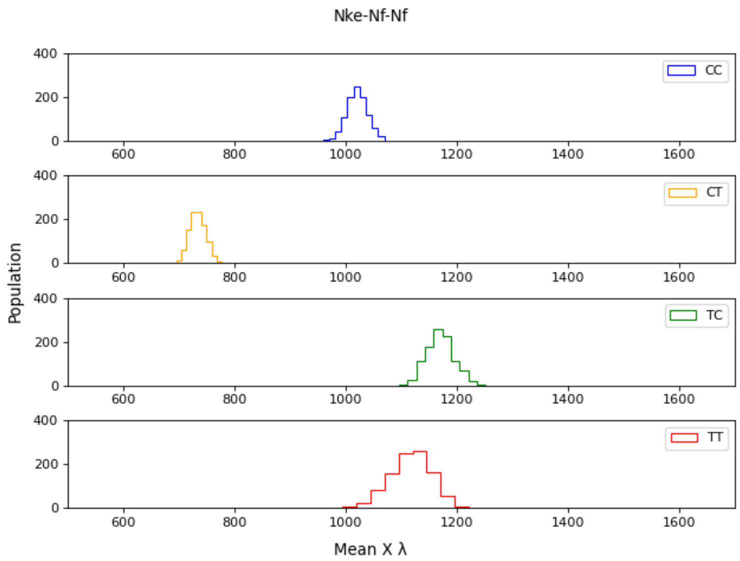

Figure S57 Bootstrapped mean $\bar{x}_\lambda$ values (n = 1000) for Nke-Nf-Nf across amide sequences cc, ct, tc and tt showing that these are normally distributed and thus confirming that $\bar{x}_\lambda$ is not affected by specific molecules within the simulation set (n = 25).

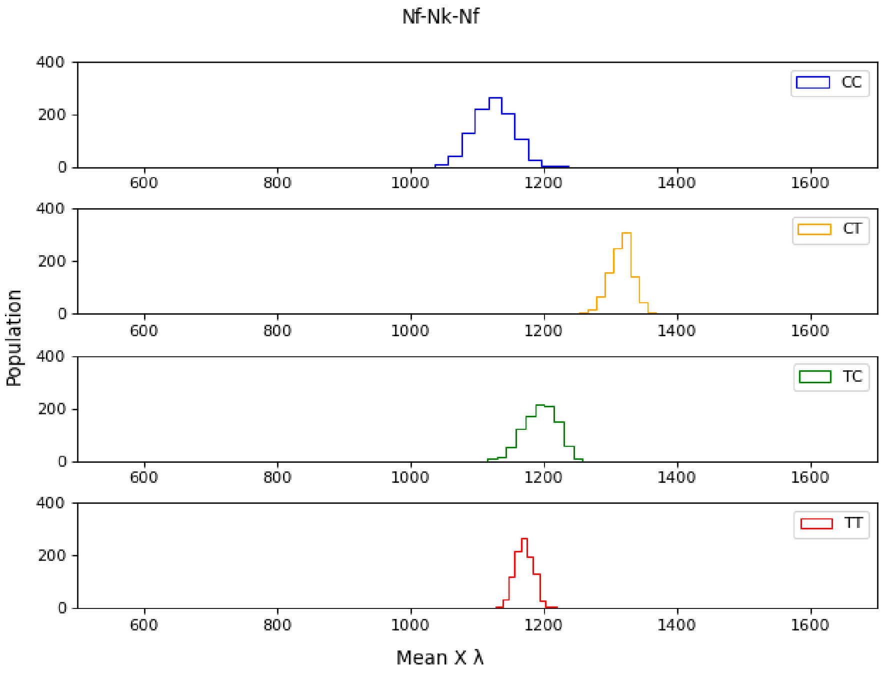

Figure S58. Bootstrapped mean $\bar{x}_\lambda$ values (n = 1000) for Nk-Nf-Nf across amide sequences cc, ct, tc and tt showing that these are normally distributed and thus confirming that $\bar{x}_\lambda$ is not affected by specific molecules within the simulation set (n = 25).

3.4 Validation of 50 ns simulation time

The use of 50 ns simulation time is partly grounded on the basis that by using multiple molecules we can effectively access 1250 ns of sequence simulation time (as 25 x 50 ns). A key metric in this study, η, is dependent on sampling of the λ dimension. A 'switch' in this variable is counted when $|\Delta\lambda| > 20^o$ and so we measured the cumulative increase in Δλ hits verses time for the Ac-$(Nf)_2$ dimer in both the *cc* and *tt* state over 100 ns in TIP3P in 298.15 K. It was found that this property grew linearly with time, for both amide conformer states, in a manner which is well captured with a linear equation. Given this condition it is possible to cut-off sampling at 50 ns and still obtain the same ratio of cumulative switches as would be obtained at 100 ns. As η depends on the *relative* ratios of sampling in λ between sequences than the preservation of the ratio itself is critical for this property to be meaningful. On this basis we believe 50 ns to be a reasonable simulation time.

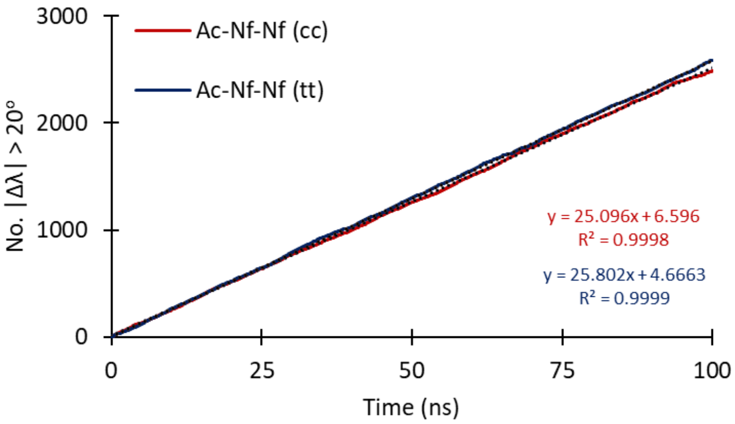

Figure S59. Cumulative No. of |Δλ| > 20° verses time for Ac-Nf-Nf dimer in *cc* and *tt* state.

4. Absolute Molecular Entropy

Table S35 – XFF tripeptides, masses in g mol^{-1} and entropy in cal mol^{-1} K^{-1}.

Mass	Name	SmsRHHO	Sconf	δrhho	Stotal	Assembler?
**369.4**	GFF	183.66	7.98	-2.80	188.83	n
**383.5**	AFF	179.33	4.44	6.62	190.39	n
**399.5**	SFF	190.70	6.75	-3.81	193.63	n
**409.5**	PFF	182.76	4.67	8.45	195.88	y
**411.5**	VFF	190.24	8.56	5.42	204.22	n
**413.5**	TFF	197.12	6.34	-0.78	202.68	n
**415.5**	CFF	192.07	3.53	4.10	199.70	y
**425.5**	LFF	198.72	9.72	5.11	213.55	y
**425.5**	IFF	196.30	10.49	5.33	212.12	y
**426.5**	NFF	199.10	8.45	5.78	213.33	n
**426.5**	DFF	190.22	4.70	1.80	196.72	y
**440.5**	QFF	204.05	9.27	0.05	213.36	n
**441.6**	KFF	208.50	10.78	-7.84	211.44	y
**440.5**	EFF	202.45	10.26	-0.95	211.77	n
**443.6**	MFF	204.82	8.47	4.58	217.87	n
**450.5**	HFF	202.56	9.30	-3.50	208.36	n
**459.6**	FFF	205.55	8.42	1.90	215.87	y
**469.6**	RFF	217.12	12.42	-1.97	227.58	n
**475.5**	YFF	207.85	8.57	-0.46	215.96	n
**498.6**	WFF	216.39	6.31	-2.79	219.91	n

Table S36 – FXF tripeptides, masses in g mol^{-1} and entropy in cal mol^{-1} K^{-1}.

Mass	Name	SmsRHHO	Sconf	δrhho	Stotal	Assembler?
**369.4**	FGF	170.72	6.00	1.35	178.07	n
**383.5**	FAF	187.69	5.63	-3.12	190.19	n
**399.5**	FSF	187.91	6.91	-2.18	192.64	n
**409.5**	FPF	192.05	8.34	-2.94	197.45	n
**411.5**	FVF	194.94	8.26	2.86	206.06	n
**413.5**	FTF	194.63	6.09	-0.39	200.33	y
**415.5**	FCF	195.03	9.18	-3.59	200.62	y
**425.5**	FLF	207.30	9.13	-1.75	214.68	n
**425.5**	FIF	206.09	9.74	4.50	220.34	n
**426.5**	FNF	197.40	7.30	-1.08	203.62	n
**426.5**	FDF	191.47	4.73	1.03	197.23	y
**440.5**	FQF	208.20	8.42	-3.47	213.15	n
**441.6**	FKF	202.77	7.45	-1.05	209.18	y
**440.5**	FEF	202.41	9.18	0.63	212.22	y
**443.6**	FMF	206.00	9.37	2.44	217.81	n
**450.5**	FHF	204.42	6.14	-5.80	204.76	n
**459.6**	FFF	205.55	8.42	1.90	215.87	y
**469.6**	FRF	214.83	10.05	-1.58	223.30	n
**475.5**	FYF	210.17	7.89	4.55	222.61	n
**498.6**	FWF	213.26	6.04	6.24	225.54	n

Table S37 – Tripeptoids, masses in g mol^{-1} and entropy in cal mol^{-1} K^{-1}.

Mass	Name	SmsRHHO	Sconf	δrhho	Stotal	Assembler?
**441.6**	Nk-Nf-Nf	202.12	9.74	5.53	217.39	y
**413.5**	Nke-Nf-Nf	189.37	9.41	5.95	204.72	y
**441.6**	Nf-Nk-Nf	201.49	14.65	6.68	222.82	y
**413.5**	Nf-Nke-Nf	192.05	9.65	2.22	203.92	y

Table S38 – Heterochiral Peptides, masses in g mol^{-1} and entropy in cal mol^{-1} K^{-1}.

Mass	Name	SmsRHHO	Sconf	δrhho	Stotal	Assembler?
**411.5**	^{d}VFF	190.70	9.26	3.71	203.67	y
**425.5**	^{d}LFF	204.10	8.09	-1.85	210.35	y
**425.5**	F^{d}LF	205.04	9.34	-0.37	214.01	y
**425.5**	F^{d}IF	209.43	8.95	-5.95	212.43	y

Table S39 – Method Verification, masses in g mol^{-1} and entropy in cal mol^{-1} K^{-1}.

Name	SmsRHHO	Sconf	δrhho	Stotal	Pracht *et al.*[30]
***n*-butane**	72.39	2.04	0.02	74.45	74.21
***n*-pentane**	80.19	3.13	0.88	84.20	83.55
***n*-hexane**	88.01	5.12	0.82	93.95	92.94
***n*-decane**	118.82	13.4	0.67	132.89	130.50
**Tamiflu**	178.46	13.7	1.74	193.9	189.4

5. B97-3c Optimised Energies

Five structures were selected entirely at random over the 25 Ac-Nf-Nf molecules and 2500 frames produced for the 50 ns simulation. `numpy.random.int()` was used to generate an array of (5,2) molecules and frames. A different random seed was used for each amide sequence combination. The measured torsions ϕ, ψ, ω and χ (as $C - N - C_{\beta} - C_{\gamma}$) and energy values were collated and are reproduced here in Table S40. Additionally, the same torsions at the CGenFF level of theory are reproduced in Table S41. Generally, all torsions are in agreement with expected torsions from experimental structures and other computational studies.[21, 31] The measured energies occupy a narrow range of < 4 kcal/mol, which confirms the intuitive notion that an ensemble of backbone conformations will exist in solution.

Table S40 – All relevant torsions and energies for 5 randomly selected structures for Ac-$(Nf)_2$ in the cc, ct, tc and tt omega states at the B97-3c level of theory.

**Level of Theory: B97-3c**	**Structure**	**ω1**	**ω2**	**ϕ1**	**ϕ2**	**ψ1**	**ψ2**	**χ1**	**χ2**	Energy (Ha)	Normalised (kcal/mol)
cc	1	-5.0	-6.2	-87.0	-86.8	-174.5	-160.5	-120.3	-116.6	-1165.45707	-1.418
	2	-4.5	-3.2	-89.7	89.2	-167.0	2.8	-121.9	103.8	-1165.45835	-2.216
	3	-7.5	-7.8	147.3	-88.4	-61.0	-156.1	105.3	-117.3	-1165.45491	-0.057
	4	-4.7	0.3	-91.5	78.1	-165.8	11.7	-121.2	-105.5	-1165.45852	-2.326
	5	-4.9	-0.2	-83.4	-80.0	178.9	-4.9	-123.1	104.9	-1165.45775	-1.839
ct	1	-4.7	179.3	-87.1	88.5	-168.4	-68.1	-119.0	78.9	-1165.45834	-2.212
	2	-5.1	179.7	-85.8	91.0	-170.5	-0.9	-118.9	76.6	-1165.46023	-3.397
	3	-4.2	179.2	-87.7	88.6	-167.5	-65.4	-117.8	80.0	-1165.45842	-2.262
	4	-4.6	-176.9	-87.2	87.0	-165.9	178.8	-117.9	81.5	-1165.4593	-2.815
	5	-4.9	178.7	-86.1	91.1	-169.2	-0.3	-117.7	77.4	-1165.46023	-3.397
tc	1	179.5	5.4	87.6	88.1	179.8	163.9	112.3	115.5	-1165.45669	-1.177
	2	179.7	-6.4	122.0	83.1	133.0	5.6	128.2	97.4	-1165.45759	-1.740
	3	-176.7	5.0	-137.6	81.3	65.8	-0.6	-104.0	-106.6	-1165.45514	-0.202
	4	179.7	-3.9	-94.6	90.8	-167.1	0.0	-122.2	107.3	-1165.45842	-2.265
	5	-179.7	-6.9	90.8	-83.8	174.5	-153.0	115.8	106.8	-1165.45737	-1.605
tt	1	-177.3	-176.9	-133.1	87.4	66.4	-73.2	-109.6	117.4	-1165.45485	-0.019
	2	-177.0	-177.0	-133.9	87.3	66.5	-74.8	-109.2	115.7	-1165.45482	0.000
	3	-179.8	173.0	-86.7	89.7	178.7	-67.4	-117.6	-69.9	-1165.45568	-0.545
	4	-176.7	-177.8	-132.5	87.5	67.5	-73.9	-109.6	117.4	-1165.45483	-0.012
	5	179.8	179.8	-93.1	88.8	-167.2	-68.2	-117.5	78.6	-1165.45817	-2.105

Table S41 – All relevant torsions for 5 randomly selected structures for Ac-$(Nf)_2$ in the cc, ct, tc and tt omega states at the CGenFF-WS level of theory.

Level of Theory: CGenFF-WS	Structure	ω1	ω2	ϕ1	ϕ2	ψ1	ψ2	χ1	χ2
cc	1	-14.3	-0.4	-71.9	-87.9	151.0	-90.0	-165.6	-85.9
	2	-1.3	17.4	-108.4	81.6	166.6	16.6	-74.3	114
	3	2.5	-14.3	139.4	-79.1	-63.0	-96.6	89.0	-83.1
	4	6.4	-2.3	-99.6	66.3	-157.9	68.6	-102.3	-90
	5	14.8	1.8	-102.8	-70.1	166.7	-52.2	-86.9	86.7
ct	1	2.7	166.7	-85.1	101.9	-174.0	-61.2	-89.6	61.9
	2	4.0	-171.0	-84.4	85.4	-157.7	71.2	-98.0	55
	3	-10.8	179.2	-92.2	92.9	-165.1	-76.0	-104.2	76
	4	-3.7	-165.3	-88.3	98.1	-162.9	-178.8	-88.1	54.7
	5	13.1	170.5	-108.7	128.3	-179.2	-72.5	-80.1	91
tc	1	-169.2	7.8	96.0	75.7	-178.2	86.7	88.6	94.1
	2	-167.8	5.8	98.9	69.2	171.4	53.1	91.6	83.6
	3	173.3	-6.1	-116.0	119.8	88.6	-61.7	-77.4	-79.9
	4	-163.2	4.6	-86.8	90.1	106.0	3.3	-103.0	93.3
	5	168.0	-5.6	120.9	-76.7	-87.4	-81.2	97.9	70.2
tt	1	167.6	166.7	-113.3	100.9	93.5	-64.4	-73.0	76.1
	2	-170.5	-166.4	-133.2	95.3	80.4	-69.6	-90.3	90.2
	3	-170.6	166.5	-117.8	88.6	176.8	-80.3	-81.3	-51.9
	4	177.0	-173.3	-115.7	72.1	83.1	-78.5	-102.4	86.8
	5	174.2	175.6	-108.2	90.2	102.3	-72.1	-78.5	77.7

6. Ac-Nf-Nf Crystal Structure Data

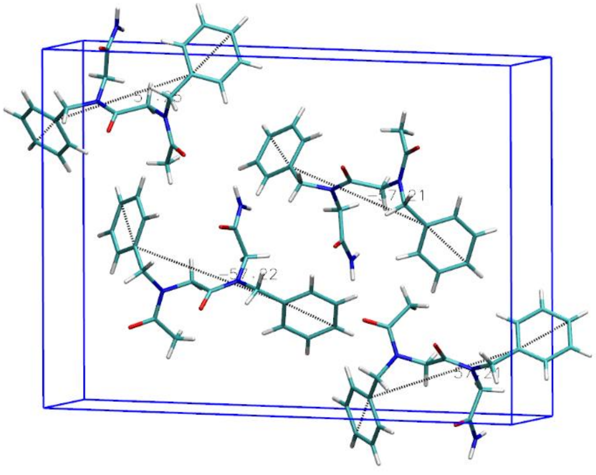

Figure S60. Crystal Structure of minimal peptoid Ac-Nf-Nf (from ref 32) showing the adoption of the ω ~ 0° backbone *cc* state in the crystalline form; consequent lateral arrangement of benzene rings which is unlike that of the assembled dipeptide FF crystal structure reported by Grobitz.[33]

Table S42 – ρ vs. λ data for dipeptoids Ac-Nf-Nf in the crystalline state.

Molecule	λ (°)	CG – CG (Å)	CZ – CZ (Å)	ρ
Top Left	57.250	7.675	11.794	0.651
Bottom Left	-57.220	7.675	11.793	0.651
Top Right	57.210	7.674	11.794	0.651
Bottom Right	57.210	7.675	11.794	0.651

7. ANI-1ccx Gas Phase ρ vs. λ Data

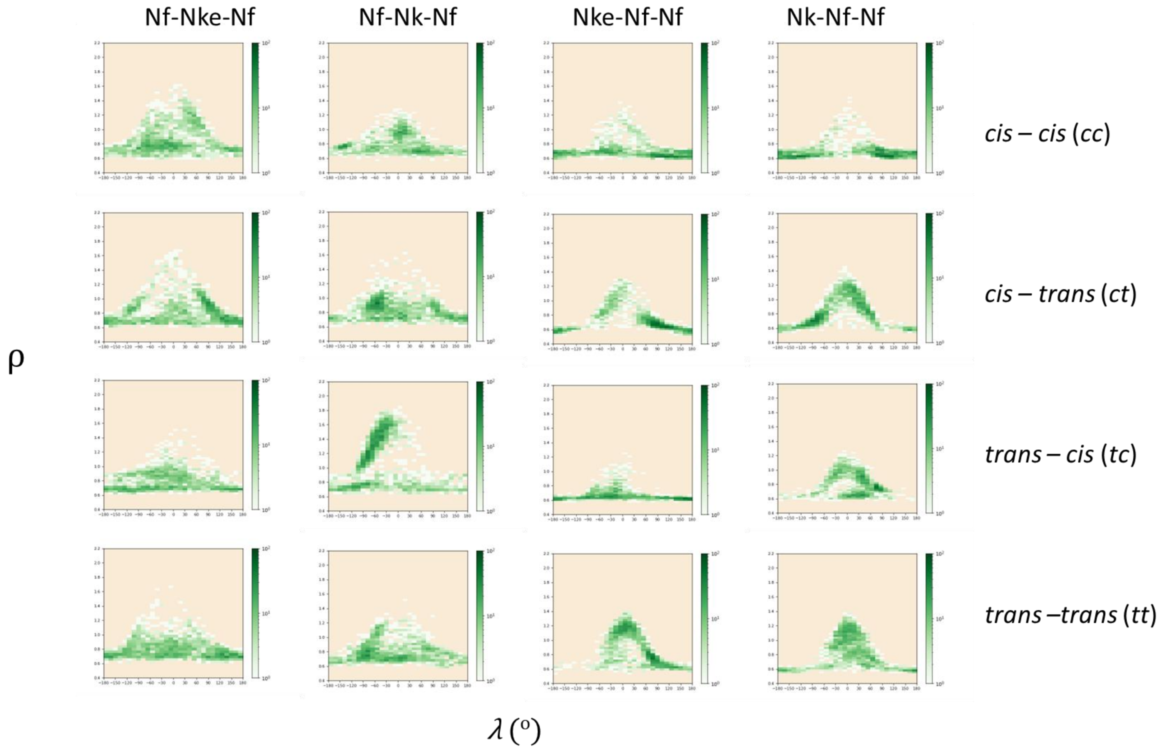

Figure S61. ρ vs. λ plots for all amide conformations (cis – cis, cis – trans, trans – cis and trans – trans) for tripeptoids Nf-Nke-Nf, Nf-Nk-Nf, Nke-Nf-Nf and Nk-Nf-Nf as obtained for a single molecule in the gas-phase using the ANI-1ccx potential (number of bins = 30).

Table S43 – $\bar{x}_{\lambda}$ and η for ANI-1ccx Gas Phase Molecular Dynamics Calculations.

ANI-1ccx	CC	CT	TC	TT	η
Ac-Nf-Nf	901	749	998	947	1.33
Nk-Nf-Nf	1030	668	760	895	1.54
Nke-Nf-Nf	1125	786	1136	792	1.45
Nf-Nk-Nf	1013	971	1022	975	1.05
Nf-Nke-Nf	1069	1037	1328	1060	1.28

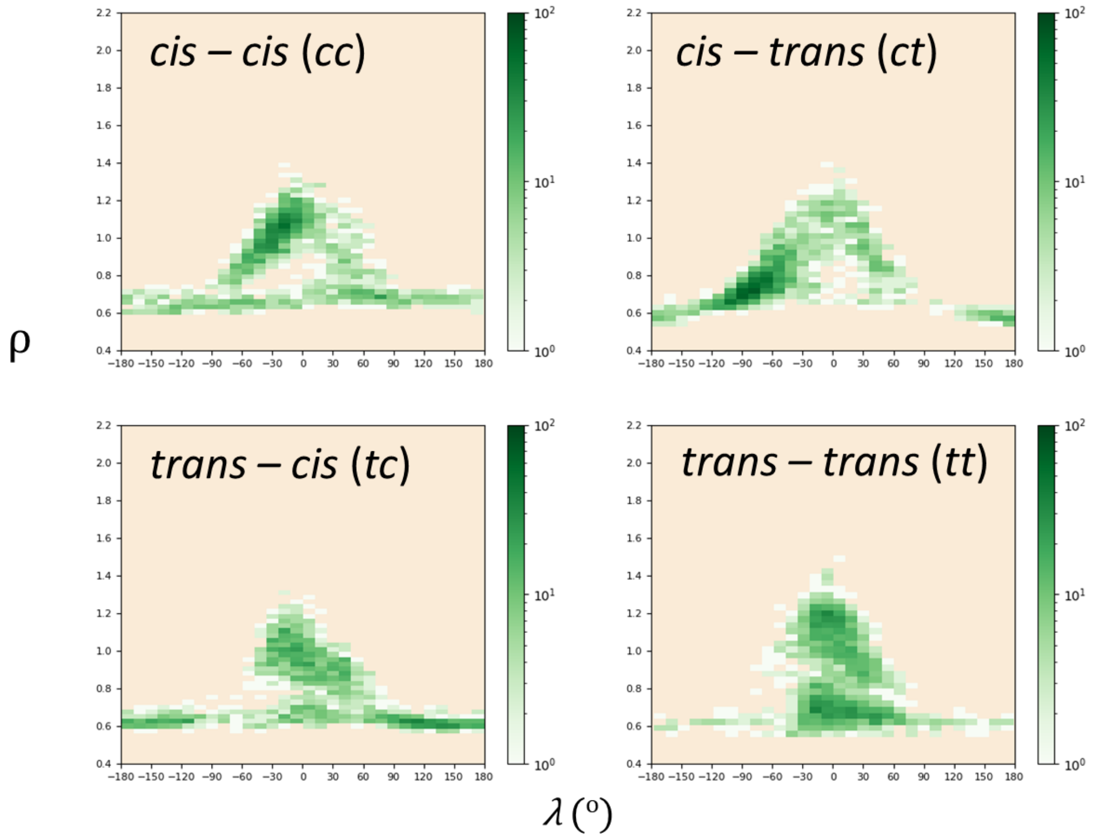

Figure S62. ρ vs. λ plots for all amide conformations (*cis – cis, cis – trans, trans – cis* and *trans – trans*) of dipeptoid Ac-Nf-Nf as obtained for a single molecule in the gas-phase using the ANI-1ccx potential (number of bins = 30).

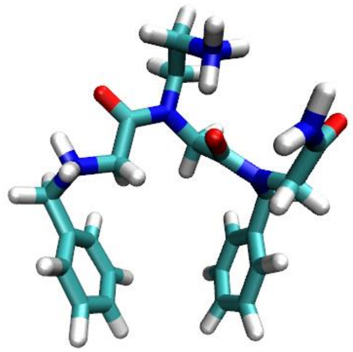

Figure S63. Nf-Nke-Nf (*ct*) example of 'gap' between aromatic rings giving rise to ρ >> 1 (here r_{ipso} = 6.62, r_{para} = 4.40 and so ρ = 1.5) whilst λ = 12.4°. The absence of such high ρ values in the gas-phase simulations implies this structuring is driven by the solvent environment.

8. Aggregation Analysis

As multiple molecules are included in the simulations and that transient aggregation is observed we deemed it appropriate to check that this aggregation did not affect the molecular structure of individual molecules. To address this point, we estimated the Solvent Accessible Surface Area (SASA) of the molecules within our system of interest using the MDTraj[34] implementation of the Shrake and Rupley algorithm,[35] on the basis that this metric is a well-established method for analyzing the degree of aggregation within self-assembly systems at various levels of theory. One way in which to interpret whether aggregation has occurred is to evaluate the ratio of initial $SASA_{init}$ to the final $SASA_{final}$ according to (Eq 1.7), which is termed the Aggregation Propensity (AP) score. In coarse-grained (CG) assembly simulations an AP score > 2.5 would indicate significant aggregation.

$$AP = \frac{SASA_{initial}}{SASA_{final}} \tag{Eq 1.7}$$

With this metric we assessed the aggregation propensity within our simulations AP and the mean AP score over the 50 ns of simulation time. In the duplicate experiments we observe AP scores ~ 0.9 – 1.0 which suggests that all molecules are well solvated, and that no significant aggregation occurred (Figures S64 – S68). Modest increases were observed for Nke-Nf-Nf, Nk-Nf-Nf and several tripeptides, as well as a sustained increase of ~ 1.5 for the dipeptide FF. To ensure this did not impact the intramolecular metrics used in this study we performed single molecule simulations and analyzed ρ, λ and η. Using Gromacs run parameters as in Section 2.3.1 omitting the NVT step, i.e., minimization, equilibration as NPT (Berendsen, v-rescaling and velocity generation) and production NPT (Parrinello-Rahman and Nose-Hoover) using Gromacs version 2020.7. For the tripeptoids the estimated η values follow the same trend as for the multimolecular studies (Figure S69 and Table 3) and qualitatively the ρ/λ plots are consistent across both studies (Figure S70 and S71). For the peptides, ρ/λ plots were also qualitatively the same, with a clear distinction between XFF and FXF sequences as found in the multimolecular studies (Figures S72).

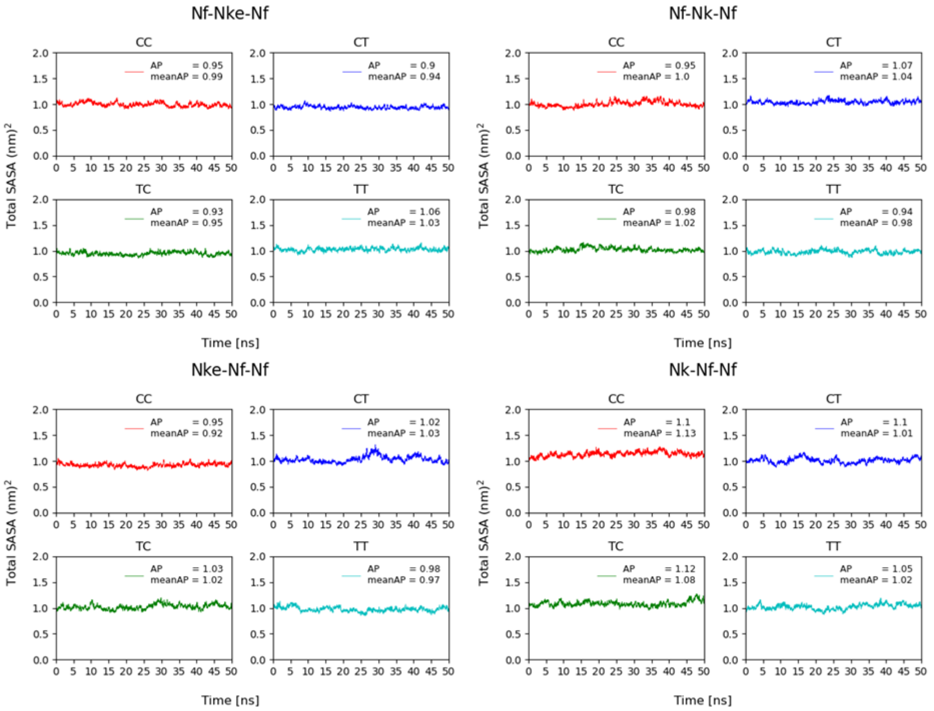

Figure S64. AP score verses time data set 1 for all tripeptoids and amide state combinations showing that this is generally very stable and non-aggregating in all cases.

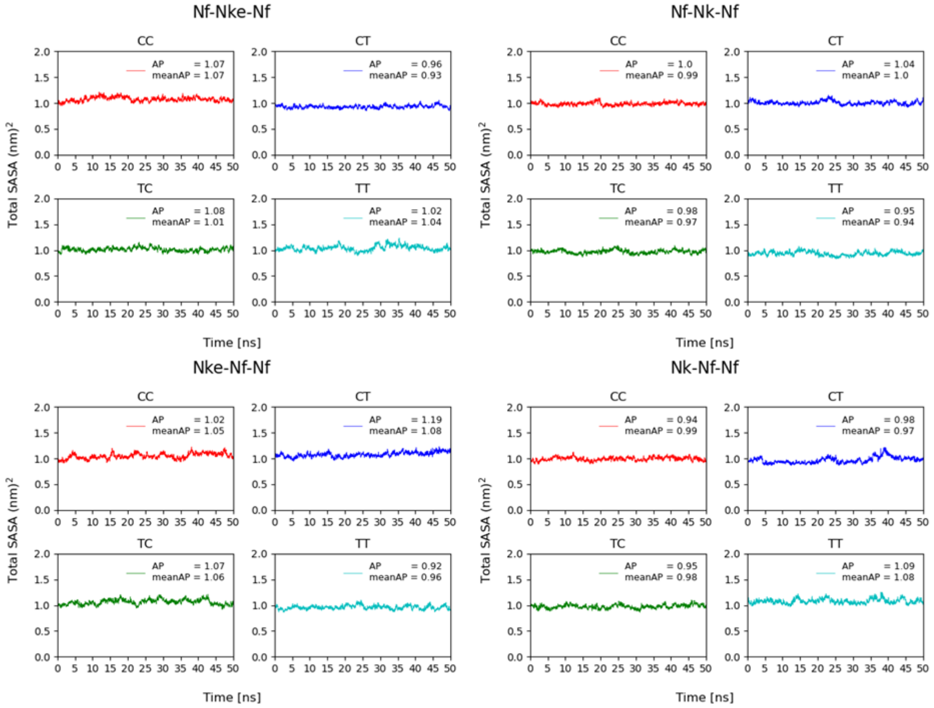

Figure S65. AP score verses time data set 2 for all tripeptoids and amide state combinations showing that this is generally very stable and non-aggregating in all cases.

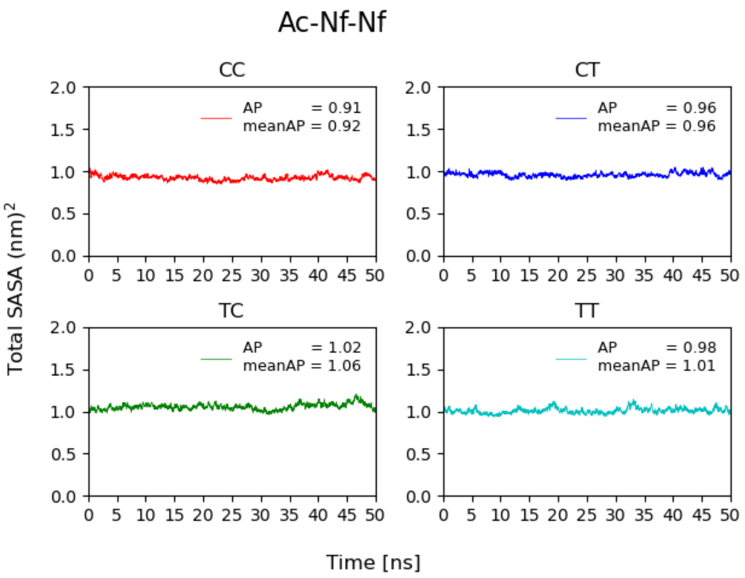

Figure S66. AP score verses time data set 1 for all amide state combinations of dipeptoid Ac-Nf-Nf and amide state combinations showing that this generally very stable and non-aggregating in all cases.

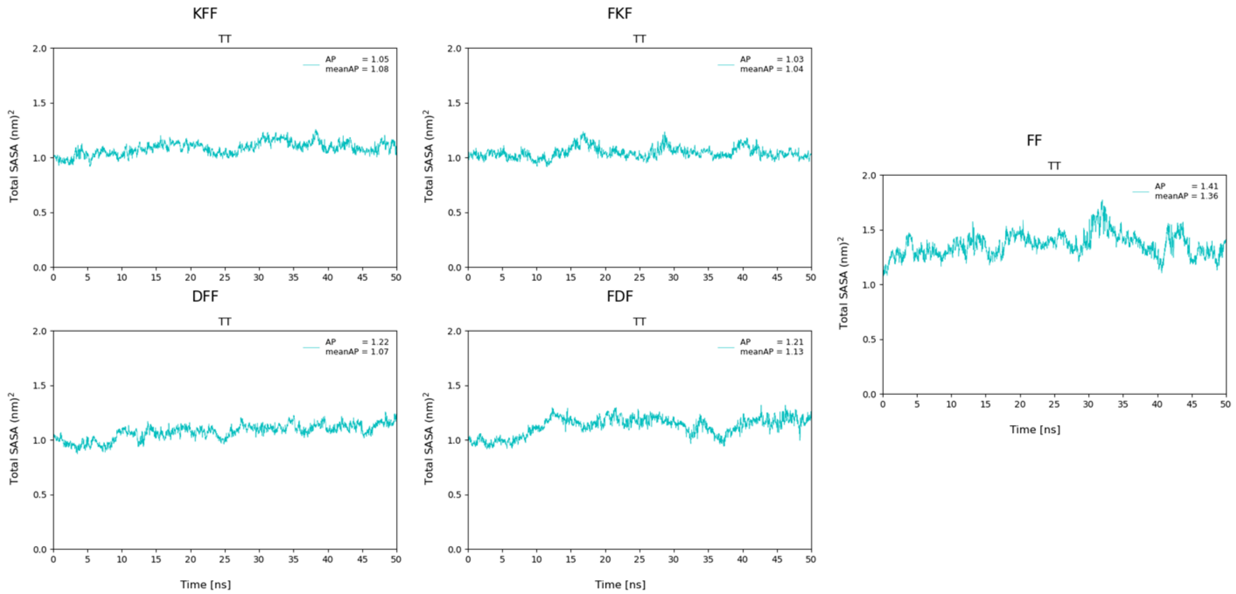

Figure S67. AP score verses time data set 1 for tripeptides KFF, FKF, DFF, FDF and dipeptide FF. These exhibit an AP score around 1 indicating no significant aggregation occurs, except for FF where aggregation is sustained over 50 ns.

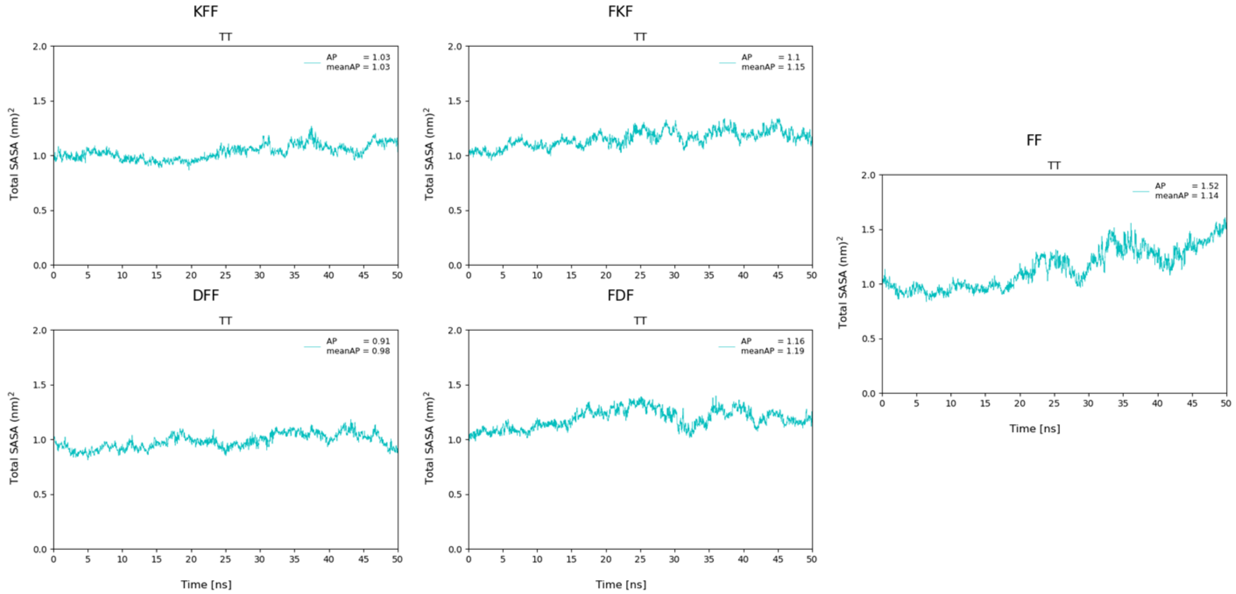

Figure S68. AP score verses time data set 2 for tripeptides KFF, FKF, DFF, FDF and FF. These exhibit an AP score around 1 indicating no significant aggregation occurs, except for FF where aggregation is sustained over 50 ns.

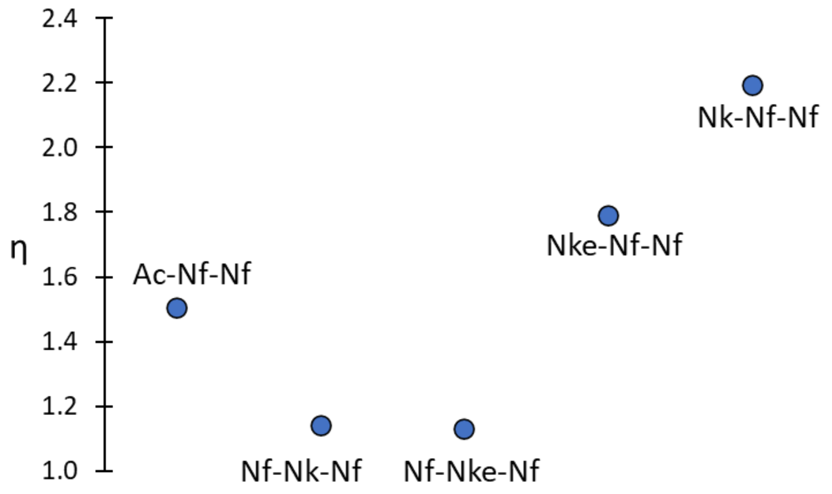

Figure S69. η values for all tripeptoids estimated from 100 ns in each amide sequence state (Table S44), following the same trend as the multimolecular simulations described in the main manuscript. The associated ρ/λ plots for are shown in Figure S70 and S71.

Table S44 – λ averages, η verses peptoid sequence and amide state

	cc	ct	tc	tt	η
**Ac-Nf-Nf**	1121.0	747.1	1126.1	995.0	1.5
**Nf-Nke-Nf**	1200.1	1148.3	1298.0	1256.1	1.1
**Nf-Nk-Nf**	1119.1	1180.7	1279.5	1277.2	1.1
**Nke-Nf-Nf**	1027.0	658.7	1180.7	798.7	1.8
**Nk-Nf-Nf**	991.3	637.9	1398.9	859.6	2.2

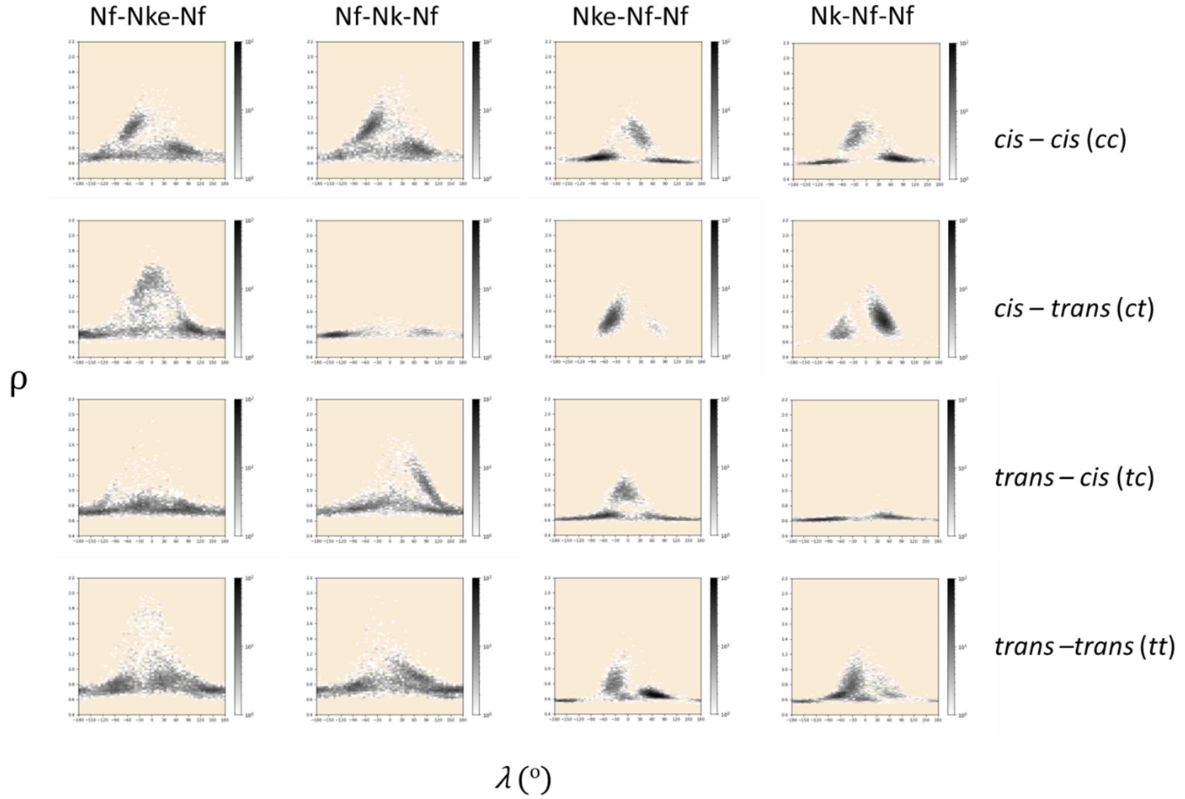

Figure S70. ρ/λ plots for single molecule simulations of Nf-Nke-Nf. Nf-Nk-Nf, Nke-Nf-Nf and Nk-Nf-Nf in TIP3P water with ions for 100 ns. These are in good agreement with the multi molecule sampling distributions shown in Section 3.1.

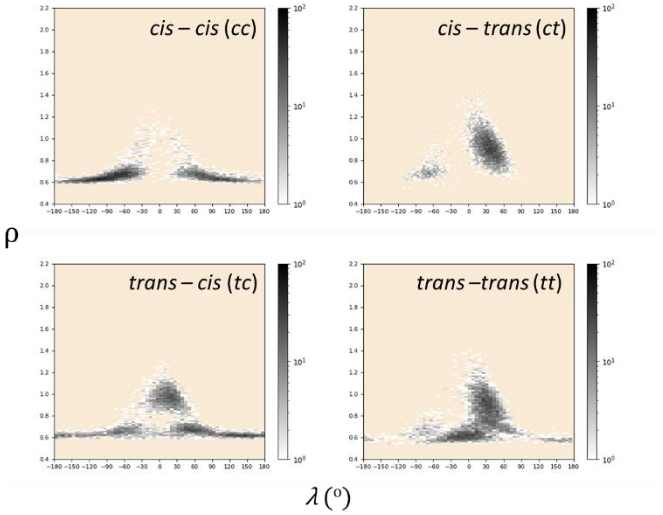

Figure S71. ρ/λ plots for single molecule simulations Ac-Nf-Nf in TIP3P water with ions for 100 ns. These are in good agreement with the multi molecule sampling distributions shown in Section 3.1.

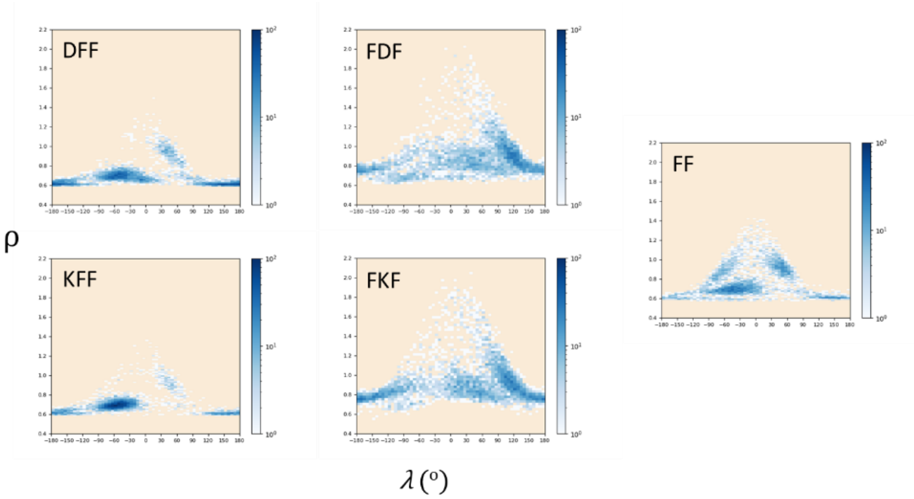

Figure S72. ρ/λ plots for single molecule simulations of DFF, FDF, KFF, FKF and FF in TIP3P water with ions for 100 ns. These are in good agreement with the multi molecule sampling distributions shown in Section 3.2.

8. References

(1) Weiser, L. J.; Santiso, E. E. A CGenFF-based force field for simulations of peptoids with both cis and trans peptide bonds. *J. Comput. Chem.* **2019**, *40* (22), 1946-1956. DOI: 10.1002/jcc.25850.
(2) *Charmm General Force Field (CGenFF) web server*. University of Maryland, 2022. https://cgenff.umaryland.edu/userAccount/userLogin.php (accessed 2021 June to December 2021).
(3) Vanommeslaeghe, K.; Hatcher, E.; Acharya, C.; Kundu, S.; Zhong, S.; Shim, J.; Darian, E.; Guvench, O.; Lopes, P.; Vorobyov, I.; et al. CHARMM General Force Field: A Force Field for Drug-Like Molecules Compatible with the CHARMM All-Atom Additive Biological Force Fields. *J. Comput. Chem.* **2010**, *31* (4), 671-690. DOI: 10.1002/jcc.21367.
(4) MacKerell, A. D.; Bashford, D.; Bellott, M.; Dunbrack, R. L.; Evanseck, J. D.; Field, M. J.; Fischer, S.; Gao, J.; Guo, H.; Ha, S.; et al. All-atom empirical potential for molecular modeling and dynamics studies of proteins. *J. Phys. Chem. B* **1998**, *102* (18), 3586-3616. DOI: DOI 10.1021/jp973084f.
(5) *Gaussian 16, Revision C.01,*; Gaussian, Inc.: Wallingford CT, 2019. (accessed.
(6) Vanommeslaeghe, K.; MacKerell, A. D. Automation of the CHARMM General Force Field (CGenFF) I: Bond Perception and Atom Typing. *J Chem Inf Model* **2012**, *52* (12), 3144-3154. DOI: 10.1021/ci300363c.
(7) Vanommeslaeghe, K.; Raman, E. P.; MacKerell, A. D. Automation of the CHARMM General Force Field (CGenFF) II: Assignment of Bonded Parameters and Partial Atomic Charges. *J Chem Inf Model* **2012**, *52* (12), 3155-3168. DOI: 10.1021/ci3003649.
(8) Mayne, C. G.; Saam, J.; Schulten, K.; Tajkhorshid, E.; Gumbart, J. C. Rapid Parameterization of Small Molecules Using the Force Field Toolkit. *J. Comput. Chem.* **2013**, *34* (32), 2757-2770. DOI: 10.1002/jcc.23422.
(9) *The Python Library Reference*; Python Software Foundation: 2020. (accessed.
(10) Mirijanian, D. T.; Mannige, R. V.; Zuckermann, R. N.; Whitelam, S. Development and Use of an Atomistic CHARMM-Based Forcefield for Peptoid Simulation. *J. Comput. Chem.* **2014**, *35* (5), 360-370. DOI: 10.1002/jcc.23478.
(11) Michaud-Agrawal, N.; Denning, E. J.; Woolf, T. B.; Beckstein, O. Software News and Updates MDAnalysis: A Toolkit for the Analysis of Molecular Dynamics Simulations. *J. Comput. Chem.* **2011**, *32* (10), 2319-2327. DOI: 10.1002/jcc.21787.
(12) O'Boyle, N. M.; Banck, M.; James, C. A.; Morley, C.; Vandermeersch, T.; Hutchison, G. R. Open Babel: An open chemical toolbox. *J Cheminformatics* **2011**, *3*. DOI: 10.1186/1758-2946-3-33.
(13) Phillips, J. C.; Braun, R.; Wang, W.; Gumbart, J. C.; Tajkhorshid, E.; Villa, E.; Chipot, C.; Skeel, R. D.; Kale, L.; K., S. Scalable molecular dynamics with NAMD. *J. Comp. Chem.* **2005**, *26*, 1781-1802. DOI: 10.1002/jcc.20289.
(14) Vermaas, J. V.; Hardy, D. J.; Stone, J. E.; Tajkhorshid, E.; Kohlmeyer, A. TopoGromacs: Automated Topology Conversion from CHARMM to GROMACS within VMD. *J Chem Inf Model* **2016**, *56* (6), 1112-1116. DOI: 10.1021/acs.jcim.6b00103.
(15) team, G. d. *Force fields in GROMACS*. Gromacs, 2023. https://manual.gromacs.org/current/user-guide/force-fields.html (accessed 2023.
(16) M. J. Abraham, T. M., R. Schulz, S. Páll, J. C. Smith, B. Hess, E. Lindahl. GROMACS: High performance molecular simulations through multi-level parallelism from laptops to supercomputers. *SoftwareX* **2015**, *1*, 19 - 25. DOI: 10.1016/j.softx.2015.06.001.

(17) Hess, B.; Bekker, H.; Berendsen, H. J. C.; Fraaije, J. G. E. M. LINCS: A linear constraint solver for molecular simulations. *J. Comput. Chem.* **1997**, *18* (12), 1463-1472. DOI: Doi 10.1002/(Sici)1096-987x(199709)18:12<1463::Aid-Jcc4>3.0.Co;2-H.
(18) Zhao, M. F.; Lachowski, K. J.; Zhang, S.; Alamdari, S.; Sampath, J.; Mu, P.; Mundy, C. J.; Pfaendtner, J.; De Yoreo, J. J.; Chen, C. L.; et al. Hierarchical Self-Assembly Pathways of Peptoid Helices and Sheets. *Biomacromolecules* **2022**, *23* (3), 992–1008. DOI: 10.1021/acs.biomac.1c01385.
(19) Butterfoss, G. L.; Renfrew, P. D.; Kuhlman, B.; Kirshenbaum, K.; Bonneau, R. A Preliminary Survey of the Peptoid Folding Landscape. *J. Am. Chem. Soc.* **2009**, *131* (46), 16798-16807. DOI: 10.1021/ja905267k.
(20) Spencer, R. K.; Butterfoss, G. L.; Edison, J. R.; Eastwood, J. R.; Whitelam, S.; Kirshenbaum, K.; Zuckermann, R. N. Stereochemistry of polypeptoid chain configurations. *Biopolymers* **2019**, *110* (6), e2326. DOI: 10.1002/bip.23266.
(21) Alamdari, S.; Torkelson, K.; Wang, X. Q.; Chen, C. L.; Ferguson, A. L.; Pfaendtner, J. Thermodynamic Basis for the Stabilization of Helical Peptoids by Chiral Sidechains. *J. Phys. Chem. B* **2023**, *127* (27), 6171-6183. DOI: 10.1021/acs.jpcb.3c01913.
(22) Moehle, K.; Hofmann, H. J. Peptides and peptoids - A quantum chemical structure comparison. *Biopolymers* **1996**, *38* (6), 781-790. DOI: 10.1002/(Sici)1097-0282(199606)38:6<781::Aid-Bip9>3.3.Co;2-A.
(23) Alamdari, S.; Pfaendtner, J. Origins of Conformational Heterogeneity in Peptoid Helices Formed by Chiral N-1-Phenylethyl Sidechains. *J. Phys. Chem. B* **2023**, *127* (27), 6163-6170. DOI: 10.1021/acs.jpcb.3c02576.
(24) Stringer, J. R.; Crapster, J. A.; Guzei, I. A.; Blackwell, H. E. Extraordinarily Robust Polyproline Type I Peptoid Helices Generated via the Incorporation of alpha-Chiral Aromatic N-1-Naphthylethyl Side Chains. *J. Am. Chem. Soc.* **2011**, *133* (39), 15559-15567. DOI: 10.1021/ja204755p.
(25) Armand, P.; Kirshenbaum, K.; Falicov, A.; Dunbrack, R. L.; Dill, K. A.; Zuckermann, R. N.; Cohen, F. E. Chiral N-substituted glycines can form stable helical conformations. *Folding & Design* **1997**, *2* (6), 369-375. DOI: 10.1016/s1359-0278(97)00051-5.
(26) Patch, J. A.; Barron, A. E. Helical peptoid mimics of magainin-2 amide. *J. Am. Chem. Soc.* **2003**, *125* (40), 12092-12093. DOI: 10.1021/ja037320d.
(27) Chongsiriwatana, N. P.; Patch, J. A.; Czyzewski, A. M.; Dohm, M. T.; Ivankin, A.; Gidalevitz, D.; Zuckermann, R. N.; Barron, A. E. Peptoids that mimic the structure, function, and mechanism of helical antimicrobial peptides. *PNAS* **2008**, *105* (8), 2794-2799. DOI: 10.1073/pnas.0708254105.
(28) Wu, C. W.; Sanborn, T. J.; Huang, K.; Zuckermann, R. N.; Barron, A. E. Peptoid oligomers with alpha-chiral, aromatic side chains: Sequence requirements for the formation of stable peptoid helices. *J. Am. Chem. Soc.* **2001**, *123* (28), 6778-6784. DOI: 10.1021/ja003154n.
(29) Sanborn, T. J.; Wu, C. W.; Zuckerman, R. N.; Barron, A. E. Extreme stability of helices formed by water-soluble poly-N-substituted glycines (polypeptoids) with alpha-chiral side chains. *Biopolymers* **2002**, *63* (1), 12-20.
(30) Pracht, P.; Grimme, S. Calculation of absolute molecular entropies and heat capacities made simple. *Chem. Sci.* **2021**, *12* (19), 6551-6568. DOI: 10.1039/D1SC00621E.
(31) Eastwood, J. R. B.; Weisberg, E. I.; Katz, D.; Zuckermann, R. N.; Kirshenbaum, K. Guidelines for designing peptoid structures: Insights from the Peptoid Data Bank. *Pept. Sci.* **2023**, *115*, e24307. DOI: 10.1002/pep2.24307.

(32) Castelletto, V.; Chippindale, A. M.; Hamley, I. W.; Barnett, S.; Hasan, A.; Lau, K. H. A. Crystallization and lamellar nanosheet formation of an aromatic dipeptoid. *Chem. Commun.* **2019**, *55* (42), 5867-5869.
(33) Gorbitz, C. H. The structure of nanotubes formed by diphenylalanine, the core recognition motif of Alzheimer's beta-amyloid polypeptide. *Chem. Commun.* **2006**, (22), 2332-2334. DOI: 10.1039/b603080g.
(34) McGibbon, R. T.; Beauchamp, K. A.; Harrigan, M. P.; Klein, C.; Swails, J. M.; Hernandez, C. X.; Schwantes, C. R.; Wang, L. P.; Lane, T. J.; Pande, V. S. MDTraj: A Modern Open Library for the Analysis of Molecular Dynamics Trajectories. *Biophys. J.* **2015**, *109* (8), 1528-1532. DOI: 10.1016/j.bpj.2015.08.015.
(35) Shrake, A.; Rupley, J. A. Environment and Exposure to Solvent of Protein Atoms - Lysozyme and Insulin. *J. Mol. Biol.* **1973**, *79* (2), 351-371. DOI: Doi 10.1016/0022-2836(73)90011-9.
